# An anesthesia-centered bundle to reduce postoperative pulmonary complications: The PRIME-AIR study protocol

**DOI:** 10.1371/journal.pone.0283748

**Published:** 2023-04-06

**Authors:** Ana Fernandez-Bustamante, Robert A. Parker, Juraj Sprung, Matthias Eikermann, Marcelo Gama de Abreu, Carlos Ferrando, B. Taylor Thompson, Marcos F. Vidal Melo

**Affiliations:** 1 Department of Anesthesiology, University of Colorado School of Medicine, Aurora, CO, United States of America; 2 Biostatistics Center, Massachusetts General Hospital, Department of Medicine, Harvard Medical School, Boston, MA, United States of America; 3 Department of Anesthesiology and Perioperative Medicine, Mayo Clinic, Rochester, MN, United States of America; 4 Department of Anesthesiology, Montefiore Medical Center, Albert Einstein College of Medicine, Bronx, NY, United States of America; 5 Department of Intensive Care and Resuscitation, Anesthesiology Institute, Cleveland Clinic, Cleveland, OH, United States of America; 6 Department of Outcomes Research, Anesthesiology Institute, Cleveland Clinic, Cleveland, OH, United States of America; 7 Department of Anesthesiology and Intensive Care, Hospital Clínic Institut D’investigació August Pi i Sunyer, Barcelona, Spain; 8 CIBER de Enfermedades Respiratorias, Instituto de Salud Carlos III, Madrid, Spain; 9 Division of Pulmonary and Critical Care, Department of Medicine, Massachusetts General Hospital, Harvard Medical School, Boston, MA, United States of America; 10 Department of Anesthesiology, Columbia University Irving Medical Center, New York, NY, United States of America; GERMANY

## Abstract

**Background:**

Postoperative pulmonary complications (PPCs) are a major cause of morbidity and mortality after open abdominal surgery. Optimized perioperative lung expansion may minimize the synergistic factors responsible for the multiple-hit perioperative pulmonary dysfunction. This ongoing study will assess whether an anesthesia-centered bundle focused on perioperative lung expansion results in decreased incidence and severity of PPCs after open abdominal surgery.

**Methods:**

Prospective multicenter randomized controlled pragmatic trial in 750 adult patients with at least moderate risk for PPCs undergoing prolonged (≥2 hour) open abdominal surgery. Participants are randomized to receive either a bundle intervention focused on perioperative lung expansion or usual care. The bundle intervention includes preoperative patient education, intraoperative protective ventilation with individualized positive end-expiratory pressure to maximize respiratory system compliance, optimized neuromuscular blockade and reversal management, and postoperative incentive spirometry and early mobilization. Primary outcome is the distribution of the highest PPC severity by postoperative day 7. Secondary outcomes include the proportion of participants with: PPC grades 1–2 through POD 7; PPC grades 3–4 through POD 7, 30 and 90; intraoperative hypoxemia, rescue recruitment maneuvers, or cardiovascular events; and any major extrapulmonary postoperative complications. Additional secondary and exploratory outcomes include individual PPCs by POD 7, length of postoperative oxygen therapy or other respiratory support, hospital resource use parameters, Patient-Reported Outcomes Measurements (PROMIS®) questionnaires for dyspnea and fatigue collected before and at days 7, 30 and 90 after surgery, and plasma concentrations of lung injury biomarkers (IL6, IL-8, RAGE, CC16, Ang-2) analyzed from samples obtained before, end of, and 24 hours after surgery.

**Discussion:**

Participant recruitment for this study started January 2020; results are expected in 2024. At the conclusion of this trial, we will determine if this anesthesia-centered strategy focused on perioperative lung expansion reduces lung morbidity and healthcare utilization after open abdominal surgery.

**Trial registration:**

ClinicalTrial.gov NCT04108130.

## Introduction

Postoperative pulmonary complications (PPCs) are a major cause of morbidity and mortality for patients undergoing the estimated 51 million annual inpatient surgeries in the US [1–3]. National estimates in 2011 suggested 1,062,000 PPCs per year, with 46,200 additional deaths, and 4.8 million additional days of hospitalization [2]. Abdominal surgery is associated with the largest absolute number of PPCs [4]. Whereas PPCs are as significant and lethal as cardiac surgical complications [1, 5], research in the field has received much less attention, and strategies to reduce perioperative lung morbidity are limited [5, 6].

Prior individual approaches to optimize specific aspects of care have been pursued to reduce PPCs, mostly focused on mechanical ventilation strategies during surgery [7–10], and the optimization of the reversal of neuromuscular blockade [11–13]. During abdominal surgery, Futier *et al*. [7] demonstrated a reduced incidence of a composite of pulmonary complications of repeated intraoperative recruitment maneuvers, higher positive end-expiratory pressure (PEEP) 6–8 cmH_2_O and lower tidal volume (VT) 6–8 ml/kg of predicted body weight (PBW) *vs*. a strategy of no lung recruitments, PEEP 0 cmH_2_O and higher VT (10–12 ml/kgPBW). The PROVHILO study found no reduction in PPCs of intraoperative constant high (12 cmH_2_O) *vs*. low (≤2 cmH_2_O) PEEP and similar protective VT 8 ml/kgPBW in patients undergoing abdominal surgery [14]. While the first trial highlighted the relevance of ventilatory strategies to postoperative outcomes, the second led to the controversial recommendation that low PEEP should be the standard of care in abdominal surgery [15], a practice anticipated to worsen lung collapse. Individualized PEEP management has been more recently explored since the PROVHILO study. Individualized PEEP titrations can optimize lung mechanics, lung aeration or oxygenation [16–18], but they have yet failed to consistently improve relevant clinical outcomes [19]. PEEP optimization in most recently published studies [16–18] is typically performed once early during the surgical procedure, and the selected PEEP level remains unchanged for the rest of surgery. However, PEEP requirements for optimal lung expansion measured as respiratory system compliance vary substantially during open abdominal surgery not only among patients, but also, within the same patient, along the different phases of surgery [20]. Therefore, a repeated PEEP titration may be a critical intraoperative intervention to effectively achieve optimized lung expansion and improve outcomes.

Other interventions during and after open abdominal surgery are likely relevant to optimize perioperative lung expansion and impact clinical outcomes. Previous reports pointed to adequate neuromuscular blockade and reversal management to improve postoperative oxygenation, minimize postoperative residual muscle weakness and optimize perioperative lung expansion [11–13]. Postoperatively, incentive spirometry followed by early ambulation are frequently employed for usual care of surgical patients as an attempt to optimize lung expansion and prevent complications after surgery [5, 21–24]. Finally, patient education has been increasingly emphasized as essential to increase engagement with adherence and quality of performance of postoperative interventions [22, 25, 26].

For the PRIME-AIR trial, we hypothesize that an intervention focused on repeated lung expansion during the perioperative period will minimize multiple and synergistic factors responsible for the multiple-hit perioperative pulmonary dysfunction and result in decreased PPCs, as well as non-pulmonary complications and healthcare utilization. To test this hypothesis, we aim to implement a bundle of perioperative interventions targeting lung expansion and composed of (1) preoperative education; intraoperative (2) repeated individualized PEEP and (3) optimized neuromuscular blockade and reversal management; and postoperative (4) incentive spirometry and (5) ambulation goals, and to assess the effect of this bundle on the incidence and severity of PPCs within postoperative day 7 as compared to usual care.

## Materials and methods

The PRIME-AIR trial protocol is presented following the Standard Protocol Items: Recommendations for Interventional Trials (SPIRIT) 2013 and 2022 guidelines [27, 28].

### Aim, design and setting

The PRIME-AIR trial is an ongoing prospective multicenter randomized controlled pragmatic trial focused on perioperative lung expansion for patients undergoing open abdominal surgery. This study aims to test if a bundle intervention focused on optimizing lung expansion before, during, and after open abdominal surgery will reduce the incidence or severity of PPCs, overall morbidity and mortality up to 90 days after surgery, and decrease plasma concentrations of biomarkers of lung injury. The trial takes place primarily at academic institutions in the United States.

### Eligibility criteria

We will study 750 adult patients of both genders undergoing open abdominal surgery and classified as at intermediate or high risk for PPCs.

Please find **inclusion and exclusion criteria** in Table 1. Eligible candidates are identified from the surgical schedule at participating sites, screened for inclusion and exclusion criteria, and then approached for introduction of the study and informed consent.

**Table 1:** Inclusion and exclusion criteria of the PRIME-AIR trial.

**Inclusion criteria**
• Adults (≥18 years) • Elective surgery with expected duration ≥2 hours • Open abdominal surgery, including: gastric, biliary, pancreatic, hepatic, major bowel, ovarian, renal tract, bladder, prostatic, radical hysterectomy, and pelvic exenteration • Intermediate or high risk of PPCs defined by an ARISCAT risk score ≥26 [4] (S1 Appendix, Table 1)
**Exclusion criteria**
• Inability or refusal to provide consent • Inability or significant difficulty to perform any study interventions, including incentive spirometry, ambulation and/or maintaining follow-up contact with study personnel for up to 90 days after the date of surgery • Participation in any interventional research study within 30 days of the time of the study • Previous surgery within 30 days prior to this study • Pregnancy • Emergency surgery • Severe obesity (above Class I, BMI≥35 kg/m^2^) • Significant lung disease: any diagnosed or treated respiratory condition that (a) requires home oxygen therapy or non-invasive ventilation (except nocturnal treatment of sleep apnea without supplemental oxygen), (b) severely limits exercise tolerance to <4 METs (e.g., patients unable to do light housework, walk flat at 4 miles/h or climb one flight of stairs), (c) required previous lung surgery, or (d) includes presence of severe pulmonary emphysema or bullae • Significant heart disease: cardiac conditions that limit exercise tolerance to <4 METs • Pulmonary hypertension • Renal failure: peritoneal or hemodialysis requirement or preoperative creatinine ≥2 mg/dL • Neuromuscular disease that impairs ability to ventilate without assistance • Severe chronic liver disease (Child-Turcotte-Pugh Score >9) • Sepsis • Malignancy or other irreversible condition for which 6-month mortality is estimated ≥20% • Bone marrow transplant

### Interventions

**Fig 1:**
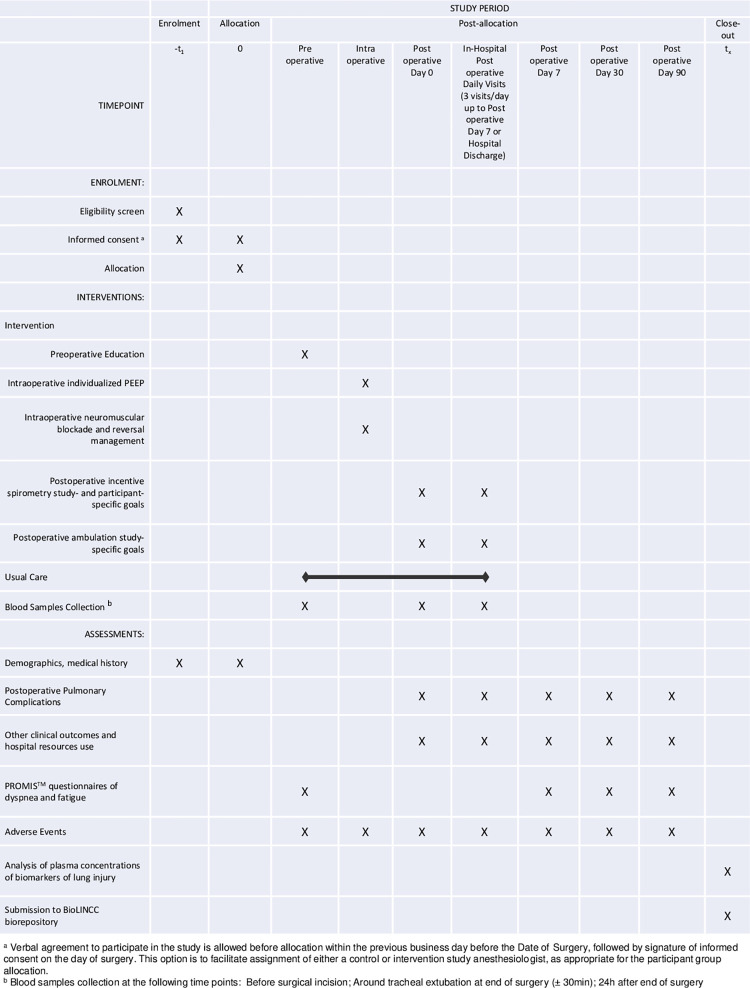
PRIME-AIR trial study workflow.

**Fig 2:**
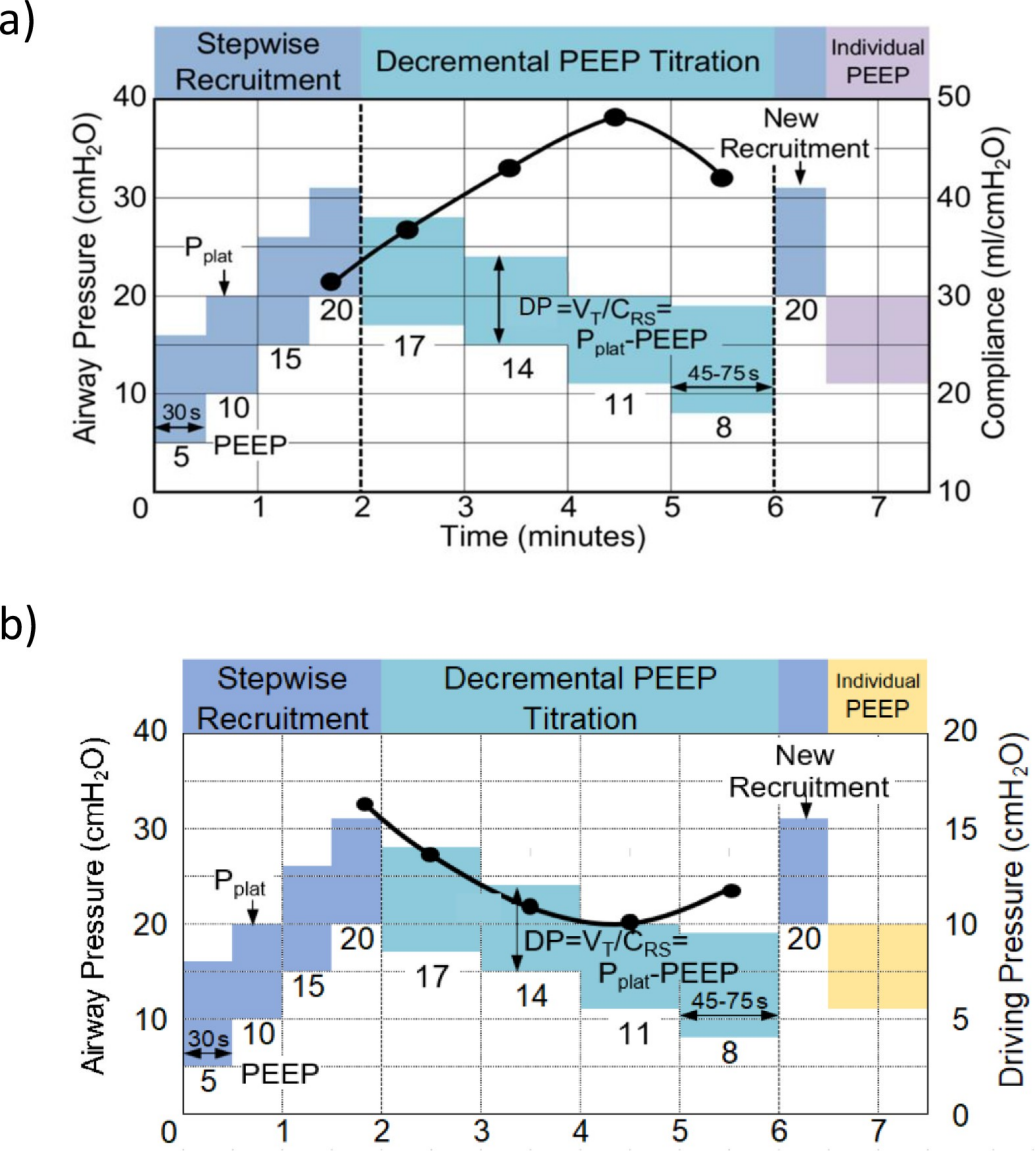
Diagram depicting intraoperative PEEP titration procedures for the intervention participants of the PRIME-AIR trial.

**Fig 3:**
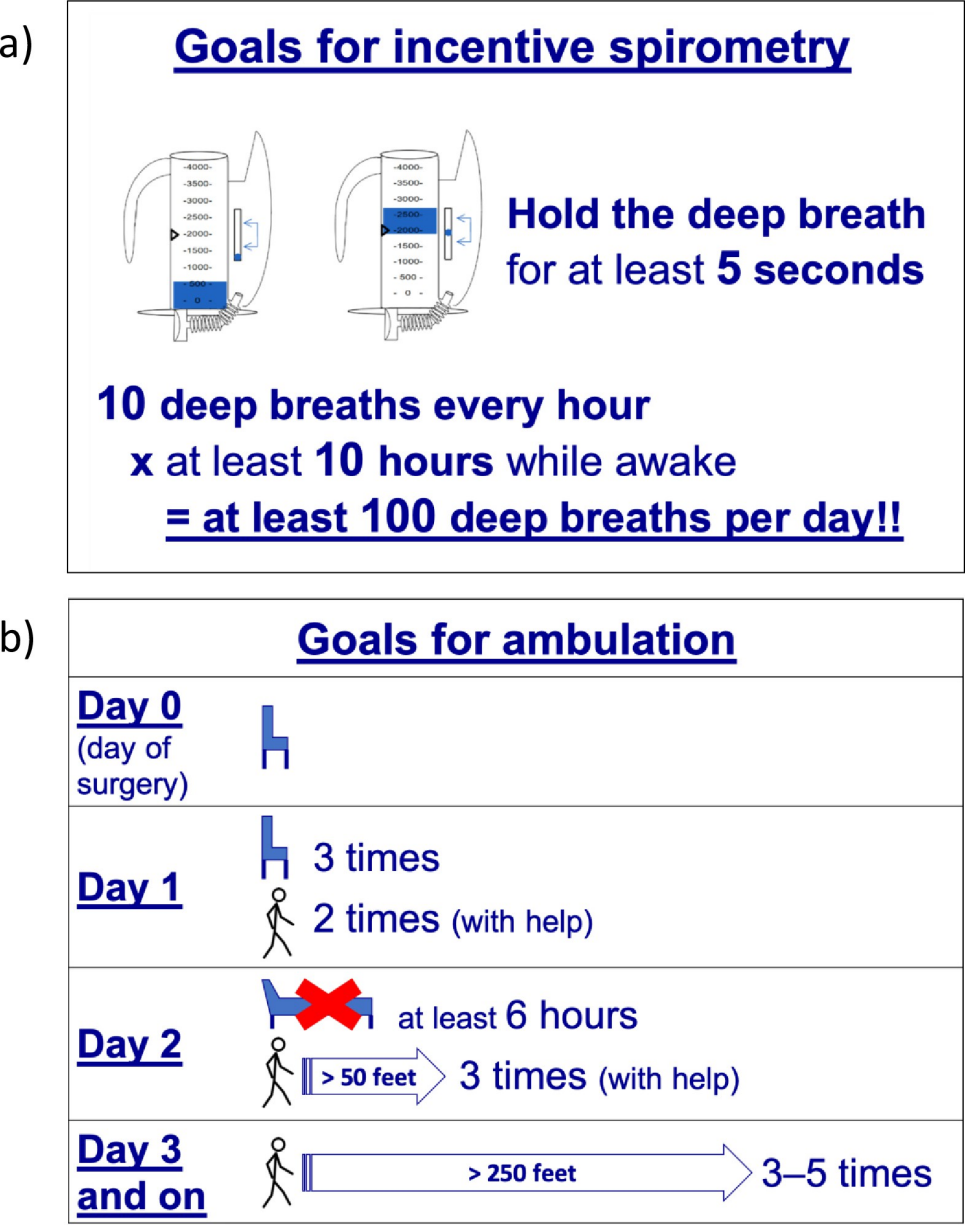
Postoperative study goals of incentive spirometry and ambulation for the intervention participants of the PRIME-AIR trial.

The study workflow diagram is shown in Fig 1. Patients are randomized to receive either usual care or an intervention bundle. Participants randomized to the usual care group receive the institution’s usual perioperative protocols of care without specific recommendations or goals. Participants randomized to the intervention group receive the intervention bundle that is summarized in the mnemonic **PRIME-AIR**: **P**EEP (positive end-expiratory pressure), **R**ecruitment, **I**ncentive spirometry, **M**uscle relaxant optimization, preoperative **E**ducation, postoperative early **A**mbulation, **I**ndividualized, and **R**einforced. Specifically, the intervention bundle consists of the following perioperative interventions aimed at optimizing perioperative lung expansion (see full details in the Protocol in S1 Appendix):

1. Preoperative education on PPCs and practice of lung expansion maneuvers. Participants randomized to the intervention group receive preoperative education in paper and video format that reviews relevance of PPCs, provides instructions for performing incentive spirometry, and includes study-specific postoperative incentive spirometry and daily ambulation goals. This preoperative education has been adapted from supported prior findings [22, 29].
2. Intraoperative periodically individualized PEEP with recruitment maneuvers to maximize respiratory system compliance (Crs). Individualized PEEP is titrated to maximize Crs following previous findings [20] confirming that this strategy achieves increased Crs, reduced driving pressure (DP) and maintains a positive end-expiratory transpulmonary pressure (Ptp_ee) during the different phases of open abdominal surgery. Mechanical ventilation for intervention patients with volume controlled ventilation and the following settings: VT 6–8 ml/kgPBW, inspiratory to expiratory (I:E) ratio 1:2, 20% inspiratory pause, respiratory rate titrated to normocapnia and FiO_2_ ≥0.4 aiming at SpO_2_ ≥96%. After confirming adequate neuromuscular blockade and hemodynamic stability, a stepwise incremental PEEP recruitment maneuver is performed (i.e., PEEP = 5, 10, 15 and 20 cmH_2_O at 30s intervals). This is followed by a 3 cmH_2_O stepwise decrease in PEEP until a maximum Crs is identified (Fig 2). After a 30s recruitment maneuver to the highest recruitment PEEP, the PEEP observed for the maximum Crs is selected. This PEEP titration is performed after tracheal intubation, hourly thereafter or sooner after any event potentially associated with lung collapse such as application of surgical retractors, disconnection of the endotracheal tube, tracheal suctioning, Trendelenburg position, or if the static respiratory system compliance is reduced by ≥15%. Deviations from these recommendations are allowed and tracked at the discretion of the anesthesiologist.
3. Intraoperative optimized neuromuscular blockade administration and reversal based on neuromuscular monitoring. Administration of intermediate-acting neuromuscular blocking agents follows appropriate dosing recommendations (see details in Table 2) and is maintained based on surgical conditions following neuromuscular transmission monitoring with 2 Hz train-of-four stimulation. At the end of surgery, neuromuscular blockade reversal is achieved following neuromuscular blockade monitoring with dosing guidelines described in Table 3 [30].
4. Postoperative incentive spirometry with study-specific daily goals and adherence enhanced by supervision by study personnel. Incentive spirometry instructions are based on the Guidelines of the American Association for Respiratory Care (AARC) [31]. Intervention patients receive: (a) specific preoperative education as described above, (b) clear expectations of incentive spirometry breaths per day (Fig 3A); and (c) direct supervision of performance by study coordinators during a target of three daily visits.
5. Postoperative ambulation with study-specific daily goals and adherence enhanced by encouragement by study personnel. Intervention patients receive: (a) preoperative education on the benefits of early mobilization and ambulation, (b) clear expectations of daily mobilization milestones (Fig 3B), (c) reinforcement and systematic monitoring of these milestones by study coordinators during three daily visits, and (d) encouraging patients to address barriers with care team.

**Table 2:** Optimized neuromuscular blockade management.

Maximal intubating and maintenance doses (mg/kg) of muscle relaxants to be used during the PRIME-AIR trial.*		
	Intubation	Maintenance
Cisatracurium (mg/kg)	0.2	0.03
Rocuronium (mg/kg)	1.2	0.2
Vecuronium (mg/kg)	0.1	0.015

* Maximal doses ± 20% considered acceptable due to usual concentrations of medication solutions.

**Table 3:** Optimized neuromuscular blockade reversal management.

Neuromuscular blockade reversal management based on monitoring of the train-of-four (TOF) ratio (T4/T1).			
Twitches in the TOF		Neostigmine dose (mcg/kg)	Sugammadex dose (mg/kg)
Quantitative Monitoring	Qualitative Monitoring		
0	0	Wait	Wait or 4–16
1	1	Wait	3–4
2–3	2–3	50	2–3
T4/T1<0.4	4 with fade	40	1–2
T4/T1 = 0.4–0.9	4 without fade	15–25	0.25–2
T4/T1>0.9		Unnecessary	Unnecessary

Intraoperative care to intervention participants is provided by anesthesiologists that have been randomized and trained in the study protocol (“intervention anesthesiologists”), while control group participants receive care by anesthesiologists that have been randomized to provide usual care (“control anesthesiologists”). Anesthesiologists are randomized at each site and may be stratified by frequency of anesthesiology practice for open abdominal surgery. Participants randomized to the control group receive usual care during surgery as per their medical anesthesia team.

Postoperative daily visits for all participants by study coordinators are maintained until hospital discharge, postoperative day 7, or when they are fully ambulatory. Full ambulation is defined as the ability to be out of bed for at least 6 hours/day and walk at least 75 meters 3 times/day. Intervention participants receive focused supervision of incentive spirometry, and review and encouragement of daily lung expansion (incentive spirometry and ambulation) study goals (Fig 3). Control participants receive daily postoperative visits with generic conversation by study coordinators.

All participants have a blood sample collected before surgery, at the end of surgery (extubation or equivalent) and 24 hours after the end of surgery. These blood samples will be used for analysis of concentrations of lung injury biomarkers and for the development of a biorepository of perioperative blood samples from patients undergoing open abdominal surgery at risk for PPCs.

#### Outcomes

All outcomes for each participant are assessed by a blinded investigator.

### Primary outcome

For an individual participant, the primary outcome is the participant’s PPC severity within postoperative day 7. We utilize an established scale of PPC severity classified from none (Grade 0) to Grade 4 [7, 32, 33] modified to include PPCs defined in recent large trials [4, 19] (Table 4). These modifications aim to maximize event detection through the addition of two items into the grading scale: (a) Mild hypoxemia (Grade 1), a usual clinical trigger for oxygen therapy in acute patients, associated by us and others with clinically meaningful outcomes and health care utilization [19, 34, 35]; and (b) Respiratory infection (Grade 2), included as an important complication in recent large studies on PPCs with a broad definition [4, 14, 36]. By using the highest PPC grade as the primary outcome in each participant, we seek to detect the impact of the bundle on both the overall PPC rate and on the severity of occurring PPCs (see Data Analysis section). The 7-day postoperative period was chosen because it includes the majority of PPCs occurring within the first month after surgery [7], has been used in previous large trials [7, 14] and is known to prolong hospital length of stay [34, 37].

**Table 4:** Definitions for postoperative pulmonary complications (PPCs) for the PRIME-AIR trial (adjusted from Futier *et al*. [7]).

**Grade 1**
Mild hypoxemia: SpO_2_ = 90–92% on room air or the equivalent imputed PaO_2_/FiO_2_ ratio when oxygen therapy is provided Mild respiratory findings: abnormal lung symptoms/signs (e.g., cough, dyspnea) and temperature >37.5°C without other documented cause; chest radiograph normal/unavailable
**Grade 2**
Cough: productive, no other cause Bronchospasm: new or pre-existent wheezing resulting in therapy change Hypoxemia: PaO_2_<60 mmHg or SpO_2_< 90% on room air or the equivalent imputed PaO_2_/FiO_2_ ratio when oxygen therapy is provided Respiratory Infection: use of antibiotic for suspected respiratory infection and at least one of the following: new or changed sputum, fever>37.5°C, WBC>12,000/mm^3^ Atelectasis*: radiological confirmation + either temperature >37.5°C or abnormal lung symptoms/signs (e.g., cough, dyspnea) Hypercarbia: transient, requiring treatment
**Grade 3**
Pleural effusion: resulting in thoracentesis Pneumonia**, suspected: radiological evidence without bacteriological confirmation Pneumonia**, proved: radiological evidence and documentation of pathological organism Pneumothorax: resulting in intervention. Ventilatory dependence: (non-invasive or invasive ventilation) < 48h
**Grade 4**
Ventilatory failure: postoperative non-invasive or invasive ventilation dependence ≥48h
* Atelectasis: lung opacification with shift of the mediastinum, hilum or hemidiaphragm towards affected area and compensatory overinflation. ** **Pneumonia:** new and/or progressive pulmonary infiltrates on chest X-ray and two or more of: fever ≥38°C or hypothermia (<36°C); white blood cell count (WBC)/mm^3^>12000 or <4000; purulent sputum and/or onset or worsening cough or dyspnea.

### Secondary outcomes

For the study include the proportion of participants with highest PPC grade as Grades 1–2 through POD 7; PPC Grades 3–4 through POD 7, 30 and 90; hypoxemia, atelectasis, pneumonia or ventilatory requirement by POD 7; intraoperative hypoxemia and rescue recruitment maneuvers; intraoperative cardiovascular events; and any major extrapulmonary postoperative complication. Secondary outcomes for the study also include the median length of postoperative oxygen therapy or other respiratory support and length of hospital stay. See details and definitions in the Protocol in S1 Appendix.

Additional **Exploratory Outcomes** are listed in the Protocol in S1 Appendix, and encompass mortality, frequency of individual pulmonary and extra-pulmonary complications, residual muscle weakness, doses of intraoperative vasoactive medications and fluids, other hospital resources used (e.g., unplanned ICU admission, post-discharge disposition, readmissions), and PROMIS® questionnaire scores.

### Plasma concentrations of biomarkers

To test the hypothesis that the PRIME-AIR bundle intervention will minimize lung mechanical injury and inflammation by reducing overinflation and atelectasis, we will compare plasma biomarker concentrations in the PRIME-AIR intervention *vs*. control groups before, at the end of and 24h after surgery. We will include plasma biomarkers of inflammation (cytokines IL-6, IL-8), epithelial injury (the soluble form of the receptor for advanced glycation end-products, RAGE, and club cell protein 16, CC16), and endothelial injury (angiopoietin-2, Ang-2) [38–40]. Blood collection in this aim will not only allow for the biomarkers analysis, but also for the creation of a biobank for future studies on PPCs.

### Participant timeline

Patient enrollment is currently ongoing and expected to reach the 750 studied patient goal no later than summer 2023.

### Sample size

Sample size was estimated using simulations of the primary analysis of the distribution of highest PPC severity between the two groups. Based on these simulations, our sample size of 375/group (750 total) being studied has 93% power (α = 0.05, two-sided) for the primary analysis for the study. We formulated the treatment effect in this project as a relative percent reduction in PPC rate between groups, not an absolute difference between the two groups. For simplicity in these simulations, we assumed no change in the distribution of the severity of PPCs, so the reduction in PPC rate applied to the number of PPCs of each severity grade. The number of participants without any PPC (highest severity PPC grade 0) was then adjusted accordingly. We then assumed a PPC event rate in the usual care group of 40% [7, 14, 19, 34], and a treatment effect (relative reduction of PPC rate) of 35.29% (so an absolute PPC event rate of 25.88% in the group receiving the intervention bundle). The 35.29% reduction in the rate of any PPCs was the lower 10% bound (one-sided) of the highest posterior region based on previous studies using a Bayesian analysis. This means that we have a 90% expectation that the true treatment effect (reduction in the overall rate of PPCs) of the entire bundle would be greater than 35.29%. We assumed for the simulations that among participants with PPCs, approximately 50% would be grade 1, 25% grade 2, 20% grade 3, and 5% grade 4.

### Recruitment

Potential candidates are identified from the surgical schedule at individual sites, based on type and estimated duration of surgical procedure. Medical records are reviewed to ensure that participants meet inclusion/exclusion criteria. Candidates are then approached for introduction of the PRIME-AIR trial by phone, videoconference (telehealth), or in person during a preoperative hospital visit. All research personnel approaching potential participants have appropriate human subjects research training per institutional requirements (e.g., Collaborative Institutional Training Initiative (CITI Program) training) and study training.

### Allocation

Randomization is performed through the PRIME-AIR online randomization system (RS). Randomization at each site is blocked over time. Only site is used as a stratification factor.

### Blinding

The intraoperative and postoperative study interventions cannot be blinded. At each site, an unblinded investigator performs the participant’s randomization, education, and postoperative incentive spirometry/mobilization encouragement during hospital visits. An anesthesiologist assigned to the appropriate group implements the intervention in the operating room. An investigator blinded to patient allocation assesses PPCs and all other participant outcomes with information obtained from the participant’s medical chart and the participant via phone calls, videoconference or in person visits. Investigators performing biomarker assays will also be blinded to study group allocation.

### Data collection

These include preoperative demographics, comorbidities, details related to the surgical procedure (e.g., duration, type), vital signs, periodic ventilator settings, medications and fluids received during surgery, parameters related to the postoperative course (e.g., admission to the Intensive Care Unit, length of stay, clinical events within postoperative days 7, 30 and 90) and Patient-Reported Outcomes Measurement Information System (PROMIS®) short questionnaires of fatigue (Short Form 13a) and dyspnea (Short Form 10a) preoperatively and on postoperative days 7, 30 and 90. Details and definitions are included in the Protocol in S1 Appendix. Data collection (directly from participants and from their electronic medical records) is required for any participants that experience a deviation from the bundle intervention unless they withdraw their consent to data collection.

### Data management

Data is entered in the PRIME-AIR StudyTRAX database (ScienceTrax, LLC, Macon GA) which consists of separate projects for screening, unblinded data collection and blinded data collection. Access to specific projects is restricted to trained study team members at the site filling the role. Access is also available to members of the Statistical and Data Coordinating Center. Data is queried for completeness and validity. Missing data is minimized by allowing collection from medical records for certain variables. As we anticipate missing data on less than 1% of participants for the primary endpoint, no data imputation for missing data will be done.

### Data analysis plan

The primary analysis will use a Cochran-Mantel Haenszel test (specifically the row mean score test), stratifying by site. This tests the null hypothesis that there is no difference between the two treatment groups in the distribution of the highest PPC grade during the first seven days of the study. The alternative hypothesis is that there is a difference between the two treatment groups in the distribution of the most severe PPC grade during the first seven days of the study. The results of this test will determine whether the study can claim that the intervention bundle reduced the average level of PPC severity. Secondary outcomes will be analyzed depending on the type of variable (see Table 6 of Protocol in S1 Appendix). More complex modeling approaches will be used to explore the results in more detail.

### Data monitoring

A Data Safety Monitoring Board (DSMB) has been established by the Sponsor, the National Heart, Lung and Blood Institute at the National Institutes of Health (NIH/NHLBI), and a Medical Monitor has been appointed by the study’s Executive Committee. The DSMB and the Medical Monitor are independent of the Study Management Committee and the Sponsor. The Medical Monitor receives information regarding all Adverse Events and provides advice on their possible relatedness to the study. The DSMB met prior to start of study to review and approve the protocol and approves all protocol amendments.The DSMB meets every 6 months during study enrollment with the Medical Monitor and the Study Management Committee. During these meetings, the DSMB receives updated reports of study data, interim data analyses as planned, and safety information (e.g., Adverse Events). Serious Adverse Events are reported to the DSMB within 24 hours (fatal events) or within 7 days (nonfatal events) of their notification to the Statistical and Data Coordinatng Center.

### Interim analysis

Interim analysis uses a Haybittle-Peto boundary (P<0.001) for the primary endpoint. Data is analyzed as described above and provided to the DSMB. There will be two interim analyses for efficacy (at approximately 250 and 500 participants with available data), and a futility analysis was planned for the second timepoint.

### Self-auditing

Sites engage in a self-assessment with at least 5 participants (or 10% of those recruited by the respective site), where a coordinator not involved in the primary data collection re-extracts the data from the EMR. Data is checked for consistency between the primary data collected and the self-assessment. Discrepancies are resolved by the site PI. Depending on the results of this review process, the Study Management Committee decides if data quality is adequate or not, in which case they can request a self-assessment of 5 additional participants from that site or an internal review (by the site) of all their data of a particular type. If there were major issues, a source document verification, remote monitoring or even an in-person visit may be recommended.

### Research ethics approval

The Protocol and informed consent forms included in the Appendix have been approved by the Partners Human Research Institutional Review Board (protocol# 2019P001788, original approval date 8/20/2019). The Partners Human Research Institutional Review Board acts as the single Institutional Review Board (sIRB) for all sites except for a Veterans Administration (VA) facility reviewed by their own IRB. The single IRB approved the protocol, site-specific informed consent forms, materials for participant recruitment, and study participant documents (e.g., preoperative education, postoperative study milestones). Annual reviews and any protocol modifications are also submitted for review and approval by the sIRB and the Miami VA IRB. Routine reports to the sIRB include the total number of participants enrolled, summaries of participants characteristics, outcomes, and adverse events, and summaries of each DSMB review of safety and/or efficacy.

### Protocol amendments

Formal amendments to the protocol will be pursued for any modifications to the protocol affecting participant eligibility, study objectives or any significant administrative study aspect or study procedure. Any protocol amendments considered necessary by the Study Management Committee or requested by the DSMB will be approved by such DSMB and the appropriate IRB(s) prior to the implementation of any changes. An important protocol adjustment was obtained in the spring of 2020 to allow minimizing in person contact for the extenuating circumstances surrounding the COVID-19 pandemic [41]. The COVID-19 pandemic caused a substantial impact on the PRIME-AIR trial due to delays and cancellations of elective surgical procedures and unprecendented efforts to minimize transmission of viral infections between patients, healthcare personnel and clinical research members. The PRIME-AIR trial had to pause enrollment from March 2020 to July 2020. Recruitment resumed when permitted by each site. Protocol study modifications were guided by individual institutional recommendations and reviewed and approved by the sIRB in June 2020. These modifications included electronic informed consent processes and different types of Health Insurance Portability and Accountability Act (HIPPA)-approved virtual contact strategies (institutional tele-health or videoconference platforms) between study participants and investigators.

### Consent or assent

Participant identification and screening may be done from pre-admission/anesthesia clinic schedules (manual or electronic), surgeons’ office schedules, operating room schedules and by communication with relevant nursing and medical staff, depending on site preferences. Once subjects are identified as meeting inclusion and exclusion criteria, they are contacted according to each site’s procedures including approaches such as in person during hospital clinic visits, phone call or videoconference through a site-specific approved HIPAA-compliant telemedicine videoconferencing platform. Research personnel (research coordinators or research nurses) at each site introduces the PRIME-AIR trial to the subject. If the subject is interested in receiving further information, the site provides a written consent containing that information in person, by mail, or email. A follow-up telephone call is done to discuss any questions or concerns the subject may have. After those are addressed and if the subject agrees to participate, a signed informed consent is obtained in person or by a site-specific HIPPA-compliant eConsent process. If none of these options are feasible until the day of surgery, the sIRB has allowed that the subject has the option to verbally agree to participate and sign the informed consent on the day of surgery. This verbal agreement to participate is needed to allow randomization of study participants before the day of surgery and assign an anesthesiologist for the appropriate study group. An independent consent section is required for the sharing of biological specimens and data with the NHLBI BioLINCC repository.

### Confidentiality

Study-related information is stored at each study site following secure local and sIRB-guided regulations for clinical research data, with participant protected health information stored in locked file cabinets in areas with access restricted to study personnel. All participant data collection reports and laboratory specimens are identified by a study-specific identification code number to maintain participant confidentiality. The individual sites but not the Statistical and Data Coordinating Center have access to participants names and contact information. Study data from all participants is entered by site study personnel using the participant’s study code number into a central, study-specific and password-protected commercial online database (StudyTRAX) provided by the Statistical and Data Coordinating Center. Dates and site name are included in the PRIME-AIR online database. No identifiable information is shared in any reports from the Statistical and Data Coordinating Center, communications to the sIRB or DSMB, or any publications.

### Access to data

The Statistical and Data Coordinating Center will oversee the data sharing process until data sets are submitted to the NIH/NHLBI repository. Site investigators always have access to their own data. Primary data is stored in the StudyTRAX data managment system, which limits access to authorized users for specific sites / types of data (e.g. blinded coordinators only have access to the blinded and screening data at their own site). The Clinical Coordinating Principal Investigators will have access to all cleaned data for the study; this would be provided in password protected files of an appropriate format (e.g. SAS datasets) as requested by the individual. Writing groups for specific papers will have access to the data used in the manuscript (again, password protected, in a format agreed with the writing group) To ensure confidentiality, data from multiple sites shared with writing groups and other study team members outside the Study Management Committee will be deidentified of any PHI, with date of surgery specified by month/year only, and all other dates converted to days before/after surgery.

We will comply with all data sharing and dissemination requirements on the NIH Policy on the Dissemination of NIH-Funded Clinical Trial Information (Notice Number: NOT-OD-16-149). This includes registration of the study in the required time frame and submitting results to ClinicalTrials.gov within 12 months of the date of final collection for the primary endpoint as required by these regulations. We will submit our data and specimens to the NHLBI BioLINCC repository (or a successor repository) for those participants that opted to participate in this NHLBI BioLINCC biorepository. Our data and specimen sharing plan will be consistent with NIH and NHLBI guidelines in effect at the time we are preparing the transfer.

### Ancillary and post-trial care

All intervention components of the PRIME-AIR trial are included within the standard of care principles for surgical patients. If any incidental findings are discovered from participants’ interactions with the research team during any phases of the study, they will be communicated to the participant’s primary medical team as appropriate for further care.

### Dissemination policy

We will prioritize timely dissemination of the results of the PRIME-AIR study to ensure that results are widely available for clinical practice. The Scientific Dissemination Committee will direct the dissemination activities and determine authorship eligibility following accepted medical peer-review guidelines [42]. No professional writers will be used. Three levels of dissemination are planned, including: 1) health care providers, 2) the scientific community, and 3) the general public. We have already opened a dedicated website (www.primeairstudy.org). The PRIME-AIR study website will initially contain information on study goals, study design, participating institutions and scientists. After the results have been published, the website will contain the detailed results of the PRIME-AIR study. Results will also be presented at medical local, national and international meetings. We anticipate the submission of the primary manuscript within a few months after the last visit of the last patient, and multiple secondary manuscripts containing a priori secondary analyses subsequently.

We will also comply with all requirements on the NIH Policy on the Dissemination of NIH-Funded Clinical Trial Information, which includes registration of the study and submitting results to ClinicalTrials.gov within the appropriate time frames. We will submit our data and specimens to the NHLBI BioLINCC repository and follow their required procedures at the end of the study.

## Discussion

The PRIME-AIR trial has been designed to address the primary hypothesis of whether a perioperative lung expansion bundle intervention can reduce the frequency and/or severity of PPCs within postoperative day 7 after ≥2h-long open abdominal surgery in patients with at least moderate risk for PPCs, compared to usual care. Reducing the pulmonary morbidity is a critical need for patients after open abdominal surgery. The potential of the PRIME-AIR study to improve pulmonary outcomes with interventions that are well known and do not increase healthcare costs has a clear clinical value. Importantly, the PRIME-AIR trial will provide very granular information related to the frequency and severity of PPCs in academic medical centers. Data will include mild complications that have not been studied prospectively in detail (e.g., mild hypoxemia, mild respiratory symptoms) and their association with healthcare resources use and outcomes up to 3 months after surgery.

The PRIME-AIR bundle intervention includes preoperative patient education, intraoperative PEEP titration and optimized neuromuscular blockade and reversal management, and postoperative incentive spirometry and early ambulation. These pragmatic interventions constitute best care practices of perioperative care and do not introduce new procedures, but they emphasize and reinforce strategies already familiar to clinicians. Detailed data collection from the PRIME-AIR trial will provide insight on the effect of the individual bundle components on postoperative pulmonary outcomes. This is particularly important because the implementation of these best care practices is time consuming to healthcare personnel and is inconsistently followed by providers and patients.

Of note, findings from the PRIME-AIR trial will be valuable independent of the primary results. This large multicenter study will provide comprehensive information on patients undergoing open abdominal surgery from varied academic US medical centers to characterize in-depth not only pulmonary complications up to 90 days after surgery, but also extra-pulmonary outcomes and validated Patient-Reported Outcome Measurements (PROMIS®). We will obtain extensive details about key aspects of care process for abdominal surgery at US academic centers, including those pertaining to anesthesia-centered perioperative care (e.g., neuromuscular blockade and reversal, analgesia strategies and opioid consumption), as well as postoperative care and hospital resources use after abdominal surgery (e.g., incentive spirometry and ambulation, hospital length of stay or readmissions up to 90 postoperative days). In addition, we will understand details related to patient adherence with postoperative lung expansion recommendations of incentive spirometry and ambulation. The analysis of plasma concentrations of accepted lung injury biomarkers may suggest mechanistic pathways associated with perioperative lung expansion and postoperative pulmonary or extra-pulmonary complications. Finally, the PRIME-AIR trial has consolidated the creation of the Perioperative Research Network (PRN), a diverse group of academic anesthesiologists and investigators focused on perioperative research and outcomes. Various secondary analyses and ancillary studies have been discussed and are being planned for additional questions that benefit from the original dataset.

It is possible that results from the PRIME-AIR trial cannot be generalized to other surgical populations with lower risk for PPCs. The PRIME-AIR study does not enroll patients undergoing emergency, elective laparoscopic or short open abdominal or non-abdominal surgical procedures. Therefore study findings will have to be confirmed in those populations separately. Also, the effectiveness of the individual PRIME-AIR bundle intervention components may depend on quality of implementation of perioperative intervention components and on participant’s adherence to postoperative study goals. Data related to those variables (e.g., quality of implementation, patient adherence) are collected during the trial to provide an assessment of the impact of these aspects on the incidence and severity of PPCs. Lastly, the participating sites are all large US academic centers. As a consequence, usual care in those institutions may not represent the typical care in community hospitals thoughout the nation or outside the US.

Results from the ongoing PRIME-AIR study are expected to be available in 2024. We are confident that the PRIME-AIR trial will provide a wealth of information relevant to postoperative pulmonary mobidity after abdominal surgery.

## Supporting information

S1 Checklist SPIRIT checklist (combined SPIRIT 2013 and SPIRIT-Outcomes 2022 items). (DOCX)

S1 Appendix (PDF)

## References

[1] Lawrence VA, Hilsenbeck SG, Mulrow CD, Dhanda R, Sapp J, Page CP. Incidence and hospital stay for cardiac and pulmonary complications after abdominal surgery. Journal of general internal medicine. 1995;10(12):671–8. Epub 1995/12/01. doi: 10.1007/BF02602761 . 8770719

[2] Shander A, Fleisher LA, Barie PS, Bigatello LM, Sladen RN, Watson CB. Clinical and economic burden of postoperative pulmonary complications: patient safety summit on definition, risk-reducing interventions, and preventive strategies. Crit Care Med. 2011;39(9):2163–72. Epub 2011/05/17. doi: 10.1097/CCM.0b013e31821f0522 . 21572323

[3] Fernandez-Perez ER, Sprung J, Afessa B, Warner DO, Vachon CM, Schroeder DR, et al. Intraoperative ventilator settings and acute lung injury after elective surgery: a nested case control study. Thorax. 2009;64(2):121–7. Epub 2008/11/08. doi: 10.1136/thx.2008.102228 . 18988659

[4] Canet J, Gallart L, Gomar C, Paluzie G, Valles J, Castillo J, et al. Prediction of postoperative pulmonary complications in a population-based surgical cohort. Anesthesiology. 2010;113(6):1338–50. Epub 2010/11/04. doi: 10.1097/ALN.0b013e3181fc6e0a . 21045639

[5] Lawrence VA, Cornell JE, Smetana GW, American College of P. Strategies to reduce postoperative pulmonary complications after noncardiothoracic surgery: systematic review for the American College of Physicians. Ann Intern Med. 2006;144(8):596–608. Epub 2006/04/19. doi: 10.7326/0003-4819-144-8-200604180-00011 . 16618957

[6] Qaseem A, Snow V, Fitterman N, Hornbake ER, Lawrence VA, Smetana GW, et al. Risk assessment for and strategies to reduce perioperative pulmonary complications for patients undergoing noncardiothoracic surgery: a guideline from the American College of Physicians. Ann Intern Med. 2006;144(8):575–80. Epub 2006/04/19. doi: 10.7326/0003-4819-144-8-200604180-00008 . 16618955

[7] Futier E, Constantin JM, Paugam-Burtz C, Pascal J, Eurin M, Neuschwander A, et al. A trial of intraoperative low-tidal-volume ventilation in abdominal surgery. N Engl J Med. 2013;369(5):428–37. doi: 10.1056/NEJMoa1301082 . 23902482

[8] Ladha K, Vidal Melo MF, McLean DJ, Wanderer JP, Grabitz SD, Kurth T, et al. Intraoperative protective mechanical ventilation and risk of postoperative respiratory complications: hospital based registry study. BMJ. 2015;351:h3646. doi: 10.1136/bmj.h3646 . 26174419 PMC4501577

[9] de Jong MAC, Ladha KS, Vidal Melo MF, Staehr-Rye AK, Bittner EA, Kurth T, et al. Differential Effects of Intraoperative Positive End-expiratory Pressure (PEEP) on Respiratory Outcome in Major Abdominal Surgery Versus Craniotomy. Ann Surg. 2016;264(2):362–9. Epub 2015/10/27. doi: 10.1097/SLA.0000000000001499 ; PubMed Central PMCID: PMC4841734. 26496082 PMC4841734

[10] Fernandez-Bustamante A, Wood CL, Tran ZV, Moine P. Intraoperative ventilation: incidence and risk factors for receiving large tidal volumes during general anesthesia. BMC Anesthesiol. 2011;11:22. Epub 2011/11/23. doi: 10.1186/1471-2253-11-22 ; PubMed Central PMCID: PMC3235523. 22103561 PMC3235523

[11] Grosse-Sundrup M, Henneman JP, Sandberg WS, Bateman BT, Uribe JV, Nguyen NT, et al. Intermediate acting non-depolarizing neuromuscular blocking agents and risk of postoperative respiratory complications: prospective propensity score matched cohort study. BMJ. 2012;345:e6329. Epub 2012/10/19. doi: 10.1136/bmj.e6329 ; PubMed Central PMCID: PMC3473088. 23077290 PMC3473088

[12] Sasaki N, Meyer MJ, Malviya SA, Stanislaus AB, MacDonald T, Doran ME, et al. Effects of neostigmine reversal of nondepolarizing neuromuscular blocking agents on postoperative respiratory outcomes: a prospective study. Anesthesiology. 2014;121(5):959–68. Epub 2014/09/17. doi: 10.1097/ALN.0000000000000440 . 25225821

[13] Fuchs-Buder T, Nemes R, Schmartz D. Residual neuromuscular blockade: management and impact on postoperative pulmonary outcome. Curr Opin Anaesthesiol. 2016;29(6):662–7. Epub 2016/10/19. doi: 10.1097/ACO.0000000000000395 . 27755128

[14] Prove Network Investigators for the Clinical Trial Network of the European Society of Anaesthesiology, Hemmes SN, Gama de Abreu M, Pelosi P, Schultz MJ. High versus low positive end-expiratory pressure during general anaesthesia for open abdominal surgery (PROVHILO trial): a multicentre randomised controlled trial. Lancet. 2014;384(9942):495–503. doi: 10.1016/S0140-6736(14)60416-5 . 24894577 PMC6682759

[15] Guldner A, Kiss T, Serpa Neto A, Hemmes SN, Canet J, Spieth PM, et al. Intraoperative protective mechanical ventilation for prevention of postoperative pulmonary complications: a comprehensive review of the role of tidal volume, positive end-expiratory pressure, and lung recruitment maneuvers. Anesthesiology. 2015;123(3):692–713. doi: 10.1097/ALN.0000000000000754 . 26120769

[16] Pereira SM, Tucci MR, Morais CCA, Simoes CM, Tonelotto BFF, Pompeo MS, et al. Individual Positive End-expiratory Pressure Settings Optimize Intraoperative Mechanical Ventilation and Reduce Postoperative Atelectasis. Anesthesiology. 2018;129(6):1070–81. Epub 2018/09/28. doi: 10.1097/ALN.0000000000002435 . 30260897

[17] Zhang C, Xu F, Li W, Tong X, Xia R, Wang W, et al. Driving Pressure-Guided Individualized Positive End-Expiratory Pressure in Abdominal Surgery: A Randomized Controlled Trial. Anesth Analg. 2021. Epub 2021/06/15. doi: 10.1213/ANE.0000000000005575 . 34125080

[18] Wu XZ, Xia HM, Zhang P, Li L, Hu QH, Guo SP, et al. Effects of ultrasound-guided alveolar recruitment manoeuvres compared with sustained inflation or no recruitment manoeuvres on atelectasis in laparoscopic gynaecological surgery as assessed by ultrasonography: a randomized clinical trial. BMC Anesthesiol. 2022;22(1):261. Epub 20220816. doi: 10.1186/s12871-022-01798-z ; PubMed Central PMCID: PMC9380300. 35974310 PMC9380300

[19] Ferrando C, Soro M, Unzueta C, Suarez-Sipmann F, Canet J, Librero J, et al. Individualised perioperative open-lung approach versus standard protective ventilation in abdominal surgery (iPROVE): a randomised controlled trial. Lancet Respir Med. 2018;6(3):193–203. Epub 2018/01/27. doi: 10.1016/S2213-2600(18)30024-9 . 29371130

[20] Fernandez-Bustamante A, Sprung J, Parker RA, Bartels K, Weingarten TN, Kosour C, et al. Individualized PEEP to optimise respiratory mechanics during abdominal surgery: a pilot randomised controlled trial. British journal of anaesthesia. 2020;125(3):383–92. Epub 2020/07/20. doi: 10.1016/j.bja.2020.06.030 ; PubMed Central PMCID: PMC7497030. 32682559 PMC7497030

[21] Yanez-Brage I, Pita-Fernandez S, Juffe-Stein A, Martinez-Gonzalez U, Pertega-Diaz S, Mauleon-Garcia A. Respiratory physiotherapy and incidence of pulmonary complications in off-pump coronary artery bypass graft surgery: an observational follow-up study. BMC pulmonary medicine. 2009;9:36. Epub 2009/07/30. doi: 10.1186/1471-2466-9-36 ; PubMed Central PMCID: PMC2727489. 19638209 PMC2727489

[22] Cassidy MR, Rosenkranz P, McCabe K, Rosen JE, McAneny D. I COUGH: reducing postoperative pulmonary complications with a multidisciplinary patient care program. JAMA Surg. 2013;148(8):740–5. Epub 2013/06/07. doi: 10.1001/jamasurg.2013.358 . 23740240

[23] do Nascimento Junior P, Modolo NS, Andrade S, Guimaraes MM, Braz LG, El Dib R. Incentive spirometry for prevention of postoperative pulmonary complications in upper abdominal surgery. The Cochrane database of systematic reviews. 2014;(2):CD006058. doi: 10.1002/14651858.CD006058.pub3 . 24510642 PMC6769174

[24] Ljungqvist O, Scott M, Fearon KC. Enhanced Recovery After Surgery: A Review. JAMA Surg. 2017;152(3):292–8. Epub 2017/01/18. doi: 10.1001/jamasurg.2016.4952 . 28097305

[25] Alawadi ZM, Leal I, Phatak UR, Flores-Gonzalez JR, Holihan JL, Karanjawala BE, et al. Facilitators and barriers of implementing enhanced recovery in colorectal surgery at a safety net hospital: A provider and patient perspective. Surgery. 2016;159(3):700–12. Epub 20151002. doi: 10.1016/j.surg.2015.08.025 . 26435444

[26] Fiore JF Jr., Castelino T, Pecorelli N, Niculiseanu P, Balvardi S, Hershorn O, et al. Ensuring Early Mobilization Within an Enhanced Recovery Program for Colorectal Surgery: A Randomized Controlled Trial. Ann Surg. 2017;266(2):223–31. Epub 2016/12/21. doi: 10.1097/SLA.0000000000002114 . 27997472

[27] Chan AW, Tetzlaff JM, Altman DG, Laupacis A, Gotzsche PC, Krleza-Jeric K, et al. SPIRIT 2013 statement: defining standard protocol items for clinical trials. Ann Intern Med. 2013;158(3):200–7. doi: 10.7326/0003-4819-158-3-201302050-00583 ; PubMed Central PMCID: PMC5114123. 23295957 PMC5114123

[28] Butcher NJ, Monsour A, Mew EJ, Chan AW, Moher D, Mayo-Wilson E, et al. Guidelines for Reporting Outcomes in Trial Protocols: The SPIRIT-Outcomes 2022 Extension. JAMA. 2022;328(23):2345–56. doi: 10.1001/jama.2022.21243 . 36512367

[29] Castelino T, Fiore JF Jr, Niculiseanu P, Landry T, Augustin B, Feldman LS. The effect of early mobilization protocols on postoperative outcomes following abdominal and thoracic surgery: A systematic review. Surgery. 2016;159(4):991–1003. Epub 20160121. doi: 10.1016/j.surg.2015.11.029 . 26804821

[30] Brull SJ, Kopman AF. Current Status of Neuromuscular Reversal and Monitoring: Challenges and Opportunities. Anesthesiology. 2017;126(1):173–90. Epub 2016/11/08. doi: 10.1097/ALN.0000000000001409 . 27820709

[31] Pasquina P, Tramér MR, Granier J-M, Walder B. Respiratory Physiotherapy To Prevent Pulmonary Complications After Abdominal Surgery. Chest. 2006;130(6):1887–99. doi: 10.1378/chest.130.6.1887 17167013

[32] Kroenke K, Lawrence VA, Theroux JF, Tuley MR. Operative risk in patients with severe obtructive pulmonary disease. Archives of internal medicine. 1992;152(5):967–71. 1580723

[33] Hulzebos EH, Helders PJ, Favie NJ, De Bie RA, Brutel de la Riviere A, Van Meeteren NL. Preoperative intensive inspiratory muscle training to prevent postoperative pulmonary complications in high-risk patients undergoing CABG surgery: a randomized clinical trial. JAMA. 2006;296(15):1851–7. Epub 2006/10/19. doi: 10.1001/jama.296.15.1851 . 17047215

[34] Fernandez-Bustamante A, Frendl G, Sprung J, Kor DJ, Subramaniam B, Martinez Ruiz R, et al. Postoperative Pulmonary Complications, Early Mortality, and Hospital Stay Following Noncardiothoracic Surgery: A Multicenter Study by the Perioperative Research Network Investigators. JAMA Surg. 2017;152(2):157–66. doi: 10.1001/jamasurg.2016.4065 ; PubMed Central PMCID: PMC5334462. 27829093 PMC5334462

[35] Rackley CR, Levitt JE, Zhuo H, Matthay MA, Calfee CS. Clinical evidence of early acute lung injury often precedes the diagnosis of ALI. J Intensive Care Med. 2013;28(4):241–6. Epub 20120624. doi: 10.1177/0885066612450850 ; PubMed Central PMCID: PMC3482421. 22733725 PMC3482421

[36] Mazo V, Sabate S, Canet J, Gallart L, de Abreu MG, Belda J, et al. Prospective external validation of a predictive score for postoperative pulmonary complications. Anesthesiology. 2014;121(2):219–31. Epub 2014/06/06. doi: 10.1097/ALN.0000000000000334 . 24901240

[37] McAlister FA, Bertsch K, Man J, Bradley J, Jacka M. Incidence of and risk factors for pulmonary complications after nonthoracic surgery. Am J Respir Crit Care Med. 2005;171(5):514–7. Epub 20041124. doi: 10.1164/rccm.200408-1069OC . 15563632

[38] Fernandez-Bustamante A, Klawitter J, Repine JE, Agazio A, Janocha AJ, Shah C, et al. Early effect of tidal volume on lung injury biomarkers in surgical patients with healthy lungs. Anesthesiology. 2014;121(3):469–81. Epub 2014/05/09. doi: 10.1097/ALN.0000000000000301 ; PubMed Central PMCID: PMC4165799. 24809976 PMC4165799

[39] Serpa Neto A, Campos PP, Hemmes SN, Bos LD, Bluth T, Ferner M, et al. Kinetics of plasma biomarkers of inflammation and lung injury in surgical patients with or without postoperative pulmonary complications. European journal of anaesthesiology. 2017;34(4):229–38. doi: 10.1097/EJA.0000000000000614 . 28187051 PMC6696995

[40] Parsons PE, Eisner MD, Thompson BT, Matthay MA, Ancukiewicz M, Bernard GR, et al. Lower tidal volume ventilation and plasma cytokine markers of inflammation in patients with acute lung injury. Crit Care Med. 2005;33(1):1–6; discussion 230–2. Epub 2005/01/13. doi: 10.1097/01.ccm.0000149854.61192.dc [pii]. . 15644641

[41] Orkin AM, Gill PJ, Ghersi D, Campbell L, Sugarman J, Emsley R, et al. Guidelines for Reporting Trial Protocols and Completed Trials Modified Due to the COVID-19 Pandemic and Other Extenuating Circumstances: The CONSERVE 2021 Statement. JAMA. 2021;326(3):257–65. doi: 10.1001/jama.2021.9941 . 34152382

[42] Fontanarosa P, Bauchner H, Flanagin A. Authorship and Team Science. JAMA. 2017;318(24):2433–7. doi: 10.1001/jama.2017.19341 . 29279909

