# Supplementary material for: An anesthesia-centered bundle to reduce postoperative pulmonary complications: The PRIME-AIR study protocol
Source: PLoS One. 2023 Apr 6;18(4):e0283748. doi: 10.1371/journal.pone.0283748 (PMC10079125; doi:10.1371/journal.pone.0283748)
Supplement: S1 Checklist — (DOCX) [file pone.0283748.s001.docx]

**SPIRIT checklist** (combined SPIRIT 2013 and SPIRIT-Outcomes 2022 items)

| **Section** | **Item No.** | **SPIRIT 2013 Item** | **SPIRIT-Outcomes 2022 item** | **Location Reported** |
| --- | --- | --- | --- | --- |
| **Administrative information** | | | | |
| Title | 1 | Descriptive title identifying the study design, population,  interventions, and, if applicable, trial acronym | - | 1 |
| Trial registration | 2a | Trial identifier and registry name. If not yet registered, name of  intended registry | - | 4 |
|  | 2b | All items from the World Health Organisation Trial Registration  Data Set | - | 3-4, S1 |
| Protocol version | 3 | Date and version identifier | - | S1 |
| Funding | 4 | Sources and types of financial, material, and other support | - | 1, 20 |
| Roles and responsibilities | 5a | Names, affiliations, and roles of protocol contributors | - | 1 |
|  | 5b | Name and contact information for the trial sponsor | - | 2 |
|  | 5c | Role of study sponsor and funders, if any, in study design; collection, management, analysis, and interpretation of data; writing of the report; and the decision to submit the report for publication, including whether they will have  ultimate authority over any of these activities | - | 2 |
|  | 5d | Composition, roles, and responsibilities of the coordinating center, steering committee, endpoint adjudication committee, data management team, and other individuals or groups overseeing the trial, if applicable  (see Item 21a for data monitoring committee) | - | 16-17, 21 |
| **Introduction** | | | | |
| Background and rationale | 6a | Description of research question and justification for undertaking the trial, including summary of relevant studies (published and unpublished) examining benefits  and harms for each intervention | - | 5-6 |
|  | 6b | Explanation for choice of comparators | - | 6-7 |
| Objectives | 7 | Specific objectives or hypotheses | - | Click to enter text |

| **Section** | **Item No.** | **SPIRIT 2013 Item** | **SPIRIT-Outcomes 2022 item** | **Location Reportedb** |
| --- | --- | --- | --- | --- |
| Trial design | 8 | Description of trial design including type of trial (eg, parallel group, crossover, factorial, single group), allocation ratio, and framework (eg, superiority, equivalence, noninferiority,  exploratory) | - | 8 |
| **Methods: participants, interventions, and outcomes** | | | | |
| Study setting | 9 | Description of study settings (eg, community clinic, academic hospital) and list of countries where data will be collected.  Reference to where list of study sites can be obtained | - | 8, 22 |
| Eligibility criteria | 10 | Inclusion and exclusion criteria for participants. If applicable, eligibility criteria for study centers and individuals who will perform  the interventions (eg, surgeons, psychotherapists) | - | 8, S1 |
| Interventions | 11a | Interventions for each group with sufficient detail to allow replication, including how and when they will be administered  (for specific guidance see TIDieR checklist and guide) | - | 8-12, Figure 1, S1 |
|  | 11b | Criteria for discontinuing or modifying allocated interventions for a given trial participant (eg, drug dose change in response to harms, participant request, or  improving/worsening disease) | - | N/A |
|  | 11c | Strategies to improve adherence to intervention protocols, and any procedures for monitoring adherence (eg, drug tablet return,  laboratory tests) | - | 9-11, S1 |
|  | 11d | Relevant concomitant care and interventions that are permitted or prohibited during the trial | - | 9-11, S1 |
| Outcomes | 12 | Primary, secondary, and other outcomes, including the specific measurement variable (eg, systolic blood pressure), analysis metric (eg, change from baseline, final value, time to event), method of aggregation (eg, median, proportion), and time point for each outcome. Explanation of the clinical relevance of chosen  efficacy and harm outcomes is strongly recommended | - | 12-13 |

| **Section** | **Item No.** | **SPIRIT 2013 Item** | **SPIRIT-Outcomes 2022 item** | **Location Reportedb** |
| --- | --- | --- | --- | --- |
|  | 12.1 |  | Provide a rationale for the selection of the domain for  the trial’s primary outcome | 12 |
|  | 12.2 |  | If the analysis metric for the primary outcome represents within-participant change, define and justify the  minimal important change in individuals | N/A |
|  | 12.3 |  | If the outcome data collected are continuous, but will be analysed as categorical (method of  aggregation), specify the cutoff values to be used | N/A |
|  | 12.4 |  | If outcome assessments will be performed at several time points after randomization, state the  time points that will be used for the analysis | 12, S1 |
|  | 12.5 |  | If a composite outcome is used, define all individual components of the composite outcome | 12, Table 4, S1 |
| Participant timeline | 13 | Time schedule of enrolment, interventions (including any run- ins and washouts), assessments, and visits for participants. A  schematic diagram is highly recommended (see Figure) | - | 13, Figure 1 |
| Sample size | 14 | Estimated number of participants needed to achieve study objectives and how it was determined, including clinical and statistical assumptions supporting  any sample size calculations | - | 13-14 |
|  | 14.1 |  | Define and justify the target difference between treatment groups (eg, the  minimal important difference) | 14 |
| Recruitment | 15 | Strategies for achieving adequate participant enrollment to reach target sample size | - | 14 |
| **Methods: assignment of interventions (for controlled trials)** | | | | |

| **Section** | **Item No.** | **SPIRIT 2013 Item** | **SPIRIT-Outcomes 2022 item** | **Location Reportedb** |
| --- | --- | --- | --- | --- |
| Allocation: Sequence generation | 16a | Method of generating the allocation sequence (eg, computer-generated random numbers), and list of any factors for stratification. To reduce predictability of a random sequence, details of any planned restriction (eg, blocking) should be provided in a separate document that is unavailable to  those who enroll participants or assign interventions | - | 15, S1 |
| Allocation concealment mechanism | 16b | Mechanism of implementing the allocation sequence (eg, central telephone; sequentially numbered, opaque, sealed envelopes), describing any steps  to conceal the sequence until interventions are assigned | - | 15, S1 |
| Implementation | 16c | Who will generate the allocation sequence, who will enroll participants, and who will assign participants to interventions | - | 15, S1 |
| Blinding (masking) | 17a | Who will be blinded after assignment to interventions (eg, trial participants, care providers,  outcome assessors, data analysts), and how | - | 15, S1 |
|  | 17b | If blinded, circumstances under which unblinding is permissible, and procedure for revealing a participant’s allocated intervention  during the trial | - | N/A |
| **Methods: data collection, management, and analysis** | | | |  |
| Data collection methods | 18a | Plans for assessment and collection of outcome, baseline, and other trial data, including any related processes to promote data quality (eg, duplicate measurements, training of assessors) and a description of study instruments (eg, questionnaires, laboratory tests) along with their reliability and validity, if known. Reference to where data collection forms can  be found, if not in the protocol | - | 15-16, S1 |

| **Section** | **Item No.** | **SPIRIT 2013 Item** | **SPIRIT-Outcomes 2022 item** | **Location Reportedb** |
| --- | --- | --- | --- | --- |
|  | 18a.1 |  | Describe what is known about the responsiveness of the study instruments in a population similar to the  study sample | 12 |
|  | 18a.2 |  | Describe who will assess the primary outcome (eg, nurse, parent) | 12 |
|  | 18b | Plans to promote participant retention and complete follow-up, including list of any outcome data to be collected for participants who discontinue or deviate from  intervention protocols | - | 9-11, 15 |
| Data management | 19 | Plans for data entry, coding, security, and storage, including any related processes to promote data quality (eg, double data entry; range checks for data values). Reference to where details of data management  procedures can be found, if not in the protocol | - | 16 |
| Statistical methods | 20a | Statistical methods for analyzing primary and secondary outcomes. Reference to where other details  of the statistical analysis plan can be found, if not in the protocol | - | 16, S1 |
|  | 20a.1 |  | Describe any planned methods to account for multiplicity in the analysis or interpretation of the primary and secondary outcomes (eg, coprimary outcomes, same outcome assessed at multiple time points, or subgroup analyses of an  outcome) | S1 |
|  | 20b | Methods for any additional analyses (eg, subgroup and adjusted analyses) | - | 16, 22, 25 |
|  | 20c | Definition of analysis population relating to protocol non- adherence (eg, as randomized analysis), and any statistical  methods to handle missing data (eg, multiple imputation) | - | 15-16 |
| **Methods: monitoring** | | | |  |
| Data monitoring | 21a | Composition of data monitoring committee (DMC); summary of its | - | 16-17 |

| **Section** | **Item No.** | **SPIRIT 2013 Item** | **SPIRIT-Outcomes 2022 item** | **Location Reportedb** |
| --- | --- | --- | --- | --- |
|  |  | role and reporting structure; statement of whether it is independent from the sponsor and competing interests; and reference to where further details about its charter can be found, if not in the protocol. Alternatively, an explanation of why a DMC is  not needed |  |  |
|  | 21b | Description of any interim analyses and stopping guidelines, including who will have access to these interim results and make the final decision to terminate the  trial | - | 17 |
| Harms | 22 | Plans for collecting, assessing, reporting, and managing solicited and spontaneously reported adverse events and other  unintended effects of trial interventions or trial conduct | - | 16-17 |
| Auditing | 23 | Frequency and procedures for auditing trial conduct, if any, and whether the process will be independent from investigators  and the sponsor | - | 16-18 |
| **Ethics and dissemination** | | | | |
| Research ethics approval | 24 | Plans for seeking research ethics  committee/institutional review board (REC/IRB) approval | - | 18 |
| Protocol amendments | 25 | Plans for communicating important protocol modifications (eg, changes to eligibility criteria, outcomes, analyses) to relevant parties (eg, investigators,  REC/IRBs, trial participants, trial registries, journals, regulators) | - | 18-19 |
| Consent or assent | 26a | Who will obtain informed consent or assent from potential trial participants or authorised surrogates, and how (see Item  32) | - | 19-20 |
|  | 26b | Additional consent provisions for collection and use of participant data and biological specimens in  ancillary studies, if applicable | - | 19-20, 21-22 |
| Confidentiality | 27 | How personal information about potential and enrolled participants will be collected, shared, and maintained in order to protect confidentiality before, during, and  after the trial | - | 20 |

| **Section** | **Item No.** | **SPIRIT 2013 Item** | **SPIRIT-Outcomes 2022 item** | **Location Reportedb** |
| --- | --- | --- | --- | --- |
| Declaration of interests | 28 | Financial and other competing interests for principal investigators for the overall trial and each study  site | - | 20-21 |
| Access to data | 29 | Statement of who will have access to the final trial data set, and disclosure of contractual agreements that limit such access  for investigators | - | 21-22 |
| Ancillary and care after the trial | 30 | Provisions, if any, for ancillary and post-trial care, and for compensation to those who suffer  harm from trial participation | - | 22 |
| Dissemination policy | 31a | Plans for investigators and sponsor to communicate trial results to participants, health care professionals, the public, and other relevant groups (eg, via publication, reporting in results databases, or other data sharing  arrangements), including any publication restrictions | - | 22-23 |
|  | 31b | Authorship eligibility guidelines and any intended use of  professional writers | - | 22 |
|  | 31c | Plans, if any, for granting public access to the full protocol, participant-level data set, and  statistical code | - | 23 |
| **Appendices** | | | | |
| Informed consent materials | 32 | Model consent form and other elated documentation given to  participants and authorised surrogates | - | S1 |
| Biological specimens | 33 | Plans for collection, laboratory evaluation, and storage of biological specimens for genetic or molecular analysis in the  current trial and for future use in ancillary studies, if applicable | - | 11-12, 13, S1 |
