# Supplementary material for: An anesthesia-centered bundle to reduce postoperative pulmonary complications: The PRIME-AIR study protocol
Source: PLoS One. 2023 Apr 6;18(4):e0283748. doi: 10.1371/journal.pone.0283748 (PMC10079125; doi:10.1371/journal.pone.0283748)
Supplement: S1 Appendix — (PDF) [file pone.0283748.s002.pdf]

<u>Supplemental Material</u> to manuscript titled "An Anesthesia-Centered Bundle to Reduce Postoperative Pulmonary Complications: The PRIME-AIR study protocol"

Authors: Ana Fernandez-Bustamante, M.D., Ph.D.[1], Robert A. Parker, Sc.D.[2], Juraj Sprung, M.D., Ph.D.[3], Matthias Eikermann, M.D., Ph.D.[4], Marcelo Gama de Abreu, M.D., M.Sc., Ph.D., D.E.S.A.[5, 6], Carlos Ferrando, M.D., Ph.D., D.E.S.A., M.Sc. [7, 8], B. Taylor Thompson, M.D., Ph.D.[9], Marcos Francisco Vidal Melo, M.D., Ph.D.[10].

Affiliations: [1] Department of Anesthesiology, University of Colorado School of Medicine, Aurora, CO (USA); [2] Biostatistics Center, Massachusetts General Hospital, Department of Medicine, Harvard Medical School, Boston, MA (USA); [3] Department of Anesthesiology and Perioperative Medicine, Mayo Clinic, Rochester, MN, (USA); [4] Department of Anesthesiology, Montefiore Medical Center, Albert Einstein College of Medicine, Bronx, NY, (USA); [5] Department of Intensive Care and Resuscitation, Anesthesiology Institute, Cleveland Clinic, Cleveland, OH, (USA); [6] Department of Outcomes Research, Anesthesiology Institute, Cleveland Clinic, Cleveland, OH; [7] Department of Anesthesiology and Intensive Care, Hospital Clínic Institut D'investigació August Pi i Sunyer, Barcelona (Spain); [8] CIBER de Enfermedades Respiratorias, Instituto de Salud Carlos III, Madrid (Spain); [9] Division of Pulmonary and Critical Care, Department of Medicine, Massachusetts General Hospital, Harvard Medical School, Boston, MA (USA); [10] Department of Anesthesiology, Columbia University Irving Medical Center, New York, NY (USA).

Index:

- Approved **IRB Protocol** by Mass General Brigham IRB, December 16th, 2022 – P.3
- **Informed Consent** template, version January 23rd, 2020 – P.68

IRB PROTOCOL

PRIME-AIR study

I. BACKGROUND AND SIGNIFICANCE

Postoperative pulmonary complications (PPCs) are a major cause of morbidity and mortality for patients undergoing the estimated 51 million annual inpatient surgeries in the US[1–3]. National estimates suggest 1,062,000 PPCs per year, with 46,200 additional deaths, and 4.8 million additional days of hospitalization[2]. Abdominal surgery is associated with the largest absolute number of PPCs[4]. Our long-term goal is to develop and implement perioperative strategies to eliminate PPCs.

Whereas PPCs are as significant and lethal as cardiac surgical complications[1,5], research in the field has received much less attention, and strategies to minimize them are limited. Indeed, the recommendations of the American College of Physicians to mitigate PPCs highlight the lack of evidence guiding current intraoperative anesthetic practice and perioperative care[5,6]. In contrast, we and others have evidence to suggest a crucial *role of perioperative interventions such as ventilatory strategies*[7–10]*, and administration and reversal of neuromuscular blocking agents*[11,12] *in reducing PPCs,* consistent with the beneficial effects of lung protective ventilation in the acute respiratory distress syndrome (ARDS)[13]. *Surgical patients differ substantially from ARDS patients* as most have no or limited lung injury at the onset of surgery. Yet, during and after abdominal surgery, systemic inflammation due to surgical trauma[14] and translocation of endotoxin[15] and bacteria[16] predispose to indirect lung injury; while resorptive and compression atelectasis from limited diaphragmatic excursion, surgical retractors, muscle paralysis, high inspired oxygen fractions, prolonged supine position, and pain[17–19] predispose to direct lung injury[20]. The ensuing multiple-hit facilitates the onset of clinical or sub-clinical lung injury that places patients at risk for PPCs[19,21,22] .

Recent major trials on PPCs focused on mechanical ventilation. Futier *et al*.[7] demonstrated the benefit of intraoperative lung recruitment and low stretch on a composite of pulmonary and extrapulmonary outcomes. Hemmes *et al*.[23] found no effect of intraoperative constant high *vs*. low positive end-expiratory pressure (PEEP) on PPCs. While the first trial highlighted the relevance of ventilatory strategies to postoperative outcomes, the second led to the controversial recommendation that low PEEP should be the standard of care in abdominal surgery[23,24] , a practice anticipated to worsen lung collapse. No trial addressed the effects of neuromuscular blocking agents on PPCs. This evidence gap and the resulting wide and unexplained variability in current anesthetic practices is a major public health issue as some practices within usual care appear to be suboptimal and even potentially injurious[8,11,12 25].

Accordingly, solid evidence to guide optimal perioperative anesthetic care to prevent PPCs is greatly needed.

We propose to conduct a large prospective multicenter randomized controlled pragmatic trial with a blinded assessor to compare PPCs in patients undergoing major open abdominal surgery receiving an individualized management bundle *versus* usual care. ***We hypothesize*** *that this anesthesia-centered bundle, based on our recent findings and focused on perioperative lung protection, will minimize multiple and synergistic factors responsible for the multiple-hit perioperative pulmonary dysfunction and result in decreased incidence and severity of PPCs*.

II. SPECIFIC AIMS

We will leverage the experience achieved in a successful pilot collaboration among US academic centers[26] to study this hypothesis by pursuing the following aims:

Specific Aim 1: To compare the number and severity of PPCs in patients receiving an individualized perioperative anesthesia-centered bundle to those in patients receiving usual anesthetic care during open abdominal surgery. The anesthesia-centered bundle will consist of: (a) mechanical ventilation utilizing individualized PEEP with recruitment maneuvers to maximize respiratory system compliance and minimize driving pressures; (b) individualized use of neuromuscular blocking agents and their reversal; and (c) postoperative lung expansion to maintain lung recruitment following surgery. We will use the patient classification of PPC severity during the first 7 days after surgery as the primary outcome in order to specifically assess the effect of our intervention not only on PPC incidence but also on severity distribution.

Specific Aim 2: To assess the effect of the proposed bundle on plasma levels of lung injury biomarkers. We hypothesize that our intervention will minimize lung mechanical injury and systemic inflammation by reducing overinflation and atelectasis. Thus, we anticipate that plasma levels of biomarkers of inflammation, epithelial, and endothelial cell injury will be lower in the intervention arm as previously described with lung protective ventilation for ARDS[27,28], and in our pilot[29] and others[30] data for PPCs. The mechanistic insights from this aim will facilitate bundle dissemination and support adoption as it has for lung protective ventilation for ARDS.[28,31,32,] In the process, we will create a novel biobank uniquely focused on PPCs, and gain mechanistic insights on early perioperative lung protection.

At the end of this project, we expect that this novel and clinically feasible anesthesiacentered bundle will change clinical practice and reduce pulmonary morbidity in the large population of patients undergoing open abdominal surgery.

III. SUBJECT SELECTION

This is a multicenter study including 17 US academic medical centers. The Massachusetts General Hospital (MGH) will receive and analyze the de-identified data; the University of Colorado School of Medicine will receive and analyze all blood samples; and Columbia University will function as the clinical coordinating center.

We will target adult patients of both genders (N =approximately 790 randomized to obtain 750 fully studied participants)
undergoing open abdominal surgery and classified as intermediate or high risk for PPCs using the well-established and validated ARISCAT risk score for PPCs (Table 1)[4].

**Table 1. ARISCAT risk score for PPCs[4]**	
**Variable**	**Score**
Age (yr)	
≤ 50	0
51 – 80	3
> 80	16
Preoperative S_pO_2, %	
≥ 96	0
91 – 95	8
≤ 90	24
Respiratory infection in the last month	17
Preoperative anemia (≤ 10 g/dL)	11
Surgical incision	
Peripheral	0
Upper abdominal	15
Intrathoracic	24
Duration of surgery, h ≤ 2	0
> 2 to 3	16
> 3	23

Inclusion criteria: Participants must meet all of the following inclusion criteria to participate in this study:

- Adults (≥18 years) scheduled for elective surgery with expected duration ≥2 hours
- Open abdominal surgery including: gastric, biliary, pancreatic, hepatic, major bowel, ovarian, renal tract, bladder, prostatic, radical hysterectomy, and pelvic exenteration
- Intermediate or high risk of PPCs defined by an ARISCAT risk score≥26

Exclusion criteria: Candidates meeting any of the exclusion criteria at baseline will be excluded from study participation. These criteria are:

- Inability or refusal to provide consent
- Inability or significant difficulty to perform any study interventions, including incentive spirometry, ambulation and/or maintaining follow-up contact with study personnel for up to 90 days after the date of surgery.
- Participation in any interventional research study within 30 days of the time of the study.
- Previous surgery within 30 days prior to this study.
- Pregnancy
- Emergency surgery
- Severe obesity (above Class I, BMI≥35 kg/m^2)
- Significant lung disease: any diagnosed or treated respiratory condition that (a) requires home oxygen therapy or non-invasive ventilation (except nocturnal treatment of sleep apnea without supplemental oxygen), (b) severely limits exercise tolerance to <4 METs (e.g., patients unable to do light housework, walk flat at 4 miles/h or climb one flight of stairs), (c) required previous lung surgery, or (d) includes presence of severe pulmonary emphysema or bullae
- Significant heart disease: cardiac conditions that limit exercise tolerance to <4 METs
- Renal failure: peritoneal or hemodialysis requirement or preoperative creatinine ≥2 mg/dL

- Neuromuscular disease that impairs ability to ventilate without assistance
- Severe chronic liver disease (Child-Turcotte-Pugh Score >9, Appendix I)
- Sepsis
- Malignancy or other irreversible condition for which 6-month mortality is estimated ≥20%
- Bone marrow transplant

IV. SUBJECT ENROLLMENT

Identification, screening and consent: Participant identification and screening will be done in all sites from pre-admission/anesthesia clinic schedules (manual or electronic), surgeons' office schedules, operating room schedules and by communication with the relevant nursing and medical staff. Patient advocacy groups are not currently engaged because this study does not focus on a patient population with a known disease. Instead, our study population is defined by patients with pre-defined risk level for postoperative pulmonary complications that are scheduled to undergo abdominal surgery. Thus, the anesthesiologists and surgeons performing these procedures and their offices' personnel are the clinical communities critical for successful recruitment and retention for this study. We have shown successful recruitment in similar patients for the pilot R34 study, with no objection by the health care providers participating in the patients' clinical care in having any patient enrolled in the study.

Once subjects are identified as meeting inclusion criteria, they will be contacted according to each site's procedures including approaches such as in person during hospital clinic visits or videoconference through use of a site-specific approved HIPAAcompliant telemedicine videoconferencing platform. Besides regular signature of the paper consent form, we will also offer the option for electronic consent signature (eConsent).

Massachusetts General Hospital (MGH): Once subjects are identified as meeting inclusion criteria, the study will be first introduced to them by a member of the treatment team known to the subject. Such introduction will be done through one of the following manners: in person, teleconference, phone, IRB approved letter, secure email, reproducing the IRB approved letter, distributed flier, or Patient Gateway.

Provided patients express agreement to be contacted, they will be further approached through one of the following methods: (i) in person during a clinic visit, (ii) by HIPAA compliant videoconference, or (iii) by phone contact (a likely case at the MGH as several patients are referred from and assessed in other hospitals and come to the MGH only on the day of surgery specifically to undergo the procedure). Direct contact will be sought for patients who answered positively to the Research Opportunities Direct to You (RODY) program question. If contact occurs in person during a clinic visit, consent will be directly obtained after all questions and concerns are addressed and answered. If subjects prefer, they will be allowed time to consult with their family or personal doctors, and consent will only be obtained after patients are comfortable with

their decision. For patients contacted by mail or phone, once initial contact has been made with the subject and the study explained, we will ask permission either to send the consent form to the subject via mail or email for their review or to provide them with eConsent instructions. If the subject agrees, we will mail or email the consent or provide the eConsent instructions and we will follow up with a telephone call to discuss any questions or concerns the subject may have. All questions and concerns will be addressed during the follow up call. All subjects will be given the opportunity to speak with a doctor during the consent process either in person or through videoconference or phone. If the subject then agrees to participate, consent will be obtained in person on the day of their surgery or through eConsent utilizing the MGH approved system. Pregnant women and children are not being enrolled in this study. No class of subjects, including institutionalized individuals, minorities, or other potentially vulnerable populations, will be excluded or preferentially targeted. Documentation of reasons for ineligibility and for non-participation of eligible candidates will be performed with a screening log.

The subset of patients only physically present in the hospital and able to ultimately sign the consent in the day of surgery presents a practical issue as per protocol the anesthesia team caring for the patient is assigned according to the group the patient is randomized to and the assignment of anesthesiologists to cases is done in the day before surgery. To address this, patients in that subset expressing preliminary agreement with their participation and not able to sign an eConsent will be prerandomized. If assigned to the intervention group, their anesthesia team will be assigned accordingly. They will also receive by email the study's brochure and a link to a YouTube explanatory video (provided below). Such material provides brief information on postoperative pulmonary complications and methods to reduce them. Consequently, it will increase patients' education on their perioperative care. In fact, similar types of materials are commonly used in other institutions for routine clinical care. Randomization will be confirmed, and patients considered enrolled in the study only when they ultimately sign the informed consent before surgery.

Brigham and Women's Hospital (BWH): Participant identification and screening will be done at BWH from pre-admission/anesthesia clinic schedules (manual or electronic), surgeons' office schedules, operating room schedules and by communication with the relevant nursing and medical staff. Patient advocacy groups are not currently engaged because this study does not focus on a patient population with a known disease. Instead, our study population is defined by patients with pre-defined risk level for postoperative pulmonary complications that are scheduled to undergo abdominal surgery. Thus, the anesthesiologists and surgeons performing these procedures and their offices' personnel are the clinical communities critical for successful recruitment and retention for this study. We have shown successful recruitment for our past interventional trials, with no objection by the health care providers participating in the patients' clinical care in having any patient enrolled in the study.

Once subjects are identified as meeting our inclusion criteria, they will be contacted in person during their hospital clinic visit or videoconference visit (preferably in the

preoperative anesthesia test center (PATC) at BWH. Pregnant women and children are not being enrolled in this study. No class of subjects, including institutionalized individuals, minorities, or other potentially vulnerable populations, will be excluded or preferentially targeted. Signed informed consent for participating in the study will be obtained prior to randomization and performance of any study procedures either inperson or through eConsent utilizing the BWH approved system. Documentation of reasons for ineligibility and for non-participation of eligible candidates will be performed with a screening log.

Individuals responsible for screening, and approaching potential participants will be members of the research team: attending physicians, certified or in-training anesthesiologists, and research nurses, coordinators and assistants. All will have appropriate human subjects research training per institutional requirements (e.g., CITI training or equivalent). The sites' research coordinators and assistants are experienced in screening and approaching participants and will be closely monitored by the BWH site-PI (Dr. Frendl).

University of Colorado: Participant identification and screening will be done at University of Colorado Hospital from pre-admission/anesthesia clinic schedules (manual or electronic), surgeons' office schedules, operating room schedules and by communication with the relevant nursing and medical staff. Patient advocacy groups are not currently engaged because this study does not focus on a patient population with a known disease. Instead, our study population is defined by patients with pre-defined risk level for postoperative pulmonary complications that are scheduled to undergo abdominal surgery. Thus, the anesthesiologists and surgeons performing these procedures and their offices' personnel are the clinical communities critical for successful recruitment and retention for this study. We have shown successful recruitment for our past interventional trials, with no objection by the health care providers participating in the patients' clinical care in having any patient enrolled in the study.

Once subjects are identified as meeting our inclusion criteria, they will be contacted in person during their hospital clinic visit or videoconference visit (preferably in the preoperative center at University of Colorado Hospital). Pregnant women and children are not being enrolled in this study. No class of subjects, including institutionalized individuals, minorities, or other potentially vulnerable populations, will be excluded or preferentially targeted. Signed informed consent for participating in the study will be obtained prior to randomization and performance of any study procedures either inperson or through eConsent utilizing the University of Colorado approved system. Documentation of reasons for ineligibility and for non-participation of eligible candidates will be performed with a screening log.

Individuals responsible for screening, and approaching potential participants will be members of the research team: attending physicians, certified or in-training anesthesiologists, and research nurses, coordinators and assistants. All will have appropriate human subjects research training per institutional requirements (e.g., CITI training or equivalent). The sites' research coordinators and assistants are experienced

in screening and approaching participants and will be closely monitored by the University of Colorado site-PI (Dr. Fernandez-Bustamante).

Duke University Health System: After obtaining permission from the operating surgeon, surgical subjects will be screened for eligibility by the study coordinator by reviewing the surgical schedule. If the patient meets eligibility criteria, the study team member will first check the Duke office of Clinical Research (DOCR) Opt Out list, to ensure that the patient has not opted out of research. If it is shown that the patient has not opted out of research, then they will be contacted by the research coordinator and will discuss their willingness to participate in the research protocol either over the phone or in person at a clinic visit or videoconference visit. An IRB approved phone script will be used during phone communication. If the subject is in agreement, the coordinator will present the research protocol in its entirety to the prospective participant and will answer any and all questions as they arise. If the patient decides during the phone call that they would like to opt out of research, the study team member will direct the patient to contact the Research Navigators to update their status at 919-668-5111. If the patient agrees to participate, the coordinator will ask the subject to sign and date the appropriate consent form at their first visit if being contacted over the phone or they will sign at the time of consent if in person or through eConsent utilizing the Duke University approved system. A copy of this consent form will be given to the subject and a copy of the consent form will be added to the patient's medical record.

Northwestern University/ Northwestern Medicine (NM): Subjects will be identified by the primary care surgeon during a preoperative visit or during their visit to the NM preoperative clinic or videoconference visit. If they have been identified as meeting the inclusion criteria they will be contacted by one of the study team members in person or by videoconference or by telephone. For subjects contacted by telephone, study team members will use a prepared script to introduce the study including study parameters as well as the consent document and the requirements to participate. If the subject requests the consent document it will then be e-Mailed via Northwestern Encrypted eMail for their perusal or through eConsent utilizing the Northwestern University approved system. This will allow additional time for the subject to consult with family and personal physicians. A study team member will conduct a follow up telephone call to address whether the subject has additional questions regarding the study. The study team member will inquire whether the subject would like to participate in the study. On the day of surgery the study team member will meet with the subject in the preoperative area and will review the inclusion and exclusion criteria. The research study team member will also inquire whether the subject has any additional questions regarding the study. The study team members will provide ample time to answer any questions posed during the questioning. After this review the research study team will obtain written informed consent. Pregnant women and children are not being enrolled in this study. No class of subjects, including institutionalized individuals, minorities, or other potentially vulnerable populations, will be excluded or preferentially targeted.

Documentation of reasons for ineligibility and for non-participation of eligible candidates will be performed with a screening log.

Beth Israel Deaconess Medical Center: Subjects will be identified from the perioperative information management system (PIMS), surgical consult lists, cardiac surgical clinic visit schedules and pre-admission testing (PAT) clinic visit schedules. Patients who meet inclusion criteria with no exclusions will be approached for consent either in-person or through videoconference visit. The study will be explained in clear, layperson language. All study procedures will be disclosed. Risks to the patient will be explained; no individual patient benefits will be promised. Patients will be given ample time to make a decision regarding participation. The patient will be given the opportunity to ask questions and confer with family members and others. If the patient elects to consent to the trial, they will sign and date the consent form(s) in the presence of authorized research personnel, who will also sign and date or through eConsent utilizing the BIDMC approved system.

Stanford Hospital and Clinics: Once subjects were identified as meeting our inclusion criteria by Epic search previously to their surgery. They will be contacted in person during a hospital clinic visit (preoperative clinic visit) or by videoconference. Subjects of research during the preop clinic visit will be consented after all questions and concerns were addressed and answered either in-person or through eConsent utilizing the Stanford University approved system. If subjects prefer, they will be allowed time to consult with their family or personal doctors, and consent will only be obtained after patients are comfortable with their decision. Pregnant women and children are not being enrolled in this study. No class of subjects, including institutionalized individuals, minorities, or other potentially vulnerable populations, will be excluded or preferentially targeted. Documentation of reasons for ineligibility and for non-participation of eligible candidates will be performed with a screening log.

Individuals responsible for screening and approaching potential participants will be members of the research team: attending physician and research assistant. All will have appropriate human subjects research training per institutional requirements (e.g., CITI training or equivalent). The sites' research assistant is experienced in screening and approaching participants and will be closely monitored by the SHC site-PI (Dr. Tanaka).

UCSF Medical Center at Mission Bay: Once subjects are identified as meeting our inclusion criteria, they will be contacted either in person during a hospital clinic visit (e.g., to their PCP, surgeon, preoperative clinic visit, provided patients expressed to their primary health care providers agreement to be contacted), by phone or by videoconference or through mail (via IRB approved MD letter and "opt out" post card), or by direct or phone contact. Direct contact will be sought for patients who answered positively to RODY. If contact occurs in person during a clinic visit, consent will be directly obtained after all questions and concerns are addressed and answered. If subjects prefer, they will be allowed time to consult with their family or personal doctors, and consent will only be obtained after patients are comfortable with their decision. For patients contacted by mail and phone, once initial contact has been made with the

subject, we will ask permission to send the consent form to the subject via mail or email or through eConsent utilizing UCSF approved system for their review. If the subject agrees, we will mail or email the consent and follow up with a telephone call to discuss any questions or concerns the subject may have. If emailing the consent form, we will do so by using encryption via the “Send Secure” function. All questions and concerns will be addressed during the follow up call. All subjects will be given the opportunity to speak with a doctor during the consent process. If the subject then agrees to participate, consent will be obtained in person on the day of their surgery. Pregnant women and children are not being enrolled in this study. No class of subjects, including institutionalized individuals, minorities, or other potentially vulnerable populations, will be excluded or preferentially targeted. Documentation of reasons for ineligibility and for non-participation of eligible candidates will be performed with a screening log.

Individuals responsible for screening, and approaching potential participants will be members of the research team: attending physicians, certified or in-training anesthesiologists, and research nurses, coordinators and assistants. All will have appropriate human subjects research training per institutional requirements (e.g., CITI training or equivalent). The sites’ research coordinators and assistants are experienced in screening and approaching participants and will be closely monitored by the UCSF site-PIs (Drs. Jae-Woo Lee or Lee-Lynn Chen).

University of Rochester Medical Center

Participant identification and screening will be done at URMC from preadmission/anesthesia clinic schedules (manual or electronic), surgeons’ office schedules, and by communication with the relevant nursing and medical staff.

Once subjects are identified as meeting inclusion criteria, they will be contacted in person during their pre-operative visit (preferably in the Center for Perioperative Medicine (CPM) or at the surgical clinic visit either in-person or through videoconference. Pregnant women and children are not being enrolled in this study. No class of subjects, including institutionalized individuals, minorities, or other potentially vulnerable populations, will be excluded or preferentially targeted. Signed informed consent for participating in the study will be obtained prior to randomization and performance of any study procedures. A URMC approved eConsent platform can be used.

The potential subject will determine whether they prefer to review the consent in person or prefer the option of an eConsent at a later time.

If potential subject prefers to use eConsent, the study team will request verbal permission to send the link to the URMC REDCap eConsent via email or text. The request will state: “Because URMC can’t control the security of email or text messages once we send them, we need your permission to text or email you. Do you want to receive the link to the eConsent via text or email?” The permission will be documented. The email/text will not include PHI and only includes a link to the URMC REDCap

eConsent. To ensure security, 2 step verification is required to access the link, The study team will verbally communicate to the subject a passcode required for the 2-step verification for the REDCap eConsent process. The subject will receive an email/text with a link to the REDCap eConsent and a verbal passcode the user enters on the website to initiate and sign the eConsent at home on their computer, phone or electronic device.

The subject will use the link to access the Redcap eConsent system. Once the subject completes 2-step verification entering the passcode on the RedCap website, they can then initiate the eConsent process. The study team will coordinate a time to review the eConsent with subject electronically over the phone and answer any questions the subject may have. The subject will then answer comprehension questions to be certain the subject understands the clinical study. (If there is any discrepancy in these answers the research staff will be notified and they will contact the subject via phone to discuss and review the consent and clinical study again.)

Once comprehension questions are answered correctly, the subject can sign the eConsent to acknowledge that they have read and are interested in participating in this clinical study. Subject's signature on the eConsent will be obtained using an electronic signature. Once the consent form is signed and submitted, subjects will be able to receive a print out of the paper copy, download a pdf, and/or receive an email with a PDF attachment of the signed consent form.

Documentation of reasons for ineligibility and for non-participation of eligible candidates will be performed with a screening log.

Individuals responsible for screening, and approaching potential participants will be members of the research team: attending physicians, advanced care providers (NP, PA's), and research nurses and coordinators. All will have appropriate human subjects research training per institutional requirements (e.g., CITI training/GCP).

Mayo Clinic Rochester: Participant identification and screening will be done at Mayo Clinic from pre-admission/anesthesia clinic schedules, surgeons' office schedules, operating room schedules and by communication with the relevant nursing and medical staff. Patient advocacy groups are not currently engaged because this study does not focus on a patient population with a known disease. Instead, our study population is defined by patients with pre-defined risk level for postoperative pulmonary complications that are scheduled to undergo abdominal surgery. Thus, the anesthesiologists and surgeons performing these procedures and their offices' personnel are the clinical communities critical for successful recruitment and retention for this study. We have shown successful recruitment for our past interventional trials, with no objection by the health care providers participating in the patients' clinical care in having any patient enrolled in the study.

Once subjects are identified as meeting our inclusion criteria, they will be contacted in person during their preoperative evaluation (POE) appointment at Mayo Clinic Rochester. Pregnant women and children are not being enrolled in this study. No class of subjects, including institutionalized individuals, minorities, or other potentially

vulnerable populations, will be excluded or preferentially targeted. Signed informed consent for participating in the study will be obtained prior to randomization and performance of any study procedures. Documentation of reasons for ineligibility and for non-participation of eligible candidates will be performed with a screening log.

Individuals responsible for screening and approaching potential participants will be members of the research team: attending physicians, certified or in-training anesthesiologists, and research nurses, coordinators and assistants. All will have appropriate human subjects research training per institutional requirements (e.g., CITI training or equivalent). The sites' research coordinators are experienced in screening and approaching participants and will be closely monitored by the Mayo Clinic Rochester site-PI (Dr. Sprung).

Memorial Sloan Kettering Cancer Center: Participant identification and screening will be done at MSKCC from Pre-Surgical Testing clinic schedules (electronic), operating room schedules and by communication with the relevant nursing and medical staff. Individuals responsible for screening will be members of the research team. The study population will be defined by patients with pre-defined risk level for postoperative pulmonary complications that are scheduled to undergo abdominal surgery. The eligibility and exclusion criteria do not discriminate either explicitly or implicitly against gender, race or ethnicity. Pregnant women and children ≤18 will not be enrolled onto the study.

Once subjects are identified as meeting the inclusion criteria, they will be approached during their Pre-Surgical Testing visit at MSKCC by a member of the research team. Details pertinent to the trial, expected outcomes, potential risks and adverse outcomes will be discussed in detail before enrollment. During the initial conversation between the investigator/research staff and the patients, the patient may be asked to provide certain health information that is necessary to the recruitment and enrollment process. They will use the information provided by the patient and/or medical record to confirm that the patient is eligible.

Signed informed consent for participating in the study will be obtained prior to randomization and performance of any study procedures and documented in the patient's chart. Documentation of reasons for ineligibility and for non-participation of eligible candidates will be performed in a screening log and a note to file that will be sent to the patient's electronic medical record. The research staff will destroy all information collected on the patient during the initial conversation and medical records review, except for any information that must be maintained for the screening log purposes.

University of Massachusetts Medical School- Hospital System: Participant identification and screening will be done from pre-admission/anesthesia clinic schedules (manual or electronic), surgeons' office schedules, operating room schedules and by communication with the relevant nursing and medical staff. Our study population is defined by patients with pre-defined risk level for postoperative pulmonary complications that are scheduled to undergo abdominal surgery. Thus, the anesthesiologists and

surgeons performing these procedures and their offices' personnel are the clinical communities critical for successful recruitment and retention for this study.

Once subjects are identified as meeting our inclusion criteria, a provider will get a verbal consent and a study team member will follow-up with an IRB approved letter and/or telephone call (approved phone script) which will provide a brief introduction of the study to the patient. If interest is expressed, we will meet with the patient during a clinic visit to conduct the informed consent process. Other patients may be approached for the first time in the pre-admission clinic or surgeons' office. Virtual clinical visits and electronic consent may be used as a COVID-19 alternative to consent the patient prior to surgery. Patients may be consented same day with prior interest and study review. All subjects will be given the opportunity to ask the study team questions pertaining to the study during the consent process. A provider will be available to consult or review the study with the patient. If the patient's consent process takes place virtually and the patient would like to speak to a provider, a University of Massachusetts Medical School study team provider will follow-up with the patient via telephone. If the subject then agrees to participate, consent will be obtained in person or via an IRB approved electronic consent (RedCap). Pregnant women and children are not being enrolled in this study. No class of subjects, including institutionalized individuals, minorities, or other potentially vulnerable populations, will be excluded or preferentially targeted. Documentation of reasons for ineligibility and for non-participation of eligible candidates will be performed with a screening log.

Individuals responsible for screening and approaching potential participants will be members of the research team: attending physicians, certified or in-training anesthesiologists, and surgeon, research nurses, coordinators and/or assistants. All will have appropriate human subjects research training per institutional requirements (e.g., CITI training or equivalent).

Columbia University Irving Medical Center: Participant identification and screening will be done at Columbia University Irving Medical Center from pre-admission/anesthesia clinic schedules, surgeons' office schedules, operating room schedules and by communication with the relevant nursing and medical staff. Patient advocacy groups are not currently engaged because this study does not focus on a patient population with a known disease. Instead, our study population is defined by patients with pre-defined risk level for postoperative pulmonary complications that are scheduled to undergo abdominal surgery. Thus, the anesthesiologists and surgeons performing these procedures and their offices' personnel are the clinical communities critical for successful recruitment and retention for this study. We have shown successful recruitment for our past interventional trials, with no objection by the health care providers participating in the patients' clinical care in having any patient enrolled in the study.

Once subjects are identified as meeting our inclusion criteria, they will be contacted during their preoperative evaluation (POE) appointment at Columbia University Irving Medical Center. Pregnant women and children are not being enrolled in this study. No

class of subjects, including institutionalized individuals, minorities, or other potentially vulnerable populations, will be excluded or preferentially targeted. Signed informed consent for participating in the study will be obtained prior to randomization and performance of any study procedures. Documentation of reasons for ineligibility and for non-participation of eligible candidates will be performed with a screening log.

Individuals responsible for screening and approaching potential participants will be members of the research team: attending physicians, certified or in-training anesthesiologists, and research nurses, coordinators and assistants. All will have appropriate human subjects research training per institutional requirements (e.g., CITI training or equivalent). The sites' research coordinators are experienced in screening and approaching participants and will be closely monitored by the Columbia University Irving Medical Center, and the site PI, Dr. Wagener.

Cleveland Clinic Foundation: Subjects will be identified using EMR system EPIC, searching in the pre-OP visit schedule. Only patients that meet the inclusion criteria will be approached in person during the pre-OP visit. Surgeons' agreement was obtained to contact their patients. After presentation of the study protocol to the patient and all questions and concerns are addressed and answered consent will be directly obtained from those patients that agree to participate. All subjects will be given the opportunity to speak with a doctor during the consent process. In the case the patients decide to review the protocol and/or consent form at home or consult with their family or personal doctors, the patients will contact the site-PI with their final decision or he/she will be approached again on day of the surgery to inquire their willingness to participate. If the subject then agrees to participate, consent will be obtained in person either during the pre-OP visit or the day of their surgery. Pregnant women and children are not being enrolled in this study. No class of subjects, including institutionalized individuals, minorities, or other potentially vulnerable populations, will be excluded or preferentially targeted. Documentation of reasons for ineligibility and for non-participation of eligible candidates will be performed with a screening log.

Study coordinators, Research fellows and research assistants will be responsible for screening, and approaching potential participants. All will have appropriate human subjects research training per institutional requirements (e.g., CITI training or equivalent). The sites' research coordinator and assistants are experienced in screening and approaching participants and will be closely monitored by the CCF site-PI (Dr. Marcelo Gamma de Abreu).

University of Nebraska Medical Center: Subject identification and screening will be completed at Nebraska Medicine using surgeons' clinic schedules, pre-anesthesia clinic schedules, operating room schedules and by communication with relevant medical staff. Once subjects are screened and identified as meeting inclusion criteria, the research team will introduce the study by the following means: in person, teleconference, phone, or OneChart patient portal.

Should the potential research candidate express agreement to be contacted, they will be approached by the research team (all of whom are trained per institutional requirements and have CITI training or equivalent) via the following means: in person during a clinic visit, by HIPAA compliant videoconference, or by phone contact. If contact occurs during an in person clinic visit, the potential subject will be allowed time to consult with their family or seek medical consultation. Consent will be obtained only after allowed ample time to develop an informed decision. If contact occurs during a videoconference or by phone, the research study team will then ask permission to send the potential subject the consent form via email or mail for their review. A follow up call will then be made to address questions and concerns, and the site PI will be made available during the entire consent process, either in person or via videoconference/phone. If the potential subject is agreeable to participation, consent will be obtained in person prior to surgery, as eConsent cannot be utilized in the state of Nebraska for this study. No vulnerable populations, including but not limited to minorities, will be preferentially excluded or targeted. Documentation of potential candidate ineligibility or non-participation of eligible candidates will be performed with a screening log kept on a secure server.

There exists a logistical concern for those patients who remotely pre-consent and are only available to be physically present in the hospital on the day of surgery. There also exists the issue of Anesthesia staff at UNMC not being assigned to cases until the day prior to surgery. In these instances, it will be properly documented and written into the IRB application to allow for study staff to pre-randomize the subject prior to obtaining written consent on day of surgery so that randomized anesthesia staff can be properly assigned to the subject's case and if subject might randomize to the intervention arm, the subject would be able to receive educational materials pertaining to the clinical trial prior to the date of surgery. Subject will specifically receive the study brochure and a link to a YouTube explanatory video via email. The verbal consent would be properly documented per IRB requirements and endorsed by a witness. Although randomization may occur prior to a wet signature of the informed consent in this instance, the subject will only be considered enrolled in the study once the informed consent is ultimately signed with a wet signature on the day of surgery.

Most of the research teams involved in this study have successfully implemented previous clinical studies within and outside our network[3,10,33–37].

Recruitment sites and Recruitment rate: We currently have 17 sites for enrollment. To achieve our goal of 750 studied patients within the period of approximately 36 months, an average of 1.5 patients recruited/site/month are required. We have agreed on a capitated enrollment model with no maximum of patients/month per participating center as recommended by the DSMB.

Randomization: Two types of randomization are planned in the study: (1) randomization of individual patients and (2) randomization of attending anesthesiologists to give either the intervention bundle or usual care. Randomization will use a centralized web-based system in the Statistics and Data Coordinating Center (SDCC), with stratification by site.

Use of a centralized service will reduce the potential for selection bias of participants. Randomization of attending anesthesiologists is intended to reduce potential contamination in the control group. Only the group allocated to deliver the intervention group will be given training in the intervention protocol described below.

Once the patient is enrolled they will be randomly assigned to one of the two groups as described below.

V. STUDY PROCEDURES

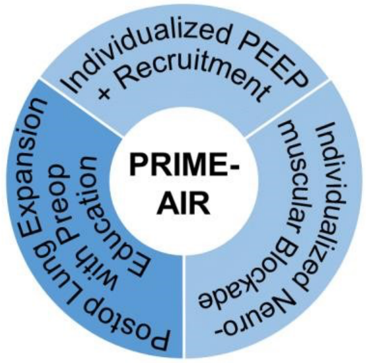

Fig. 1. Intervention categories composing the **PRIME-AIR** bundle to reduce PPCs. Light-blue = intraoperative, dark blue = pre- and postoperative.

PRIME-AIR is a multicenter, prospective, controlled openlabel parallel-group clinical trial to test the effect of an individualized perioperative anesthesia-centered bundle on the number and severity of postoperative pulmonary complications (PPCs). Eligible patients will be adults undergoing major open abdominal surgery with intermediate or high risk for developing PPCs. These represent a large population of abdominal surgery patients sustaining the relevance and generalizability of the study. We plan to study a total of 750 adult patients undergoing abdominal surgery with general anesthesia and mechanical ventilation.

Patients will receive the intervention perioperatively and will be followed by hospital visits (in-person or videoconference) and a daily phone call until discharged home or until 7 days after surgery. Phone calls or hospital visits (in-person or videoconference) will also be done immediately after postoperative days 7, 30 and 90 to determine additional complications.

Strategy and Experimental Design: We hypothesize that an anesthesia-centered bundle, focused on perioperative lung expansion, will minimize the synergistic factors responsible for the multiple-hit perioperative pulmonary dysfunction and injury, and will result in decreased incidence and severity of PPCs.

We will recruit adult patients with moderate or high risk for PPCs scheduled for major open abdominal surgery during 36 months and randomize them 1:1 to either the bundle or usual care. Our anesthesia-centered individualized bundle will consist of pre-, intra- and postoperative interventions aimed at optimizing perioperative lung expansion.

The bundle is summarized in the mnemonic **PRIME-AIR**: **P**EEP (positive end-expiratory pressure), **R**ecruitment, **I**ncentive spirometry, **M**uscle relaxant optimization, preoperative **E**ducation, postoperative early **A**mbulation, **I**ndividualized, and **R**einforced. Specifically, the intervention bundle consists of: (1) individualized PEEP with recruitment maneuvers to maximize respiratory system compliance and minimize driving pressures; (2) protocolized neuromuscular blockade agents administration and reversal based on neuromuscular monitoring; (3) postoperative lung expansion interventions (incentive spirometry and early ambulation) with adherence enhanced by supervision and preoperative education on PPCs and performance of lung expansion maneuvers. This is summarized in Fig. 1.

Overview of Patient Flow in the Trial (Fig. 2): Trial activities for each participant will commence with enrollment. This will involve personal or phone contact of a member of the study team with patients before surgery. After signing informed consent (or, for participants recruited by phone who will be signing the informed consent document on the day of surgery, after agreeing to participate; please, see section IV. Subject Enrollment - MGH), patients will be randomized to receive the intervention (intervention group) or usual perioperative care (control group) using a web-based system in the SDCC. The most intense interventional phase will occur during hospital admission for surgery. Participants will receive three daily follow-up visits (in person or videoconference) by an unblinded investigator until fully ambulatory or until 7 days after surgery (whichever is longer). Recognizing that three visits per day may be difficult for specific participants and clinical situations, we will consider at least 2 visits per day as adequate for the purposes of the study. Besides the visits by the unblinded investigator, a daily phone call, videoconference, or in-person visit will be done by a blinded investigator until discharge or 8 days after surgery. This period will allow for primary and secondary outcomes assessment.

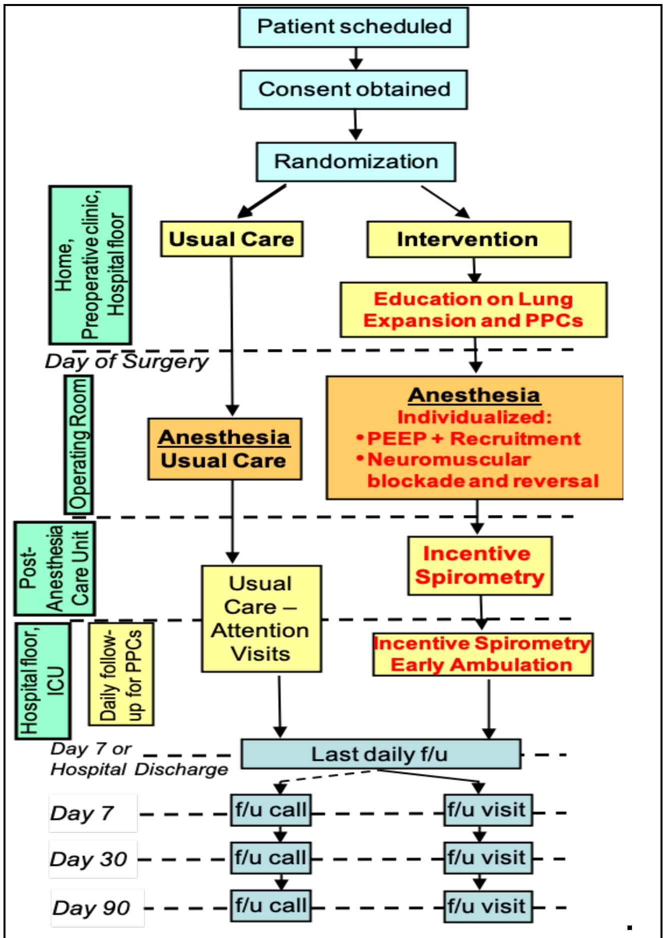

Fig. 2 Diagram of **PRIME AIR Trial.** Bundle interventions are presented in red.

Outcomes will be collected through hospital visits, videoconference or phone calls (if the patient is discharged from hospital) immediately after postoperative days (POD) 7, 30

and 90. The visit (in-person or videoconference) or phone call for POD7 (with medical record review if required) will provide primary and secondary outcomes, and the follow-ups after POD 30 and 90 will correspond to secondary outcomes. Participation will be concluded after the POD 90 follow-up visit (in-person or videoconference) or phone call. Windows will be allowed for each postoperative assessment: postoperative days 7 to 14 for the assessment of POD 7, postoperative days 30 to 45 for POD 30, and postoperative days 90 to 120 for POD 90.

Study Protocol for the Intervention Group – Perioperative Anesthesia-Centered Bundle Elements:

Preoperative Phase:

Lung Expansion **E**ducation:

Because incentive spirometry and early ambulation depend on patient performance, patients in the Intervention group will receive study-specific preoperative education consisting of:

A. Educational printed material and video: These materials will be provided to patients before their surgery. The materials will contain written and pictorial information on the importance of postoperative pulmonary complications and their prevention with adequate lung expansion after surgery using incentive spirometry and early ambulation. The effects of anesthesia and surgery on lung volumes and its consequences for lung dysfunction and infection will be explained. The educational materials have been designed to encourage patients to adhere to interventions by emphasizing that their participation is vital to protect their lungs. The written information is included in Appendix II and the educational video is available at https://youtu.be/ZcNRZSqjgRw.

B. In-person or videoconference education: Reinforcement of these concepts, answers to questions and practice with an incentive spirometer will be done at the time of visit to the anesthesia/surgery preoperative clinic, as videoconference, or on the day of surgery before transport to the operating room and administration of any sedatives. Members of the research team will implement this education intervention to maximize patient adherence. Family members and caregivers, if present, will be involved in the instruction to enhance involvement and further encourage adherence to therapy.

Intraoperative Phase:

Managing anesthesiologists will be those usually composing the team caring for abdominal surgery patients at the participating sites. Anesthesiologists will be randomized into two groups, caring only for either controls or interventional patients, to minimize contamination, i.e., avoid anesthesiologists being exposed to the bundle and then treating control patients. The site PI or study coordinator will be available in the operating room to monitor and provide support with the interventions. This phase will consist of two components (Fig. 1):

A. <u>**P**EEP and Lung **R**ecruitment - Mechanical Ventilation with **I**ndividualized PEEP:</u>

Perioperative lung expansion will be accomplished by PEEP individualization through maximization of the patient's respiratory system compliance (C_{RS}). This corresponds to the minimization of driving pressures, i.e., the difference between plateau pressure and PEEP during mechanical ventilation. This ***maximal compliance strategy*** will be started immediately after induction and repeated at least hourly throughout the surgery (see Appendix III for additional details)***.***

PEEP individualization will be determined by a recruitment maneuver followed by a decremental PEEP titration (Fig. 3). The stepwise **recruitment maneuver** will consist of the increase of PEEP from 5 to 20 cmH_2O in 5 cmH_2O steps of 30-sec each, as long as the patient's plateau pressure (P_{plat}) =30-32 cmH_2O (PEEP can be increased by 2-3 cmH_2O instead of by 5 cmH_2O to achieve P_{plat} =30-32 cmH_2O). Starting at this maximally achieved PEEP, PEEP will be decreased in 3 cmH_2O steps of 45-75 sec until a maximum C_{RS} is identified by a reduction in C_{RS} at a lower PEEP following a larger C_{RS} at a higher PEEP (Fig. 3). The minimum PEEP along the decremental steps will be 2 cmH_2O. Once the PEEP for maximal C_{RS} is identified, the largest PEEP level achieved during the recruitment maneuver will be used for 30-sec (in order to reinforce lung recruitment), followed by setting PEEP as the value that maximized C_{RS}. The procedure will be repeated on an hourly basis, and following any significant event producing a ≥15% reduction in C_{RS} (e.g., significant change in surgical table position, application of surgical retractors, endotracheal tube disconnection, endotracheal tube suction). Similar adjustments will be performed in

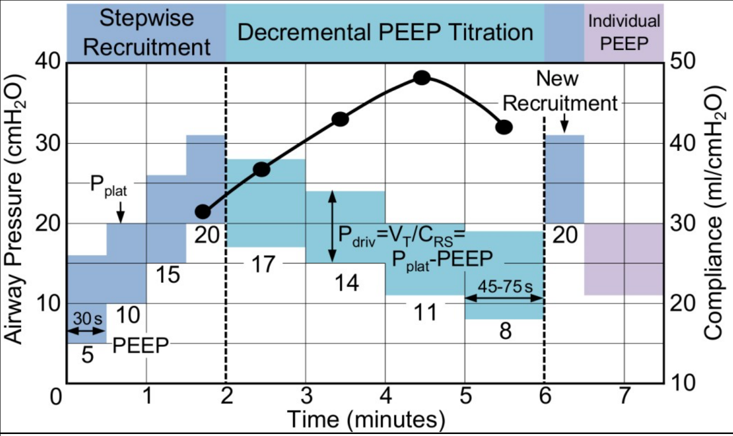

Fig. 3. Decremental PEEP titration protocol following a stepwise recruitment maneuver. Respiratory system compliance (C_{RS}) is measured at each step (●) and the PEEP corresponding to the maximum compliance is set as the individualized PEEP. In this example, titration would be stopped at 8 cmH_2O and the final PEEP set at 11 cmH_2O.

case of □15% C_{RS} increases (i.e. removal of retractors) in order to avoid overinflation if triggers of lung derecruitment are removed.

Other mechanical ventilation settings will be: volume controlled ventilation, tidal volume (V_T)=6-8mL/kg of predicted body weight, F_IO_2 titrated for the target SpO_2≥96% (F_IO_2 increased only to maintain that target while avoiding hyperoxia), I:E ratio 1:2 and respiratory rate aiming at normocapnia ($P_{ET}CO_2$=32-42 mmHg and/or P_aCO_2=35-45 mmHg, if available).

B. **M**uscle relaxant optimization – Individualized Administration and Reversal of Neuromuscular Blocking Agents:

Only intermediate-acting neuromuscular blocking agents will be used (Table 2). Induction doses of muscle relaxants will be routine: Cisatracurium (≤0.2 mg/kg), rocuronium (≤1.2 mg/kg), vecuronium (≤0.1 mg/kg). Given the difficulty of precise dosing due to the presentation of the medications, doses 25% higher (or to the next round mL, e.g., 10 mg for rocuronium, 2 mg for cisatracurium, 1 mg for vecuronium with usual presentations) will be considered as consistent with the intent of the protocol. Succinylcholine can be used and dosed at the anethesia team's discretion. Maintenance doses will be administered as required by surgical conditions and following confirmation of the presence of twitches in the train-of-four (TOF) stimulation. Readministration of muscle relaxants will be avoided as possible when <2 twitches are present unless specific clinical conditions such as diaphragmatic contraction indicate otherwise. Maintenance doses will be limited to (Table 2): Cisatracurium (≤0.03 mg/kg), rocuronium (≤0.2 mg/kg) and vecuronium (≤0.015 mg/kg). As described for the induction dose, we will consider doses ≤25% higher than the target dose or to the next round dose in mililiters as consistent with the intent of the protocol.

Table 2. Maximal intubating and maintenance doses of muscle relaxants to be used during the PRIME-AIR trial.

	Dose (mg/kg)	
	**Intubation**	**Maintenance**
Cisatracurium	0.2	0.03
Rocuronium	1.2	0.2
Vecuronium	0.1	0.015

Table 3. Doses of neuromuscular blockade reversal based on monitoring of the train-of-four (TOF) ratio (T4/T1).[113, 171]

Twitches in the TOF		Neostigmine dose (mcg/kg)	Sugammadex dose (mg/kg)
**Quantitative Monitoring**	**Qualitative Monitoring**		
0	0	Wait	Wait or 4-16
1	1	Wait	3-4
2-3	2-3	50	2-3
T4/T1<0.4	4 with fade	40	1-2
T4/T1=0.4-0.9	4 without fade	15-25	0.25-2
T4/T1>0.9		Unnecessary	Unnecessary

Monitoring of neuromuscular transmission with a peripheral nerve stimulator will be applied in all cases using the assessment of muscle response to a TOF stimulation

(series of four electric stimuli delivered at 2 Hz). Ulnar nerve stimulation with assessment of adductor pollicis response will be preferred. If not achievable, stimulation of the temporal branch of the facial nerve with orbicularis oculi response will be monitored[38]. We will encourage use of acceleromyography-guided quantitative TOF monitoring. Yet, given current practice, we designed this intervention to allow for use of either qualitative (based on visual or tactile assessment of the TOF response) or quantitative neuromuscular blockade monitoring. The following principles will be followed for neuromuscular blockade reversal management according to type of monitoring:

B.1. Quantitative monitor (e.g., acceleromyography): In this case, at least 90% recovery of the ratio between the fourth and first twitches (T4/T1>0.9) of the TOF stimulation will be ensured before extubation. If this is present before administration of reversal agents, no neuromuscular blockade reversal is required. However, up to 1 mg/kg of sugammadex is allowed. If it is not present (i.e., T4/T1≤0.9), reversal agents will be administered based on the TOF quantitative response according to Table 3. As for the doses of neuromuscular blockers, we consider doses not more than 25% above the target dose, or the next higher dosing interval (50 mg for total dose of sugammadex; 1 mg for total dose of neostigmine) as consistent with the protocol. Extubation will only be performed once a T4/T1>0.9 is achieved.

B.2. Qualitative monitor (visual or tactile assessment of the TOF response): (i) Neostigmine users: administered only when at least two twitches are present in the TOF stimulation and based on the doses presented in Table 3; (ii) Sugammadex users: dose based on TOF count as recommended following rocuronium- or vecuronium-induced neuromuscular blockade (Table 3). As described above for quantitative monitoring, higher doses will be considered consistent with the intent of the protocol if not more than 25% above the recommended dose or the total dose is not more than the next highest dosing interval.

High-dose neostigmine (≥60 mcg/kg) will be systematically avoided since it can increase the risk for muscle weakness and postoperative complications[12,39].

Additional signs of patient responsiveness, such as ability to follow commands, and appropriate hemodynamic, respiratory and temperature parameters will be recommended before extubation.

Postoperative Phase:

A. Incentive spirometry: Our protocol has been designed to address concerns that incentive spirometry in usual care is not systematically monitored and adherence is low[32, 5]. To maximize adherence and benefit, intervention patients will receive:(1) specific preoperative education as described above, (2) clear expectations of 10 incentive spirometry breaths every hour for at least 10 hours along the day (i.e., 10 sessions total with 100 incentive spirometry breaths per day); and (3) direct supervision of performance targeted to 3 of those 10 sessions, with an expectation that this will be done at least twice each day, spaced along awake hours.

Instructions will be based on the Guidelines of the American Association for Respiratory Care (AARC)[40]. The patient will be instructed to hold the spirometer in an upright position, exhale normally, and then place the lips tightly around the mouthpiece. This will be followed by a slow inhalation to raise the ball (flow-oriented) or the piston/plate (volume-oriented) in the chamber to the set target. At maximum inhalation, the mouthpiece is removed, followed by an at least 5-sec breath-hold and normal exhalation. Instruction of relatives, guardians, and other health caregivers in the technique will be provided to facilitate the patient's appropriate use of the technique and assist with encouraging adherence to therapy.

A member of the research team will be the supervisor aiming to maximize patient's performance through education, reinforcement of expectations, and resolution of compliance barriers. Supervision will be started within 2 hours after arrival at the postanesthesia care unit and continued in the floor until the patient is able to freely ambulate. Free ambulation will be defined as the ability to be out of bed for at least 6 hours/day and walk at least 75 meters 3 times/day[41,42]. Postoperative supervision visits can also be performed by using a site-specific HIPAA-compliant telehealth videoconferencing platform.

Preliminary studies from our group and previous investigations with targeted motivational education have shown improved adherence to therapy in respiratory conditions with traditionally poor patient compliance[43,44].

<u>B. Postoperative **A**mbulation:</u>

As with incentive spirometry, early ambulation is routinely pursued after surgery but not always systematically implemented or reported (although it is more frequently systematically used than incentive spirometry in our participating sites). Early mobilization and ambulation of intervention patients will be done by health care providers participating in the patient's routine care. The same member of the research team supervising incentive spirometry will support ambulation through increased education, reinforcement, systematic monitoring (3x/day), and facilitation of barriers (e.g., pain) by advising the patient to communicate with the health care team until free ambulation. Ambulation after surgery is part of usual care in more than 50% of the planned sites (Table 4) and we will encourage early ambulation depending on resources routinely available at each individual site (e.g. nursing, physical therapists). As tolerated, the following specific expectations for mobilization will be expressed to the patient and pursued: postoperative day (POD) 0 (starting from end of surgery until midnight) - up in chair with assistance; POD 1 - up in chair 3 times/day for meals, ambulation in room or hallway 2 times/day with assistance; POD 2 - out of bed for all meals and >6 hours during the day, walking in hallway more than 15 meters 3 times/day with assistance; POD 3 - increase ambulation (desirably beyond 75 meters) 3–5 times/day.

Protocols to guide the basic requirements for patient care and safety during mobilization and ambulation are available in all sites, and followed by postoperative health care providers. In the rare circumstances that a contraindication for early mobility is present, the intervention will only be implemented when that contraindication is resolved. Contraindications include: vasopressor requirement, acute neurologic event, femoral catheters, open abdominal wounds, and excessive sedation.

Performance on postoperative incentive spirometry and ambulation will be directly noted during the visits (in-person or videoconference) by an unblinded investigator. Although the target is for the unblinded investigator to visit the participant 3 times a day, we will consider 2 in-person visits with incentive spirometry to meet the target for the study.

Concomitant Interventions:

If pain or other barriers are identified that prevent/limit adequate performance of incentive spirometry or early ambulation, the investigator will advise the subject to report this to their care team. Subsequent execution of incentive spirometry/ ambulation will be pursued as soon as feasible.

Table 4. Usual care for interventions in 14 US academic centers

**Usual Care Distribution**	Mostly	Frequently	Occasion	Seldom
**% receiving intervention**	**>80%**	**50-80%**	**20-50%**	**< 20%**
Pre-op Education	29	21	**7**	**43**
Individualized PEEP	0	7	**14**	**79**
Reversal protocol	21	29	**14**	**36**
Reinforced Inc. Spirometry	29	14	**14**	**43**
Consistent Early Ambulation	50	21	14	14

The following perioperative management goals will be flexibly defined for participants in the intervention group to avoid both extremes of clinical practice and practice misalignment: (a) anesthesiologists will be advised to use continuous positive airway pressure of at least 5 cmH_2O during preoxygenation for induction of anesthesia, and during the period of spontaneous ventilation preceding removal of the endotracheal tube; (b) F_IO_2 should target normoxia ($SpO_2 \geq 96\%$). Hyperoxia should be avoided (i.e., the lowest range of F_IO_2 required to achieve that target should be used, and increases in F_IO_2 done only to maintain that target SpO_2); (c) fluid administration will be aimed at neutral intraoperative fluid balance, and total recommended limit ≤6 mL/kg/h including pre-operative (correction of dehydration) and intraoperative administration. Hemodynamic management based on goal directed fluid therapy to guide vasoactive medications and avoid excessive fluid administration will be suggested to the primary care team as a consideration in high-risk cases if patients present no response after two fluid boluses. The primary care team will also be advised to maintain neutral fluid

balance in the immediate postoperative period (first 24 hours) while oral intake is not restarted.

<u>Postoperative Mechanical Ventilation (if applicable):</u> The pre- or intraoperative decision to transfer a patient to the critical care unit will be made by clinical staff and recorded. To maintain the basic tenet of a recruited lung into the postoperative period, patients requiring mechanical ventilation postoperatively will receive at least one PEEP titration maneuver per day as described above and in keeping with interventions utilized in ICU patients[45]. This is consistent with the evidence on the beneficial effect of low driving pressures to outcomes of surgical[8,46] and ICU patients[47]. Of note, recruitment maneuvers and PEEP levels in our protocol are substantially different from those utilized in the ART trial and lead to values comparable to the control lower-mortality arm of that trial[48]. We will monitor if the PEEP optimization maneuver and the resulting PEEP used at least once a day. In general, this or alternative methods based on principles of lung expansion should be attempted if considered feasible by the ICU clinical team. Mechanical ventilation in the ICU will be done at the discretion of the critical care team. Recruitment maneuvers and PEEP titration are part of the usual care of mechanically ventilated ICU patients, and not expected to constitute a significant challenge to care providers.

<u>Study Protocol for the Control Group:</u>

The control group will consist of a *usual care* group, managed in a manner consistent with routine perioperative medical practice at the participating hospitals and of the attending anesthesiologists. This will increase the pragmatic character of the trial, as well as generalizability of our results. This design improves safety monitoring by providing an ability to detect harmful effects relative to usual care. It also strengthens the findings of the trial by emphasizing their pragmatic meaning. Details of usual care in participating sites are described in Table 4. This survey of practices in our 14 participating sites indicated that usual care corresponded to minimal individualization of intraoperative PEEP, infrequent use of any systematic management of neuromuscular blockade, unusual enforcement of incentive spirometry and rare preoperative education.

Participants in the control group will also be visited (in-person or videoconference) or called in the preoperative settings, post-anesthesia care unit, and postoperatively at a frequency equivalent to that of the intervention group to reduce the likelihood of bias related to less personal attention. At those points, patients will be greeted, and a generic conversation held.

<u>Study Design Aspects Relevant To All Participants:</u>

With the exception of the specific intervention elements, anesthetic and postoperative management will follow each participating site's routine clinical practice, according to

the choices of the primary clinical team caring for the patients. Fluid therapy and intraoperative fluid balance (crystalloids, colloids, and volume loss) will be recorded for analysis. Participants will receive standard local measures to maintain oxygenation, hemoglobin, core temperature, blood pressure and heart rate. Postoperative analgesia will be typically provided by epidural infusion (bupivacaine ± opioid) or intravenous patient controlled opioid infusion. Oxygen supplementation postoperatively is recommended to be administered if SpO_2<94% (measured at rest in room air).

Blood Biomarker Analysis and Biobank:

We will collect blood samples perioperatively at three specific time points (before surgical incision, at extubation ±30min at the end of surgery, and 24±12h after extubation). Blood collection (10mL at each time point for a total of maximum 30 mL per patient) will be synchronized with routine blood draws whenever possible to prevent extra needle sticks for the patient. All samples will be sent to the University of Colorado for storage as the PRIME-AIR biorepository and they will be analyzed for biomarkers of lung injury under the supervision of the study co-PI, Dr. Fernandez-Bustamante. The 10mL blood samples will be distributed in EDTA tubes (7.5mL) for plasma collection and PAXgene tubes (2.5mL) for RNA collection. The EDTA samples will be centrifuged at 1,000-2,000 rpm for 10 min, plasma volume aliquoted (0.5 mL aliquots), and frozen at 80°C until analysis. Following conclusion of the study and unless patients have opted out, samples of the PRIME-AIR biorepository will be submitted to the NHLBI Biologic Specimen and Data Repository (BioLINCC)) for the creation of a biobank for future studies on PPCs.

Study Outcomes

Primary Outcome: The primary outcome is the participant's PPC severity during the first 7 days after surgery. PPC severity will be classified from none to Grade 4 (Table 5) based on the most serious PPC occurring during those 7 days. Our focus on PPCs as the primary outcome is due to their major clinical relevance. This contrasts with previous use of composites of pulmonary and extrapulmonary outcomes to assess pulmonary interventions[7,49,50]. By studying the patient severity of PPCs as the primary outcome metric, we seek to detect the impact of the bundle not only on the PPC rate but also account for the bundle effect on the severity of occurring PPCs. We will utilize an established scale of PPC severity[7,51,52] (Table 5) that has been slightly modified to include PPCs defined in recent large trials[4,49,50]. These modifications aim to maximize event detection by minimizing gaps in the list of conditions composing the primary outcome through the addition of two items: (a) Mild hypoxemia (Grade 1), a usual clinical trigger for oxygen therapy in acute patients[26,49,49,53], associated by us and others with clinically meaningful outcomes and health care utilization; and (b) Respiratory infection (Grade 2), included as an important complication in recent large studies on PPCs with a broad definition[4,23,54]. Accordingly, the proposed modifications will enhance PPC detection. Indeed, a recent study by one of our collaborators showed an event rate

of 48% in the control arm (N=244) in a similar population[49]. The 7-day postoperative period was chosen because it includes the majority of PPCs occurring within the first month after surgery[7], known to substantially prolong hospital length of stay[55]; and it was used in previous large trials[7,23].

Table 5. Operational Definitions of PPCs[20]
**Grade 1**
Mild hypoxemia: SpO_2 =90-92% on room air or the equivalent imputed PaO_2/FiO_2 ratio when oxygen therapy is provided
Mild respiratory findings: abnormal lung symptoms/signs (e.g., cough, dyspnea) and temperature >37.5°C without other documented cause; chest radiograph normal/unavailable
**Grade 2**
Cough: productive, no other cause
Bronchospasm: new or pre-existent wheezing resulting in therapy change
Hypoxemia: PaO_2<60 mmHg or SpO_2< 90% on room air or the equivalent imputed PaO_2/FiO_2 ratio when oxygen therapy is provided
Respiratory Infection: use of antibiotic for suspected respiratory infection and at least one of the following: new or changed sputum, fever>37.5°C, WBC>12,000/mm^3
Atelectasis*: radiological confirmation + either temperature >37.5°C or abnormal lung symptoms/signs (e.g., cough, dyspnea) Hypercarbia: transient, requiring treatment
**Grade 3**
Pleural effusion: resulting in thoracentesis
Pneumonia**, suspected: radiological evidence without bacteriological confirmation
Pneumonia**, proved: radiological evidence and documentation of pathological organism
Pneumothorax: resulting in intervention.
Ventilatory dependence: (non-invasive or invasive ventilation) < 48h
**Grade 4**
Ventilatory failure: postoperative non-invasive or invasive ventilation dependence ≥48h
* **Atelectasis:** lung opacification with shift of the mediastinum, hilum or hemidiaphragm towards affected area and compensatory overinflation. ** **Pneumonia:** new and/or progressive pulmonary infiltrates on chest X-ray and two or more of: fever ≥38°C or hypothermia (<36°C); white blood cell count (WBC)/mm^3>12000 or <4000; purulent sputum and/or onset or worsening cough or dyspnea.

Secondary Outcomes (further defined in Appendices I)**:** We will study Grade 3 and 4 PPCs (more reliably determined at unsupervised timepoints) at 30 and 90 days to verify if short term (7-day) effects persist at meaningful mid-term periods. We will assess a sub-set of individual PPCs to identify specific effects, relevant for analysis and clinical

management. Because lung injury is associated with extrapulmonary complications[7,28], we will quantify a subset of those. Given that PPCs have a major effect on hospitalization costs[58], we intend to collect different measures of health care resource utilization. Hypoxemia and hypotension are related to the proposed interventions and important intraoperative events requiring quantification. Specifically, the following 13secondary outcomes will be assessed:

- Proportion of participants with highest grade being Grade 1 or 2 through POD 7
- Proportion of participants with highest grade being Grade 3 or 4 through POD 7, 30, 90
- Proportion of participants with Hypoxemia by POD 7 (as defined in PPCs Grade 1 and 2),
- Proportion of participants with Atelectasis by POD 7;
- Proportion of participants with Pneumonia (suspected and proved) by POD 7;
- Proportion of participants with Ventilatory dependence or failure by POD 7;
- Proportion of participants with both intraoperative hypoxemia and rescue recruitment maneuvers;
- Proportion of participants with intraoperative cardiovascular events (hypotension, bradycardia, tachycardia, arrhythmias, new ST-segment changes and cardiac arrest);
- Length of postoperative oxygen therapy/other respiratory support;
- Length of hospital stay;
- Proportion of participants with any major extrapulmonary complications (as defined in Appendix I by established criteria[59–61]); .

Exploratory Outcomes (further defined in Appendices I and IV)**:** These include 7 and 30-day mortality, as it is affected by PPCs[4], and 90-day mortality, important for high-risk patient subsets such as the elderly[56], and for accurate estimation of mortality after major abdominal surgery[57]. We will assess the subset of individual PPCs not included in the secondary outcomes to identify specific effects, relevant for clinical management and interpretation of the results. Because lung injury is associated with extrapulmonary complications[7,28], we will quantify a panel of those as defined in Appendix I. Patient reported outcomes are increasingly emphasized as key data to determine management. Thus, we will collect those addressing dyspnea and fatigue. Specifically, the following exploratory outcomes will be assessed:

- All cause mortality at postoperative days 7, 30 and 90;
- Each of the individual components of the list of pulmonary complications in the primary endpoint not included in the secondary outcomes (Table 5);
- Rate of intraoperative muscle weakness;
- Dose of intraoperative vasoactive medications, crystalloids and colloids;
- Length of stay in the postanesthesia care unit;
- Unexpected admission to ICU;
- Length of ICU stay;

- Rate of each of the major extrapulmonary complications (defined in Appendix I by established criteria[59–61]);
- Rate of hospital readmission(s) after initial discharge;
- Patient-Reported Outcomes Measurements (PROMIS®) short forms (Appendix IV) addressing: Dyspnea Characteristics, Severity and Functional Limitations; and Fatigue, before surgery and 7, 30 and 90 days after surgery.[62,63].

Clinically important differences for primary and secondary outcomes: Because a large number of patients are affected by PPCs and undergo abdominal surgery, even small percent differences in the outcomes to be studied represent a large absolute number of patients that could be affected by the studied intervention. Accordingly, we will consider clinically significant: (a) a 10% difference in the primary outcome of the study (the row mean score test of a Cochran-Mantel-Haenszel, which incorporates both the frequency and highest grade of severity of PPCs during the first 7 days after surgery; (b) 5% difference in the rate of composite or isolated PPCs; (c) 5% difference in the rate of intraoperative and major extrapulmonary complications; (d) a difference of 12h in the length of postoperative oxygen therapy/other respiratory support; (e) a difference of half-day in the length of hospital stay.

Plasma biomarkers. We hypothesize that the PRIME-AIR bundle will minimize lung mechanical injury and inflammation by reducing overinflation and atelectasis. Accordingly, it will reduce plasma levels of biomarkers of inflammatory, epithelial and endothelial injury. Plasma will be collected from all patients for analysis of a focused panel of plasma biomarkers of inflammation (cytokines IL-6, IL-8); epithelial injury (the soluble form of the receptor for advanced glycation end-products, RAGE, and club cell protein 16, CC16), and endothelial injury (angiopoietin-2, Ang-2). We will compare biomarker levels in the PRIME-AIR bundle *vs.* control groups before, at the end of and 24h after surgery. Blood collection in this aim will not only allow for the biomarkers analysis, but also for the creation of a biobank for future studies on PPCs.

Blinding: Because the intraoperative and postoperative interventions cannot be blinded, each site will have at least 2 members of the research team: one to perform the randomization, education, and implementation of the protocol in the operating room and postoperative incentive spirometry/mobilization during hospital visits (in-person or videoconference); and another, blinded to patient allocation, to assess PPCs and all other outcomes -with information obtained from the participant's medical chart and phone calls to the participant (phone script in Attachments/Insight). Investigators performing biomarker assays will also be blinded to study group.

VI. STATISTICAL ANALYSIS

A detailed statistical analysis plan (SAP) will be prepared and approved by the core study team prior to locking the database for the study. The material below provides an

overview of the key features of the SAP. Should there be any discrepancy between the final SAP and material in the protocol, the final SAP will be the definitive version.

Justification of Sample Size. Sample size was estimated using simulations of the primary analysis of the primary outcome. Based on these simulations, our sample size is 375/group (750 total) being studied. We have formulated the treatment effect in this project as a relative percent reduction in PPC rate between groups, not an absolute difference between the two groups. With a PPC event rate of 40%, and a treatment effect (relative reduction of PPC rate) of 35.29% (so an absolute PPC event rate of 25.88% in the group receiving the intervention bundle), and no change in the distribution of the severity of PPCs, we would have 93% power (α= 0.05, two-sided) for the primary analysis. The 35.29% treatment effect was estimated from a Bayesian analysis as the lower 10% bound (one-sided) of the highest posterior region based on previous studies of aspects of the intervention; this means that we have a 90% expectation that the true treatment effect of the entire bundle would be greater than 35.29%. Power increases if the PPC event rate is higher than 40%.

Analytic approach - primary endpoints: The primary outcome is the participant's PPC severity using a previously published five-point scale[7,51,52]. Participants are graded from 0 (no PPC at all) to 4 (most severe PPC) based on the most serious PPC occurring during the first seven days after surgery. The analysis will use a Cochran-MantelHaenszel test (specifically the row mean score test), stratifying by site. This tests the null hypothesis that there is no difference between the two treatment groups in the distribution of the most severe PPC grade during the first seven days of the study. The alternative hypothesis is that there is a difference between the two treatment groups in the distribution of the most severe PPC grade during the first seven days of the study. The results of this test will determine whether the study can claim that the intervention bundle reduced the average level of PPC severity

We will also compare biological measures of lung function and injury between the interventions: (1) lung mechanics and gas exchange; and (2) plasma biomarkers of lung injury at baseline, end of and 24±4h after surgery (see secondary outcomes).

Minimizing bias in outcome assessment: Study of each subject will involve at least 2 investigators per center: one to perform the randomization and implementation of the protocol in the operating room, and another, blinded to patient allocation, to assess for postoperative complications.

Analytic approach - secondary endpoints: The type of variable of the secondary outcome determines the approach used, as listed in Table 6 below:

**Table 6.** Approach for primary analysis according to the type of variable	
Type of Variable	Approach for Primary Analysis

Binary	For rare events (≤ 5%): Fisher's Exact test<br>For more common events (>5%): Logistic regression (to allow for stratification by site)
Categorical (> 2 categories)	Cochran-Mantel-Haenszel test
Continuous	van Elteren test (an extension of the Wilcoxon Rank Sum test to allow for stratification by site)
Time to death	Cox Proportional Hazards Model
Repeated continuous outcomes (e.g. PROMIS® measures)	Mixed model Analysis of Variance

Additional analyses to fully explore the data will be specified in the SAP.

Interim analyses: We will perform two interim analyses after approximately 1/3 and 2/3 of participants have completed the 7-day follow up. Efficacy will be assessed using a Haybittle-Peto boundary of 0.001 at each analysis. We will be doing a futility analysis at the second interim analyses as well.

VII. RISKS AND DISCOMFORTS

The estimated risks and discomforts involved with participation in this study include:

I. Risks of recruitment maneuvers and PEEP titration: The potential main serious risks of the proposed bundle are hemodynamic instability, respiratory deterioration including pneumothorax. The PEEP values and protocol for recruitment maneuvers to be applied in the intervention arm of this study are comparable to those from previous studies and intensive care practice in early or established lung injury[64,65]. PEEP levels have been adjusted to lower maximal values in the current proposal given that lungs of surgical patients have better mechanical properties including recruitability as compared to those of intensive care patients. We are also excluding patients with conditions with the potential to increase the susceptibility to complications. In addition, ventilator parameters will be changed in a manner aimed at minimizing the likelihood of complications, e.g., P_{plat} to be kept under 30-32 cmH_2O and PEEP to be changed in a stepwise manner aiming at minimizing significant and rapid hemodynamic instability. Given the previously observed safety in terms of similar recruitment and PEEP levels in critically ill patients[66,67], we anticipate safe implementation in patients with less critical conditions. Indeed, we have successfully used intraoperative PEEP titration in patients comparable to those to be enrolled in the proposed trial during our R34 grant, without any serious adverse event. Such expectation of safety is reinforced because the intervention will be administered in an intensely monitored environment, i.e., operating room.

Hemodynamic instability can be triggered by the increased intrathoracic pressure during a recruitment maneuver or multiple other reasons during surgery. Hemodynamic mild/moderate changes (possible risk) include different degrees of hypotension (systolic blood pressure <100 mmHg or mean blood pressure <60 mmHg), tachycardia (heart rate >100 bpm) or bradycardia (heart rate <60 bpm) and/or cardiac arrhythmias. Hemodynamic instability will be defined as systolic blood pressure >160 or <80 mmHg, heart rate >140 or <50 bpm, or deviations by more than 20% in any of those parameters from patient’s baseline for >3 minutes despite treatment (rare risks) or cardiac events (extremely rare) (e.g. significant arrhythmias, electrocardiogram ST segment changes). These risks are present during recruitment maneuvers and mechanical ventilation performed as standard of care during surgery. Our intervention is not expected to significantly increase these risks, as we include the assessment of hemodynamic stability before the initiation of recruitment maneuvers and careful titration of PEEP level. The alternative treatment includes maintaining low levels of PEEP, which can contribute to insufficient lung expansion, low oxygenation, and increased risk of pneumonia after surgery. Of note, stepwise recruitment as proposed in the current protocol is associated with less hemodynamic instability.

Respiratory deterioration, including: Inadequate oxygenation or desaturation, defined as a SpO_2<90% or a need to increase F_IO_2 by □0.2 to maintain the targeted SpO_2 □96%, hypercapnia ($P_{ET}CO_2$>45 mmHg, $PaCO_2$>50 mmHg or increase by 2mmHg in either parameter compared to before intervention), and increase in plateau pressure (P_{plat} >30-32cmH_2O) (possible risks). These risks are present during standard of care during surgery and can be related to recruitment maneuvers and mechanical ventilation performed but can also be due to other factors, e.g., hypotension, and bronchial secretions. Our intervention is not anticipated to significantly increase this risk. There are no alternative treatments that reliably prevent intraoperative respiratory deterioration, and we have designed the appropriate responses in our protocol to respond in the event respiratory deterioration occurs.

Sudden hypoxemia followed by hypotension will lead to the consideration of a possible trauma to the airways and pneumothorax (rare risk). The PEEP values and protocol for recruitment maneuvers to be used in this study are within ranges compatible with those used in previous studies and in clinical practice. Specifically, recruitment maneuvers to values of 40 cmH_2O have been published, whereas in this study values of plateau pressure of 30-32 cmH_2O (considered a safe limit in patients with acute lung injury) will be used for only 30 seconds. PEEP values in the range of 0-20 cmH_2O are used in clinical practice. Recent studies actually indicated that values of 8-12 cmH_2O may be necessary to prevent injurious alveolar derecruitment due to abdominal pressure. Such pressures are expected to be important in cases of abdominal surgery with necessary muscle paralysis for surgery, when pressure applied by abdominal contents, surgical retractors and/or operative field manipulation leads to compression and derecruitment of the neighboring lung. The alternative treatment includes maintaining low levels of PEEP without recruitment

maneuvers, which can contribute to lung derecruitment, low oxygenation, and increased risk of pneumonia after surgery. Of note, our proposed values for recruitment maneuvers are substantially different (lower) from those used in the recently published ART trial[48].

II. Risks of neuromuscular blockade monitoring and reversal: insufficient use of neuromuscular blockade agents can lead to inadequate surgical conditions, and their excessive use and/or insufficient (or excessive) reversal are associated with postoperative muscle weakness. Muscle weakness can lead to atelectasis, hypoxemia, swallowing problems, aspiration of saliva or gastric contents, coughing, pneumonia and need for ventilatory support. These are precisely the issues the proposed intervention attempts to address and the risk is hypothesized to be reduced in the intervention group. Any sign of muscle weakness will be notified to the primary team and recorded. Given that the control group will follow usual care practices, any safety issues should be detectable during interim analysis. There is no alternative of care since muscle paralysis is required for this type of surgery.

III. Risks of incentive spirometry and early ambulation: pain and lightheadedness related to hyperventilation and respiratory alkalosis. Other possible adverse effects are fatigue and exacerbation of bronchospasm. Most frequently these either do not occur or are mild and resolved by interruption of the procedure. If patients develop these symptoms during the study interventions, the intervention will be stopped temporarily and symptoms addressed. This may include advising the patient to requestpain medication from the primary team or resting temporarily. Interventions will be re-attempted when the symptoms resolve and patients are agreeable to them. The alternative of not performing or substantial delay in those interventions can contribute to insufficient lung expansion, low oxygenation, and increased risk of pneumonia after surgery.

IV. Discomfort and hematoma due to blood sampling: Three blood samples will be obtained for the study for the analysis of levels of biomarkers of lung injury. These risks are possible if a new vascular access is required but should be a minor temporary inconvenience. We will attempt to obtain these blood samples from existing vascular lines and/or simultaneous to the collection of other blood sample needed for clinical analyses. This risk is also present for routine medical care.

V. Risk to patient privacy: Patient information and blood samples are collected for this study. Patient information will be de-identified and assigned a study ID but there is a possible risk to the patient's privacy. A list linking patient names to their study ID will be kept separate from the data in secure storage. Deidentified data, after review by the NHLBI BioLINCC data repository staff, will be available through the NHLBI BioLINCC biorepository. The plan to redact or convert all PHI data (e.g. date of hospitalization will be converted to days after randomization; rare events will be grouped to minimize the risk of participant identification; site will not be identifiable from published or internal data) will be reviewed and approved by the NHLBI BioLINCC prior to being implemented. Patients will be allowed to opt-out of providing the biorepository of blood samples if they do not

desire to participate in the availability of their blood samples for future research on pulmonary conditions or other research.

VI. Risk of anxiety: Potential subjects present a possible psychosocial risk of developing anxiety by being asked to participate in a research study. We will minimize the risk of anxiety by assuring patients that: the study is based on sound medical reasoning and data, the study is entirely voluntary, they may withdraw and have usual care at any time, their doctors have the last say in the manner their care will be provided, and that their decision of whether or not to participate will in no way affect their care.

VII. Risk of distress: Participants are at risk of being distressed by one or more items in the questionnaire. We will minimize the risk of distress by reminding participants that they may skip any question if they would prefer not to answer.

We do not know the risk of fetal harm with the study protocol, thus, pregnant patients are excluded.

Informed Consent

Sites with the capability to create an eConsent with a phone script to consent subjects remotely prior to their admission for surgery will be allowed to do so. When used, this process will follow the exact wording as the approved informed consent form (ICF), and that ICF will be placed in a HIPAA-compliant platform that creates a secure link. A phone script to assess subjects' interest will be used, and, if subjects are interested, they will receive a link to eConsent through secure email. Once the eConsent is signed, a copy will be attached to the patient's medical record.

Massachusetts General Hospital: Informed consent will be requested after confirmation of eligibility of inclusion and exclusion criteria of each potential participant. All questions and concerns will be addressed prior to obtaining Informed consent. Consent will be obtained in person in a private room by one of the licensed physician investigators listed on the protocol. Study nurses, coordinators, or other study staff may assist in the consent process by explaining the study to potential participants and answering any questions. All subjects will be given the opportunity to speak to a doctor during the consent process either in-person or through videoconference. Participants in this study must fulfill study criteria and be able to follow instructions of incentive spirometry and ambulation, and thus subjects whose cognitive status makes them unable to cooperate will not be eligible to participate. A copy of the informed consent will be provided to the patient. The original signed informed consent form will be kept locked in a secure location in the PI's or research coordinator's office. A MGH approved eConsent platform can be used. When used, this process will follow the exact wording as the currently approved ICF, and that ICF will be placed in a HIPAA compliant platform that creates a secure link. A phone script to assess subjects' interest will be used, and, if subjects are interested, they will receive a secure link to eConsent through

email. Once the eConsent is signed, a copy will be attached to the patient's medical record.

Brigham and Women's Hospital:

Informed consent will be requested after confirmation of eligibility of inclusion and exclusion criteria of each potential participant. Informed consent will take place in person in a private room of a medical center, or by phone conversation if the participant agrees to it or through videoconference. Consent will be obtained by one of the licensed physician investigators listed on the protocol. Study nurses, coordinators, or other study staff may assist in the consent process by explaining the study to potential participants and answering questions. Participants to this study must fulfill study criteria and be able to follow instructions of incentive spirometry and ambulation, and thus subjects whose cognitive status makes them unable to cooperate will not be eligible to participate in this study. A copy of the informed consent will be provided to the patient. The original signed informed consent form will be kept locked in a secure location in the BWH SICU Translational Research Center office. A BWH approved eConsent platform can be used. When used, this process will follow the exact wording as the currently approved ICF, and that ICF will be placed in a HIPAA compliant platform that creates a secure link. A phone script to assess subjects' interest will be used, and, if subjects are interested, they will receive a secure link to eConsent through email. Once the eConsent is signed, a copy will be attached to the patient's medical record.

University of Colorado:

Informed consent will be requested after confirmation of eligibility of inclusion and exclusion criteria of each potential participant. Informed consent will take place in person in a private room of a medical center, or by phone conversation if the participant agrees to it or through videoconference. Consent will be obtained by one of the licensed physician investigators listed on the protocol. Study nurses, coordinators, or other study staff may assist in the consent process by explaining the study to potential participants and answering questions. Participants to this study must fulfill study criteria and be able to follow instructions of incentive spirometry and ambulation, and thus subjects whose cognitive status makes them unable to cooperate will not be eligible to participate in this study. A copy of the informed consent will be provided to the patient. The original signed informed consent form will be kept locked in a secure location in the University of Colorado anesthesiology research personnel office. A University of Colorado approved eConsent platform can be used. When used, this process will follow the exact wording as the currently approved ICF, and that ICF will be placed in a HIPAA compliant platform that creates a secure link. A phone script to assess subjects' interest will be used, and, if subjects are interested, they will receive a secure link to eConsent through email. Once the eConsent is signed, a copy will be attached to the patient's medical record.

Duke University Health System:

The study coordinator or research nurse will confirm subject eligibility of inclusion and exclusion criteria. Once confirmed eligible, the study coordinator or research nurse will contact the potential subject either in person or via phone or through videoconference. Only key personnel trained in conducting informed consent will discuss the study with potential subjects. The participant will provide consent for themselves. The participant will be allowed as much time as they need to decide to participate or to decline. The consent process will take place in a designated private room or over the phone. All questions about the research study will be answered while with the patient. Contact information and a business card will be provided to the subject in the event there are additional questions. There will be no attempt at coercion or undue influence. Only subjects who are capable of performing the study criteria will be approached. Once consent is signed the original will be maintained in the subject's study file with a copy sent to medical records. A Duke University approved eConsent platform can be used. When used, this process will follow the exact wording as the currently approved ICF, and that ICF will be placed in a HIPAA compliant platform that creates a secure link. A phone script to assess subjects' interest will be used, and, if subjects are interested, they will receive a secure link to eConsent through email. Once the eConsent is signed, a copy will be attached to the patient's medical record.

Northwestern University/ Northwestern Medicine (NM):

Written informed consent will be requested after confirmation of eligibility of inclusion and exclusion criteria of each potential subject. Questions and concerns will be addressed by the research study team prior to obtaining written informed consent. Written consent will be obtained in person on the day of surgery in a NM hospital room by one of the study team members listed in the EIRB.
Participants in this study must fulfill study criteria and be able to follow instructions of incentive spirometry and ambulation, and thus subjects whose cognitive status makes them unable to cooperate will not be eligible to participate. A copy of the written informed consent will be provided to the patient. One copy will be forwarded to the medical records department of NM and the original signed informed consent form will be kept locked in a secure location in Arkes Pavilion 10 Floor Anesthesiology Research office in a locked storage room which is key card and hard key controlled. A Northwestern University approved eConsent platform can be used. When used, this process will follow the exact wording as the currently approved ICF, and that ICF will be placed in a HIPAA compliant platform that creates a secure link. A phone script to assess subjects' interest will be used, and, if subjects are interested, they will receive a secure link to eConsent through email. Once the eConsent is signed, a copy will be attached to the patient's medical record.

Beth Israel Deaconess Medical Center:

Once a patient is found eligible, he or she will be approached by a member of the research team for informed consent either in-person or through videoconference. Informed consent will take place in the preoperative setting, in the cardiac surgery clinic, inpatient floors or PAT clinic. The study will be discussed and the consent form reviewed. The subjects will have the opportunity to ask any and all questions, and are free to decline participation at any time. Written informed consent will be obtained prior to any research procedures. Copies of the signed consent will be provided to the subject and filed in the medical record while the original is retained by the study personnel to be filed in the patient's study folder kept at a secure location.

The research staff undergoes a rigorous consent training process. Within the Center for Anesthesia Research Excellence (CARE) at BIDMC, this training includes: didactic sessions, mandated attendance at CCI/HSPO seminars related to the informed consent process, shadowing of informed consent in a variety of contexts, trainee-led informed consent conversations with the aid of consenting checklists and accompanied by senior staff member and/or PI, robust feedback sessions, and clear communication when the team member is skilled enough to engage in informed consent discussions without direct supervision.

A BIDMC approved eConsent platform can be used. When used, this process will follow the exact wording as the currently approved ICF, and that ICF will be placed in a HIPAA compliant platform that creates a secure link. A phone script to assess subjects' interest will be used, and, if subjects are interested, they will receive a secure link to eConsent through email. Once the eConsent is signed, a copy will be attached to the patient's medical record.

Stanford Hospital and Clinics:

Informed consent will be requested after confirmation of eligibility of inclusion and exclusion criteria of each potential participant. All questions and concerns will be addressed prior to obtaining Informed consent either in-person or through videoconference. Consent will be obtained in person in a private room by one of the licensed physician investigators listed on the protocol. Study coordinator may assist in the consent process by explaining the study to potential participants and answering any questions. All subjects will be given the opportunity to speak to a doctor during the consent process. Participants in this study must fulfill study criteria and be able to follow instructions of incentive spirometry and ambulation, and thus subjects whose cognitive status makes them unable to cooperate will not be eligible to participate. One copy of the informed consent will be provided to the patient and other will remain as part of patient's medical record. The original signed informed consent form will be kept locked in a secure location in the PI's or research coordinator's office. A Stanford University approved eConsent platform can be used. When used, this process will follow the exact wording as the currently approved ICF, and that ICF will be placed in a HIPAA compliant platform that creates a secure link. A phone script to assess subjects' interest

will be used, and, if subjects are interested, they will receive a secure link to eConsent through email. Once the eConsent is signed, a copy will be attached to the patient's medical record.

UCSF Medical Center at Mission Bay:

Informed consent will be requested after confirmation of eligibility of inclusion and exclusion criteria of each potential participant. All questions and concerns will be addressed prior to obtaining Informed consent either in-person or through videoconference. Consent will be obtained in person in a private room by one of the licensed physician investigators listed on the protocol. Study nurses, coordinators, or other study staff may assist in the consent process by explaining the study to potential participants and answering any questions. All subjects will be given the opportunity to speak to a doctor during the consent process. Participants in this study must fulfill study criteria and be able to follow instructions of incentive spirometry and ambulation, and thus subjects whose cognitive status makes them unable to cooperate will not be eligible to participate. A copy of the informed consent will be provided to the patient. The original signed informed consent form will be kept locked in a secure location in the PI's or research coordinator's office. A UCSF approved eConsent platform can be used. When used, this process will follow the exact wording as the currently approved ICF, and that ICF will be placed in a HIPAA compliant platform that creates a secure link. A phone script to assess subjects' interest will be used, and, if subjects are interested, they will receive a secure link to eConsent through email. Once the eConsent is signed, a copy will be attached to the patient's medical record.

University of Rochester Medical Center:

Informed consent will be requested after confirmation of eligibility of inclusion and exclusion criteria of each potential participant either in-person or through videoconference. All questions and concerns will be addressed prior to obtaining Informed consent. Consent will be obtained in person in a private room by one of the research staff members certified to consent participants and listed on the study staff log for this protocol.

Study nurses or other study staff may assist in the consent process by explaining the study to potential participants and answering any questions. All subjects will be given the opportunity to speak to a study staff member during the consent process or to take the consent form home to review prior to signing consent. A copy of the signed informed consent will be provided to the patient. The original signed informed consent form will be kept locked in a secure location in the Anesthesia Clinical Research Center office. A URMC approved eConsent platform can be used. When used, this process will follow the exact wording as the currently approved ICF, and that ICF will be placed in a HIPAA compliant platform that creates a secure link. A phone script to assess subjects' interest

will be used, and, if subjects are interested, they will receive a secure link to eConsent through email. Once the eConsent is signed, a copy will be attached to the patient's medical record.

Mayo Clinic Rochester:

Informed consent will be requested after confirmation of eligibility of inclusion and exclusion criteria of each potential participant. Informed consent will take place in person in a private room of a medical center, or by phone conversation if the participant agrees to it. Consent will be obtained by one of the study team members listed on the protocol. Study nurses, coordinators, or other study staff may assist in the consent process by explaining the study to potential participants and answering questions. Participants to this study must fulfill study criteria and be able to follow instructions of incentive spirometry and ambulation, and thus subjects whose cognitive status makes them unable to cooperate will not be eligible to participate in this study. A copy of the informed consent will be provided to the patient. The original signed informed consent form will be kept locked in a secure location in the Mayo Clinic Rochester's Anesthesia Clinical Research Unit office. The study team members who will meet with the prospective subject and obtain informed consent must be sufficiently trained, knowledgeable about the research project in order to answer questions posed by the subject and must have IRB approval to obtain consent.

Memorial Sloan Kettering Cancer Center:

Informed consent will be requested after confirmation of eligibility of inclusion and exclusion criteria of each potential participant. All questions and concerns will be addressed prior to obtaining Informed consent. Consent will be obtained in person in a private room by one of the investigators listed on the protocol. Research nurses, coordinators, or other research staff may assist in the consent process by explaining the study to potential participants and answering any questions. All subjects will be given the opportunity to speak to a doctor during the consent process. Participants in this study must fulfill study criteria and be able to follow instructions of incentive spirometry and ambulation, and thus subjects whose cognitive status makes them unable to cooperate will not be eligible to participate. A copy of the informed consent will be provided to the patient. The original signed informed consent form will be kept locked in a secure location in the PI's or research coordinator's office and a copy will be scanned to the patient's electronic medical record.

University of Massachusetts Medical School- Hospital System:

Informed consent will be requested after confirmation of eligibility of inclusion and exclusion criteria of each potential participant. All questions and concerns will be

addressed prior to obtaining Informed consent. Consent will be obtained in person or virtually in a private room by one of the members of the study staff. Study nurses, coordinators, or other study staff may assist in the consent process by explaining the study to potential participants and answering any questions. All subjects will be given the opportunity to speak to a doctor during the consent process. Participants in this study must fulfill study criteria and be able to follow instructions of incentive spirometry and ambulation, and thus subjects whose cognitive status makes them unable to cooperate will not be eligible to participate. A copy of the informed consent will be provided to the patient. The original signed informed consent form will be kept locked in a secure location in the PI's or research coordinator's office/laboratory.

Columbia University Irving Medical Center:

Informed consent will be requested after confirmation of eligibility of inclusion and exclusion criteria of each potential participant. Informed consent will take place in person at the medical center, or by phone conversation if the participant agrees to it. Consent will be obtained by one of the study team members listed on the protocol. Study nurses, coordinators, or other study staff may assist in the consent process by explaining the study to potential participants and answering questions. Participants to this study must fulfill study criteria and be able to follow instructions of incentive spirometry and ambulation, and thus subjects whose cognitive status makes them unable to cooperate will not be eligible to participate in this study. A copy of the informed consent will be provided to the patient. The original signed informed consent form will be kept locked in a secure location in the Columbia University Irving Medical Center's Anesthesia Clinical Research Unit office.

Cleveland Clinic Foundation:

We are requesting approval for virtual consent for this study as well as approval of a phone script to use in the consenting process.
Please see below for a summary of the remote consent process:
The main way we plan to consent will be remotely via DocuSign and this process is described here. There will be a focus on obtaining patient consent remotely. We are proposing to use the new DocuSign (Part 11 Compliant) platform that will allow a patient to provide consent remotely via electronic signature. The process of obtaining consent will be as follows:

- Once the patient has been determined as potentially eligible, they will be contacted via remote platform (telephone or video platform) for a discussion regarding this study using an IRB approved phone script. A member of the study team will verify the patient's identity, present the consent via email with IRB approved cover letter language (with the patients approval to receive e-mail and verifying their email address) along with instructions for using DocuSign and answer any initial questions the patient may have.

The patient will be given sufficient time to review the consent. After the patient has reviewed and has expressed interest in the trial, one of the clinical team members will again review the consent via remote platform (telephone or video platform), answer any remaining questions, and ensure the patient's understanding. That clinical team member will then provide instructions on how the patient will sign the consent via the DocuSign platform. The patient will sign the consent electronically. The clinical team member will then provide their signature as the person obtaining consent electronically. The clinical team member will document the entire process, including whether the patient consented to the trial, in the patient's electronic medical record. The patient will be provided a copy of the signed informed consent form.

Any patient not signing consent via DocuSign will follow this procedure for in-person consenting:
- Once a patient has been determined to be potentially eligible for this study, a member of the study team will present the study as a potential treatment option to the patient during an in-person visit in a private room and answer any initial questions the patient may have. After the patient has had sufficient time to review the informed consent, the clinical research coordinator will again review the study with the patient, ensure the patient's understanding of the study and answer all of the patient's questions.

The study team member will then witness the patient signing the informed consent and will also sign the informed consent. The patient will be given a copy of the signed consent. The study team member will document the entire interview process per IRB Policy, including whether the patient consented to the trial, in the patient's electronic medical record.

Protections Against Risk

The study design implies a high degree of individual attention to each patient during the performance of the study. Consequently, safety is evaluated in a continuous basis during execution of the study. At least one physician or research coordinator of the study team will be available throughout the entire study.

As an overarching principle, the primary clinical team caring for the patients along the study (anesthesiologists, surgeons, and nurses) can intervene at any time if it is believed that patients are at risk.

The planned strategies for protecting participants from identified potential risks related to this study include:

I. <u>Hemodynamic instability:</u> The recruitment maneuver and PEEP adjustment will be interrupted if the patient becomes bradycardic or tachycardic, develops a new arrhythmia, or becomes severely hypotensive (systolic blood pressure < 80 mmHg or mean blood pressure <50 mmHg). The patient will be managed by the primary anesthesia team if any hemodynamic changes occur during the recruitment maneuver or decremental PEEP titration. PEEP levels will be decreased for any urgent clinical reasons at the discretion of the managing anesthesia team. In the absence of an emergency need for change, the

anesthesia team caring for the patient will consider addressing any reasons of hemodynamic changes with intravenous fluids, vasoactive medications, and/or a stepwise adjustment of PEEP consisting of reducing PEEP by 2-5 cmH_2O. The anesthesia team will reassess repeating the recruitment maneuvers and PEEP optimization once the patient is stable (heart rate <100 beats/minute, SBP>100 mmHg). Anesthesiologists are experienced at reducing or responding to these events during usual care for surgery.

II. <u>Respiratory deterioration:</u> It is at the primary anesthesiologist's discretion to change the protocol ventilator settings if any emergent respiratory deterioration is observed. In the absence of an emergency need for change, a new PEEP optimization will be performed. If it fails to improve SpO_2, F_IO_2 will be increased by 0.1-0.2 until a SpO_2□96% is obtained. If plateau pressures are beyond 30 cmH_2O, we will follow the guidelines provided by the ARDSnet to maintain plateau pressures below this threshold[17]: if P_{plat} >30 cmH_2O: decrease V_T by 1mL/kg PBW steps (minimum = 4 mL/kg PBW); if later P_{plat} <25 cmH_2O and V_T <6 mL/kg PBW, increase V_T by 1 mL/kg PBW until P_{plat} >25 cmH_2O or V_T=6 mL/kg PBW; if P_{plat} <30 cmH_2O and breath stacking or dyssynchrony occurs, V_T may be increased in 1mL/kg PBW increments to 7 or 8 mL/kg PBW as long as P_{plat} remains ≤30 cmH_2O.

III. <u>Pneumothorax:</u> Before and during any recruitment maneuver, signs of excessive airway pressure will be searched for to prevent barotrauma and pneumothorax. During recruitment maneuvers, sufficient depth of anesthesia will be ensured to minimize the risk of barotrauma caused by patients' gasping or opposing ventilation. Also, as mentioned before, plateau pressure ≤30 cmH_2O during ongoing ventilation will always be targeted. Anesthesiologists are experienced at reducing or responding to these events during usual care for surgery.

IV. Amongst the potential adverse effects, development of hemodynamic instability or worsened oxygenation to increases in PEEP to values ≥10 cmH_2O during 2 separate attempts will lead to discontinuation of intervention until susceptibility factors are corrected. Continuation will only be pursued if approved by the primary clinical care team. PEEP can be modified at any time at the primary anesthesiologist's discretion if considered clinically needed for any reason, including but not limited to: (i) Systolic arterial pressure lower than 90 mmHg for more than 3 min not responding to fluids and/or vasoactive drugs; (ii) New arrhythmias not responding to the treatment suggested by the Advanced Cardiac Life Support Guidelines; (iii) Need for a dosage of vasoactive drugs at the highest level tolerated, upon the attending physician's evaluation; (iv) Need of massive transfusion to maintain hematocrit > 21% (hemoglobin > 7 mg/dl); (v) Life-threatening surgical complication. The welfare of the patient will always be prioritized over their participation in the study. In such circumstances, the interventions will be stopped as appropriate.

V. <u>Risk of muscle weakness due to inadequate muscle paralysis management.</u> Our intervention bundle has been designed to reduce residual muscle weakness after surgery by protocolizing muscle paralysis administration and reversal. Thus, our

intervention patients should have a lower risk of muscle weakness than patients receiving standard of care. Muscle weakness can contribute to atelectasis, poor oxygenation, swallowing problems, aspiration of saliva or gastric contents, coughing, and pneumonia. If during our study visits we suspect this problem in any study patient, we will record it and notify the patient's primary care team. Anesthesiologists are experienced at reducing or responding to these risks that sometimes occur during usual care for surgery.

VI. Risk of pain and lightheadedness from incentive spirometry and early ambulation after surgery: Pain will be assessed before starting incentive spirometry exercises and/or ambulation, and these interventions may be delayed until pain is better controlled. Should a participant complain about pain during these postoperative interventions to the research coordinator, the interventions will be interrupted, and the research coordinator will instruct the participant to inform his /her care team about the pain and inability to perform incentive spirometry and/or ambulation. Pain management through oral, intravenous or epidural anesthesia composes the standard of care in these cases as are orders for additional doses as needed in case of discomfort. If incentive spirometry is done repeatedly too fast, or if standing up is performed too quickly, they can cause a slight temporary lightheadedness. The research coordinator will instruct the patient to do the incentive spirometry and ambulation more slowly and/or pause until the lightheadedness resolves. These two interventions are considered standard of care, as are the precautions to minimize their risks. In any case, patient safety will be prioritized. The alternative of not performing or delaying those interventions can contribute to insufficient lung expansion, low oxygenation, and increased risk of pneumonia after surgery.

VII. Discomfort and hematoma due to blood sampling: In order to minimize patient discomfort, we will attempt to synchronize routine blood test samples for clinical care with the collection of blood samples for the study whenever possible. Local pressure will be applied to minimize the risk of hematoma. Only experienced personnel in venipuncture will attempt to obtain these blood samples.

VIII. Risk to patient privacy: Patient information will be de-identified and assigned a study ID that will be used for all data records and blood samples. A list linking patient names to their study ID will be kept separate from the data in secure storage. Deidentified data, after review by the NHLBI BioLINCC data repository staff, will be available through the NHLBI BioLINCC biorepository. The plan to redact or convert all PHI data (e.g. date of hospitalization will be converted to days after randomization; rare events will be grouped to minimize the risk of participant identification; site will not be identifiable from published or internal data) will be reviewed and approved by the NHLBI BioLINCC prior to being implemented. Patients will be allowed to opt-out the biorepository of blood samples if they do not desire to participate in the availability of their blood samples for future research on pulmonary conditions or other research. All individually identifiable information (signed consent forms, data collection sheets) will be stored in a secure, locked, location in the site PI's office.

Passwordprotected computer databases will be used with limited access to the study data. An electronic data capture (EDC) system at the SDCC will be used to store all study data. Participants will be identified only by study ID also in this data system. This system, which is both HIPAA and 21CFR11 compliant, is secured from unauthorized use within the Partners IT infrastructure used to project the Massachusetts General Hospital's own hospital data.

IX. Risk of anxiety: We will minimize the risk of anxiety by assuring patients that: the study is based on sound medical reasoning and data, the study is entirely voluntary, they may withdraw and have usual care at any time, their doctors have the last say in the manner their care will be provided, and that their decision of whether or not to participate will in no way affect their care.

X. Risk of distress: We will minimize the risk of distress by reminding participants that they may skip any question if they would prefer not to answer.

If any **incidental findings** are discovered from participants' interactions with the research team, they will be communicated to the patient's primary medical team as appropriate for further handling.

VIII. POTENTIAL BENEFITS

In this study, we will determine whether an anesthesia-centered bundle involving individualization of PEEP level during abdominal surgery, optimized neuromuscular blockade administration and reversal, and supervision of incentive spirometry/early mobilization after surgery after preoperative patient education will reduce the occurrence of pulmonary complications after surgery. Each one of these interventions have been suggested in our pilot studies and in previous publications to reduce the likelihood of postoperative pulmonary complications. Accordingly, we presume that patients receiving the intervention could benefit from it by having a reduced incidence and severity of postoperative pulmonary complications.

Because patients with a large range of intraoperative surgical requirements and characteristics related to restriction of lung expansion as well as anthropometric characteristics undergo abdominal surgery, use of fixed PEEP values is not likely to be an optimal strategy to achieve a protective ventilatory strategy in clinical practice. For instance, the PEEP level for optimal lung recruitment in a thin small female patient is expected to be substantially different from that required in a large muscular male patient. Use of a fixed large PEEP in a higher PEEP interventional arm could lead to lung overexpansion in some cases, and insufficient expansion in others, ultimately increasing the risk for ventilator associated lung injury. Similarly, in the same patient the respiratory conditions change rapidly during surgery, and the optimum PEEP during periods with the surgical retractors constraining the lung expansion, for example, will likely be very different than periods without them. Accordingly, we presume that enrolled subjects who receive individualization of PEEP settings to achieve optimal individual ventilation would have optimal intraoperative mechanical ventilation during abdominal surgery and their risk of experiencing pulmonary complications after their surgery may

be lower compared to the usual care group. This rationale is also true for other efforts in our bundle to optimize lung expansion during and after surgery, including by avoiding muscle weakness after surgery and encouraging early mobilization and incentive spirometry exercises. The risks to subjects related to our intervention bundle include episodes of lower blood pressure that may happen during recruitment maneuvers and PEEP management. This potential risk, and others, is also present during usual care with mechanical ventilation for surgery, and anesthesiologists are particularly experienced at reducing and managing intraoperative low blood pressure and other possible complications. We have established specific processes to minimize these otherwise standard risks for any patient undergoing surgery and, thus, we believe our study-specific risks are reasonable in relation to the anticipated benefits to research participants and others.

The benefit to future patients and the health care system can also be important. Indeed, PPCs affect more than a million patients every year in the US, with 46,200 additional deaths, and 4.8 million additional days of hospitalization. Abdominal surgery is associated with the largest absolute number of PPCs. Respiratory complications have been reported as the costliest major postoperative complication. US estimates suggest that PPCs add $717 to the average cost of each elective surgery, 92,200 additional intensive care unit (ICU) admissions, 584,300 additional ICU days, and $3.42 billion in additional costs[18]. There is a remarkable paucity of high-level data in this field. If we establish that our bundle is relevant to postoperative pulmonary complications, this would provide evidence to modify clinical practice towards use of specific protective methods for intraoperative ventilatory settings, neuromuscular blockade administration and reversal, and early mobilization and incentive spirometry after abdominal surgery. Future patients would benefit from significant risk reduction in postoperative pulmonary complications and related morbidity and mortality. The health care system could also benefit substantially with reduction in costs of postoperative care.

IX. MONITORING AND QUALITY ASSURANCE

This study is implemented with a Data Safety Monitoring Board established by the NIH/NHLBI and a Medical Monitor. The SDCC will perform checks on accuracy and completeness of case report forms in StudyTRAX and other electronic data capture systems.

The SDCC will generate performance reports for individual sites monthly. The CCC will approach sites with performance issues to resolve them in a timely manner.

Because no new medication, new medical interventions, or new device is used in this study, except for the risks under the standard medical procedures, no additional adverse events are expected. The study design implies a high degree of individual attention to each patient during the performance of the study. Consequently, safety is evaluated in a continuous basis during execution of the study. The principal investigator

will be responsible to review safety information, communicate with the IRB and other members of the research team, and alter or stop the study.

The DSMB has been convened by the NIH-NHLBI for oversight of this trial. The principal investigator is responsible for submitting all communications received from the DSMB to the IRB.

At least one physician investigator will be immediately available throughout the entire study. Adverse events will be discussed with all research staff.

We will comply with all policies and requirements for data safety and monitoring specified by the Partners single IRB (sIRB), the DSMB, or NHLBI. Consistent with NHLBI reporting guidelines, PRIME-AIR investigators will notify the SDCC as soon as possible, but no later than seven calendar days after becoming aware of all SAEs, and within 15 calendar days of becoming aware of all other AEs. In addition, we propose that specific events (listed below) occurring during surgery that might be related to study procedures be reported as soon as possible, but not later than seven calendar days after the investigator becomes aware of their occurrence, whether serious or not.

AE reporting: There is a high complication rate after major abdominal surgery. Virtually any complication could increase duration of hospitalization and, thus, be defined as a SAE. For this reason, we propose to follow the approach defined on Table 7 to define the subset of AEs that needs to be reported with AE forms, AEs that will be reported as outcomes in the daily/discharge forms, or both. A **subset of AEs will have expedited reporting** to the sIRB and DSMB within 24 hours or 7 days of receipt by the SDCC, and these are described in detail below.

Table 7: AE Reporting – PRIME-AIR Study

AE Reporting Criteria	Actions
Serious	File AE Form
Unexpected	File AE Form
Targeted AE*	File AE Form<br>Report as clinical outcome in the corresponding clinical report forms
Other PRIME-AIR clinical outcomes not included above.	Report as clinical outcome in the corresponding clinical report forms
AE not included above and expected, not serious, or not a clinical outcome	Not reported

*Targeted AEs:
Acute myocardial ischemia or infarction
Cardiogenic pulmonary edema
Arrhythmia
Cardiac arrest
Stroke
GI bleeding
Anastomotic Breakdown
Paralytic ileus
Acute liver failure
Acute renal failure
Systemic inflammatory response syndrome
Surgical site infection
Urinary tract infection
Bacteremia
Sepsis
Septic shock
Infection - source uncertain
Disseminated intravascular coagulation
Postoperative hemorrhage
Thromboembolism
Pulmonary embolism
ARDS

AEs with Expedited Reporting: Only a subset of SAEs will have expedited reporting to the sIRB and the DSMB. We propose that only the following categories of events have expedited reporting (within 7 days of receipt by the SDCC):

- Any unanticipated problem (defined in the Detailed Protocol);
- Any AE that is serious, unexpected and suspected of being related (probably or possibly related) to the study procedures;
- Any occurrence of hemodynamic or respiratory instability, or pneumothorax, occurring during surgery and potentially arising from participation in the study.
- All adverse events that are serious, unexpected and occurring intraoperatively and immediately postoperatively (Day 0-1).

Definition of severe intraoperative respiratory AEs and hemodynamic instability. We define the intraoperative adverse events related to severe respiratory events (severe hypoxemia, pneumothorax) or severe hemodynamic instability as follows:

- Severe hypoxemia: SpO2 ≤92% or 2% below the participant's preoperative room air value (if that value is ≤92%) for at least 10 minutes; OR increase in FiO2 to 1.0 preceded by a SpO2 ≤95% in the previous 5 minutes (excluding in preparation for extubation); AND requiring treatment in intensity or duration

beyond the usual intraoperative requirements according to the participant's anesthesiologist.

- Pneumothorax: presence of air in the pleural cavity occurring during surgery and requiring intervention
- Hemodynamic instability: SBP>160 or <80 mmHg, MAP>120 or <50 mmHg, HR>140 or <50 bpm, or deviations by more than 20% in any of those parameters from patient's baseline for >3 minutes despite treatment or cardiac events (e.g. significant arrhythmias, ST segment changes or cardiac arrest); AND requiring treatment in intensity or duration beyond the usual intraoperative requirements according to the participant's anesthesiologist

Relatedness is determined by the site PI, recognizing that the site PI may not be blind to the treatment intervention. The reports are also assessed by an independent Medical Monitor.

Site-PIs will be instructed to report fatal AEs as soon as possible, latest within 24 h after becoming aware of the event. The sIRB, NHLBI, DSMB and CCC PIs will be notified as soon as the information is confirmed. The report to the DSMB will be blinded.

Events assessed as unrelated to participation in the study (including hemodynamic instability, respiratory deterioration, and pneumothorax that occur during surgery because of causes unrelated to participation in the study or occur after the end of surgery and anesthesia) will be reported within 15 calendar days to the SDCC.

For the expedited events listed above, the SDCC will have reporting procedures to notify, the CCC Co-PIs and CCC manager, the sIRB for the study, and NHLBI of the events listed above. Specific mechanisms for reporting these events to the sIRB for the study and NHLBI will be developed collaboratively with the sIRB and the NHLBI. Reports will be filed with the sIRB and NHLBI. All SAEs will be reported by the site PI in a blinded fashion and adjudicated by the blinded study PI for expectedness and relatedness to the study procedure.

For each expedited report, one of the CCC Co-PIs will contact the site PI and review the issue prior to the next weekly Study Management Committee meeting. Unanticipated problems and AEs that are serious, unexpected and suspected of being related (or possibly related) to the study procedures will be discussed at the next weekly Study Management Committee meeting. Any occurrence of hemodynamic instability, respiratory deterioration, or pneumothorax occurring during surgery and potentially arising from participation in the study will be discussed at the next weekly Study Management Committee meeting.

Because of the importance of these events, this topic will be a standing item on the meeting agenda after the first patient is enrolled on the study, and the SDCC notifies the

CCC and the Medical Monitor about them daily. The occurrence of hemodynamic instability, respiratory deterioration, or pneumothorax occurring during the surgery and thought to relate to the intervention bundle will lead to site or attending anesthesiologist retraining on the intervention bundle if appropriate. All sites will be trained in the consistent assessment and reporting of adverse events.

The DSMB letter resulting from its review of AEs and SAEs will be submitted to the sIRB within 7 calendar days of receipt.

X. REFERENCES

1. Lawrence VA, Hilsenbeck SG, Mulrow CD, Dhanda R, Sapp J, Page CP: Incidence and hospital stay for cardiac and pulmonary complications after abdominal surgery. J Gen Intern Med 1995; 10:671–8
2. Shander A, Fleisher LA, Barie PS, Bigatello LM, Sladen RN, Watson CB: Clinical and economic burden of postoperative pulmonary complications: patient safety summit on definition, risk-reducing interventions, and preventive strategies. Crit Care Med 2011; 39:2163–72
3. Fernandez-Perez ER, Sprung J, Afessa B, Warner DO, Vachon CM, Schroeder DR, Brown DR, Hubmayr RD, Gajic O: Intraoperative ventilator settings and acute lung injury after elective surgery: a nested case control study. Thorax 2009; 64:121–7 4. Canet J, Gallart L, Gomar C, Paluzie G, Valles J, Castillo J, Sabate S, Mazo V, Briones Z, Sanchis J, ARISCAT Group: Prediction of postoperative pulmonary complications in a population-based surgical cohort. Anesthesiology 2010; 113:1338–50
5. Lawrence VA, Cornell JE, Smetana GW, American College of Physicians: Strategies to reduce postoperative pulmonary complications after noncardiothoracic surgery: systematic review for the American College of Physicians. Ann Intern Med 2006; 144:596–608
6. Qaseem A, Snow V, Fitterman N, Hornbake ER, Lawrence VA, Smetana GW, Weiss K, Owens DK, Aronson M, Barry P, Casey DE Jr, Cross JT Jr, Fitterman N, Sherif KD, Weiss KB, Clinical Efficacy Assessment Subcommittee of the American College of Physicians: Risk assessment for and strategies to reduce perioperative pulmonary complications for patients undergoing noncardiothoracic surgery: a guideline from the American College of Physicians. Ann Intern Med 2006; 144:575–80
7. Futier E, Constantin JM, Paugam-Burtz C, Pascal J, Eurin M, Neuschwander A, Marret E, Beaussier M, Gutton C, Lefrant JY, Allaouchiche B, Verzilli D, Leone M, De Jong A, Bazin JE, Pereira B, Jaber S, IMPROVE Study Group: A trial of intraoperative low-tidal-volume ventilation in abdominal surgery. N Engl J Med 2013; 369:428–37
8. Ladha K, Vidal Melo MF, McLean DJ, Wanderer JP, Grabitz SD, Kurth T, Eikermann M: Intraoperative protective mechanical ventilation and risk of postoperative respiratory complications: hospital based registry study. BMJ 2015; 351:h3646 9. De Jong MAC, Ladha K, Vidal Melo MF, Staehr-Rye AK, Bittner EA, Kurth T, Eikermann M: Differential effects of intra-operative positive end-expiratory pressure (PEEP) on respiratory outcome in major abdominal surgery versus craniotomy. AnnSurg 2016; 264:362–9
10. Fernandez-Bustamante A, Wood CL, Tran ZV, Moine P: Intraoperative ventilation: incidence and risk factors for receiving large tidal volumes during general anesthesia. BMC Anesthesiol 2011; 11:22
11. Grosse-Sundrup M, Henneman JP, Sandberg WS, Bateman B, Villa Uribe J, Thuy Nguyen N, Ehrenfeld JM, Martinez EA, Kurth T, Eikermann M: Intermediate-acting

non-depolarizing neuromuscular blocking agents and risk of postoperative complications: prospective propensity score-matched cohort study. BMJ 2012; 345:e6329
12. Sasaki N, Meyer MJ, Malviya SA, Stanislaus AB, MacDonald T, Doran ME, Igumenshcheva A, Hoang AH, Eikermann M: Effects of neostigmine reversal of nondepolarizing neuromuscular blocking agents on postoperative respiratory outcomes: a prospective study. Anesthesiology 2014; 121:959–68
13. Acute Respiratory Distress Syndrome Network, Brower RG, Matthay MA, Morris A, Schoenfeld D, Thompson BT, Wheeler A: Ventilation with lower tidal volumes as compared with traditional tidal volumes for acute lung injury and the acute respiratory distress syndrome. N Engl J Med 2000; 342:1301–8
14. Marik PE, Flemmer M: The immune response to surgery and trauma: Implications for treatment. J Trauma Acute Care Surg 2012; 73:801–8
15. Schietroma M, Carlei F, Cappelli S, Amicucci G: Intestinal permeability and systemic endotoxemia after laparotomic or laparoscopic cholecystectomy. Ann Surg 2006; 243:359–63
16. MacFie J, Reddy BS, Gatt M, Jain PK, Sowdi R, Mitchell CJ: Bacterial translocation studied in 927 patients over 13 years. Br J Surg 2006; 93:87–93
17. Ford GT, Whitelaw WA, Rosenal TW, Cruse PJ, Guenter CA: Diaphragm function after upper abdominal surgery in humans. Am Rev Respir Dis 1983; 127:431–6
18. Tokics L, Hedenstierna G, Strandberg A, Brismar B, Lundquist H: Lung collapse and gas exchange during general anesthesia: effects of spontaneous breathing, muscle paralysis, and positive end-expiratory pressure. Anesthesiology 1987; 66:157–67
19. Duggan M, Kavanagh BP: Pulmonary atelectasis: a pathogenic perioperative entity. Anesthesiology 2005; 102:838–54
20. Melo MF, Eikermann M: Protect the Lungs during Abdominal Surgery: It May Change the Postoperative Outcome. Anesthesiology 2013; 118:1254–7
21. Altemeier WA, Matute-Bello G, Frevert CW, Kawata Y, Kajikawa O, Martin TR, Glenny RW: Mechanical ventilation with moderate tidal volumes synergistically increases lung cytokine response to systemic endotoxin. Am J Physiol Cell Mol Physiol 2004; 287:L533-42
22. Michelet P, D'Journo XB, Roch A, Doddoli C, Marin V, Papazian L, Decamps I, Bregeon F, Thomas P, Auffray JP: Protective ventilation influences systemic inflammation after esophagectomy: a randomized controlled study. Anesthesiology 2006; 105:911–9
23. PROVE Network Investigators for the Clinical Trial Network of the European Society of Anaesthesiology, Hemmes SN, Gama de Abreu M, Pelosi P, Schultz MJ: High versus low positive end-expiratory pressure during general anaesthesia for open abdominal surgery (PROVHILO trial): a multicentre randomised controlled trial. Lancet 2014; 384:495–503
24. Guldner A, Kiss T, Serpa Neto A, Hemmes SN, Canet J, Spieth PM, Rocco PR, Schultz MJ, Pelosi P, Gama de Abreu M: Intraoperative protective mechanical ventilation for prevention of postoperative pulmonary complications: a comprehensive review of the role of tidal volume, positive end-expiratory pressure, and lung recruitment maneuvers. Anesthesiology 2015; 123:692–713

25. Murphy GS, Szokol JW, Avram MJ, Greenberg SB, Shear TD, Vender JS, Parikh KN, Patel SS, Patel A: Residual Neuromuscular Block in the Elderly: Incidence and Clinical Implications. Anesthesiology 2015; 123:1322–36
26. Fernandez-Bustamante A, Frendl G, Sprung J, Kor DJ, Subramaniam B, Martinez Ruiz R, Lee JW, Henderson WG, Moss A, Mehdiratta N, Colwell MM, Bartels K, Kolodzie K, Giquel J, Vidal Melo MF: Postoperative Pulmonary Complications, Early Mortality, and Hospital Stay Following Noncardiothoracic Surgery: A Multicenter Study by the Perioperative Research Network Investigators. JAMA Surg 2017; 152:157–66
27. Calfee CS, Janz DR, Bernard GR, May AK, Kangelaris KN, Matthay MA, Ware LB, the NIH NHLBI ARDS Network: Distinct Molecular Phenotypes of Direct vs Indirect ARDS in Single-Center and Multicenter Studies. Chest 2015; 147:1539–48
28. Ranieri VM, Suter PM, Tortorella C, De Tullio R, Dayer JM, Brienza A, Bruno F, Slutsky AS: Effect of mechanical ventilation on inflammatory mediators in patients with acute respiratory distress syndrome: a randomized controlled trial. JAMA J Am Med Assoc 1999; 282:54–61
29. Fernandez-Bustamante A, Klawitter J, Repine JE, Agazio A, Janocha AJ, Shah C, Moss M, Douglas IS, Tran ZV, Erzurum SC, Christians U, Seres T: Early effect of tidal volume on lung injury biomarkers in surgical patients with healthy lungs. Anesthesiology 2014; 121:469–81
30. Serpa Neto A, Campos PPZA, Hemmes SN, Bos LD, Bluth T, Ferner M, Guldner A, Hollmann MW, India I, Kiss T, Laufenberg-Feldmann R, Sprung J, Sulemanji D, Unzueta C, Vidal Melo MF, Weingarten TN, Tuip-de Boer AM, Pelosi P, Gama de Abreu M, Schultz MJ: Kinetics of Plasma Biomarkers of Inflammation and Lung Injury in Surgical Patients with or without Postoperative Pulmonary Complications. Eur J Anaesthesiol 2017; 34:229–38
31. Parsons PE, Eisner MD, Thompson BT, Matthay MA, Ancukiewicz M, Bernard GR, Wheeler AP, NHLBI Acute Respiratory Distress Syndrome Clinical Trials Network: Lower tidal volume ventilation and plasma cytokine markers of inflammation in patients with acute lung injury. Crit Care Med 2005; 33:1–6
32. Slutsky AS, Ranieri VM: Ventilator-induced lung injury. N Engl J Med 2013; 369:2126–36
33. Fernandez-Bustamante A, Frendl G, Sprung J, Kor DJ, Subramanian B, Martinez-Ruiz R, Lee JW, Henderson WG, Moss A, Mehdiratta N, Colwell MM, Bartels K, Kolodzie K, Giquel J, Vidal Melo MF: Multicenter prospective assessment of postoperative pulmonary complications, early mortality and hospital stay by the Perioperative Research Network (PRN) investigators. JAMA Surg 2016:Accepted for publication-Accepted for publication
34. Blum JM, Maile M, Park PK, Morris M, Jewell E, Dechert R, Rosenberg AL: A description of intraoperative ventilator management in patients with acute lung injury and the use of lung protective ventilation strategies. Anesthesiology 2011; 115:75–82
35. Kor DJ, Lingineni RK, Gajic O, Park PK, Blum JM, Hou PC, Hoth JJ, Anderson HL 3rd, Bajwa EK, Bartz RR, Adesanya A, Festic E, Gong MN, Carter RE, Talmor DS:

Predicting risk of postoperative lung injury in high-risk surgical patients: a multicenter cohort study. Anesthesiology 2014; 120:1168–81
36. Serpa Neto A, Hemmes SN, Barbas CS, Beiderlinden M, Fernandez-Bustamante A, Futier E, Hollmann MW, Jaber S, Kozian A, Licker M, Lin WQ, Moine P, Scavonetto F, Schilling T, Selmo G, Severgnini P, Sprung J, Treschan T, Unzueta C, Weingarten TN, Wolthuis EK, Wrigge H, Gama de Abreu M, Pelosi P, Schultz MJ, PROVE Network investigators: Incidence of mortality and morbidity related to postoperative lung injury in patients who have undergone abdominal or thoracic surgery: a systematic review and meta-analysis. LancetRespiratory Med 2014; 2:1007–15
37. Coger K, Frendl G, Sprung J, Kor DJ, Subramaniam B, Martinez Ruiz R, Lee JW, Henderson WG, Moss A, Mehdiratta N, Colwell MM, Bartels K, Kolodzie K, Giquel J, Vidal Melo MF, Fernandez-Bustamante A: Postoperative Pulmonary Complications Not Increased With Combined Regional + General Anesthesia Compared To General Anesthesia Alone: A Sub-Analysis Of The Perioperative Research Network Study 2017
38. Squadrone V, Coha M, Cerutti E, Schellino MM, Biolino P, Occella P, Belloni G, Vilianis G, Fiore G, Cavallo F, Ranieri VM, Piedmont Intensive Care Units Network (PICUN): Continuous positive airway pressure for treatment of postoperative hypoxemia: a randomized controlled trial. Jama 2005; 293:589–95
39. McLean DJ, Diaz-Gil D, Farhan HN, Ladha KS, Kurth T, Eikermann M: Dosedependent Association between Intermediate-acting Neuromuscular-blocking Agents and Postoperative Respiratory Complications. Anesthesiology 2015; 122:1201–13
40. Pasquina P, Tramer MR, Granier JM, Walder B: Respiratory physiotherapy to prevent pulmonary complications after abdominal surgery: a systematic review. Chest 2006; 130:1887–99
41. Duggan M, Kavanagh BP: Perioperative modifications of respiratory function. Best Pract Res Anaesthesiol 2010; 24:145–55
42. Tusman G, Bohm SH, Warner DO, Sprung J: Atelectasis and perioperative pulmonary complications in high-risk patients. Curr Opin Anaesthesiol 2012; 25:1–10
43. Wozniak DR, Lasserson TJ, Smith I: Educational, supportive and behavioural interventions to improve usage of continuous positive airway pressure machines in adults with obstructive sleep apnoea. Cochrane Database Syst Rev 2014; (1):CD007736. doi:CD007736
44. Lai AYK, Fong DYT, Lam JCM, Weaver TE, Ip MSM: The efficacy of a brief motivational enhancement education program on CPAP adherence in OSA: a randomized controlled trial. Chest 2014; 146:600–10
45. Narendra DK, Hess DR, Sessler CN, Belete HM, Guntupalli KK, Khusid F, Carpati CM, Astiz ME, Raoof S: Update in Management of Severe Hypoxemic Respiratory Failure. Chest 2017; 152:867–79
46. Neto AS, Hemmes SN, Barbas CS, Beiderlinden M, Fernandez-Bustamante A, Futier E, Gajic O, El-Tahan MR, Ghamdi AA, Gunay E, Jaber S, Kokulu S, Kozian A, Licker M, Lin WQ, Maslow AD, Memtsoudis SG, Reis Miranda D, Moine P, Ng T,

Paparella D, Ranieri VM, Scavonetto F, Schilling T, Selmo G, Severgnini P, Sprung J, Sundar S, Talmor D, Treschan T, et al.: Association between driving pressure and development of postoperative pulmonary complications in patients undergoing mechanical ventilation for general anaesthesia: a meta-analysis of individual patient data. LancetRespiratory Med 2016; 4:272–80

47. Amato MB, Meade MO, Slutsky AS, Brochard L, Costa EL, Schoenfeld DA, Stewart TE, Briel M, Talmor D, Mercat A, Richard JC, Carvalho CR, Brower RG: Driving pressure and survival in the acute respiratory distress syndrome. N Engl J Med 2015; 372:747–55
48. Writing Group for the Alveolar Recruitment for Acute Respiratory Distress Syndrome Trial (ART) Investigators, Cavalcanti AB, Suzumura EA, Laranjeira LN, Paisani DM, Damiani LP, Guimaraes HP, Romano ER, Regenga MM, Taniguchi LNT, Teixeira C, Pinheiro de Oliveira R, Machado FR, Diaz-Quijano FA, Filho MSA, Maia IS, Caser EB, Filho WO, Borges MC, Martins PA, Matsui M, Ospina-Tascon GA, Giancursi TS, Giraldo-Ramirez ND, Vieira SRR, Assef MDGPL, Hasan MS, Szczeklik W, Rios F, Amato MBP, et al.: Effect of Lung Recruitment and Titrated Positive End-Expiratory Pressure (PEEP) vs Low PEEP on Mortality in Patients With Acute Respiratory Distress Syndrome: A Randomized Clinical Trial. Jama 2017; 318:1335–45
49. Ferrando C, Soro M, Unzueta C, Suarez-Sipmann F, Canet J, Librero J, Pozo N, Peiro S, Llombart A, Leon I, India I, Aldecoa C: Individualised perioperative openlung approach versus standard protective ventilation in abdominal surgery (iPROVE): a randomised controlled trial. Lancet RespirMed 2018:Published Online January 19, 2018-Published Online January 19, 2018
50. Ferrando C, Soro M, Canet J, Unzueta MC, Suarez F, Librero J, Peiro S, Llombart A, Delgado C, Leon I, Rovira L, Ramasco F, Granell M, Aldecoa C, Diaz O, Balust J, Garutti I, Matta M de la, Pensado A, Gonzalez R, Duran ME, Gallego L, Del Valle SG, Redondo FJ, Diaz P, Pestana D, Rodriguez A, Aguirre J, Garcia JM, Garcia J, et al.: Rationale and study design for an individualized perioperative open lung ventilatory strategy (iPROVE): study protocol for a randomized controlled trial. Trials 2015; 16:193
51. Kroenke K, Lawrence VA, Theroux JF, Tuley MR: Operative risk in patients with severe obstructive pulmonary disease. Arch Intern Med 1992; 152:967–71
52. Hulzebos EH, Helders PJ, Favie NJ, De Bie RA, Brutel de la Riviere A, Van Meeteren NL: Preoperative intensive inspiratory muscle training to prevent postoperative pulmonary complications in high-risk patients undergoing CABG surgery: a randomized clinical trial. Jama 2006; 296:1851–7
53. Rackley CR, Levitt JE, Zhuo H, Matthay MA, Calfee CS: Clinical evidence of early acute lung injury often precedes the diagnosis of ALI. J Intensive Care Med 2013; 28:241–6
54. Mazo V, Sabate S, Canet J, Gallart L, Abreu MG de, Belda J, Langeron O, Hoeft A, Pelosi P: Prospective external validation of a predictive score for postoperative pulmonary complications. Anesthesiology 2014; 121:219–31
55. McAlister FA, Bertsch K, Man J, Bradley J, Jacka M: Incidence of and risk factors for pulmonary complications after nonthoracic surgery. Am J Respir Crit Care Med 2005; 171:514–7

56. Manku K, Bacchetti P, Leung JM: Prognostic significance of postoperative inhospital complications in elderly patients. I. Long-term survival. Anesth Analg 2003; 96:583–9
57. Visser BC, Keegan H, Martin M, Wren SM: Death after colectomy: it's later than we think. Arch Surg Chic Ill 1960 2009; 144:1021–7
58. Fleisher LA, Linde-Zwirble WT: Incidence, outcome, and attributable resource use associated with pulmonary and cardiac complications after major small and large bowel procedures. Perioper Med Lond Engl 2014; 3:7
59. Dindo D, Demartines N, Clavien PA: Classification of surgical complications: a new proposal with evaluation in a cohort of 6336 patients and results of a survey. Ann Surg 2004; 240:205–13
60. Singer M, Deutschman CS, Seymour CW, Shankar-Hari M, Annane D, Bauer M, Bellomo R, Bernard GR, Chiche JD, Coopersmith CM, Hotchkiss RS, Levy MM, Marshall JC, Martin GS, Opal SM, Rubenfeld GD, Poll T van der, Vincent JL, Angus DC: The Third International Consensus Definitions for Sepsis and Septic Shock (Sepsis-3). Jama 2016; 315:801–10
61. Jammer I, Wickboldt N, Sander M, Smith A, Schultz MJ, Pelosi P, Leva B, Rhodes A, Hoeft A, Walder B, Chew MS, Pearse RM, European Society of Anaesthesiology (ESA) and the European Society of Intensive Care Medicine (ESICM), European Society of Anaesthesiology, European Society of Intensive Care Medicine: Standards for definitions and use of outcome measures for clinical effectiveness research in perioperative medicine: European Perioperative Clinical Outcome (EPCO) definitions: a statement from the ESA-ESICM joint taskforce on perioperative outcome measures. Eur J Anaesthesiol 2015; 32:88–105
62. Davies SJ, Francis J, Dilley J, Wilson RJ, Howell SJ, Allgar V: Measuring outcomes after major abdominal surgery during hospitalization: reliability and validity of the Postoperative Morbidity Survey. Perioper Med Lond Engl 2013; 2:1
63. Bennett-Guerrero E, Welsby I, Dunn TJ, Young LR, Wahl TA, Diers TL, Phillips-Bute BG, Newman MF, Mythen MG: The use of a postoperative morbidity survey to evaluate patients with prolonged hospitalization after routine, moderate-risk, elective surgery. Anesth Analg 1999; 89:514–9
64. Hess DR, Kondili D, Burns E, Bittner EA, Schmidt UH: A 5-year observational study of lung-protective ventilation in the operating room: A single-center experience. J Crit Care 2013; 28:533.e9-15
65. Fernandez-Bustamante A, Sprung J, Bartels K, Weingarten T, Kosour C, Vidal Melo MF: Titrated PEEP To Optimize Lung Mechanics During Abdominal Surgery Exceeds 2 cmH2O and Reduces Driving Pressures, Anesthesiology Annual Metting. 2018
66. Hess DR: Recruitment Maneuvers and PEEP Titration. Respir Care 2015; 60:1688–704
67. Brower RG, Lanken PN, MacIntyre N, Matthay MA, Morris A, Ancukiewicz M, Schoenfeld D, Thompson BT, National Heart L and Blood Institute ARDS Clinical Trials Network: Higher versus lower positive end-expiratory pressures in patients with the acute respiratory distress syndrome. N Engl J Med 2004; 351:327–36

68. Jacobs I, Nadkarni V, Bahr J, Berg RA, Billi JE, Bossaert L, Cassan P, Coovadia A, D'Este K, Finn J, Halperin H, Handley A, Herlitz J, Hickey R, Idris A, Kloeck W, Larkin GL, Mancini ME, Mason P, Mears G, Monsieurs K, Montgomery W, Morley P, Nichol G, Nolan J, Okada K, Perlman J, Shuster M, Steen PA, Sterz F, et al.: Cardiac arrest and cardiopulmonary resuscitation outcome reports: update and simplification of the Utstein templates for resuscitation registries: a statement for healthcare professionals from a task force of the International Liaison Committee on Resuscitation (American Heart Association, European Resuscitation Council, Australian Resuscitation Council, New Zealand Resuscitation Council, Heart and Stroke Foundation of Canada, InterAmerican Heart Foundation, Resuscitation Councils of Southern Africa). Circulation 2004; 110:3385–97
69. Peel AL, Taylor EW: Proposed definitions for the audit of postoperative infection: a discussion paper. Surgical Infection Study Group. Ann R Coll Surg Engl 1991; 73:385–8
70. Bree SHW van, Bemelman WA, Hollmann MW, Zwinderman AH, Matteoli G, El Temna S, The FO, Vlug MS, Bennink RJ, Boeckxstaens GEE: Identification of clinical outcome measures for recovery of gastrointestinal motility in postoperative ileus. Ann Surg 2014; 259:708–14
71. Khwaja A: KDIGO clinical practice guidelines for acute kidney injury. Nephron Clin Pract 2012; 120:c179-184
72. Horan TC, Andrus M, Dudeck MA: CDC/NHSN surveillance definition of health careassociated infection and criteria for specific types of infections in the acute care setting. Am J Infect Control 2008; 36:309–32
73. Taylor FB, Toh CH, Hoots WK, Wada H, Levi M, Scientific Subcommittee on Disseminated Intravascular Coagulation (DIC) of the International Society on Thrombosis and Haemostasis (ISTH): Towards definition, clinical and laboratory criteria, and a scoring system for disseminated intravascular coagulation. Thromb Haemost 2001; 86:1327–30
74. Boral BM, Williams DJ, Boral LI: Disseminated Intravascular Coagulation. Am J Clin Pathol 2016; 146:670–80

Appendix I – Definitions of Outcomes

List and definitions of secondary and exploratory outcomes:

- All-cause mortality at 7, 30 and 90 days: mortality for any cause within 7, 30, and 90 days after the day of surgery;

- Grade 3 and 4 PPCs at 30 and 90 days: individual grade 3 and 4 PPCs within 30 and 90 days after the day of surgery;

- Presence of each of the individual components of the list of pulmonary complications in the primary endpoint (Table 5) within 7, 30 and 90 days after the day of surgery;

- Rate of intraoperative adverse events (hypoxemia, hypotension during lung recruitment, need for vasoactive medications and volume replacement, muscle weakness. This will be assessed as TOF<0.9 after extubation, or, in the absence of quantitative assessment, clinical assessments such as inability to generate a tidal volume above 4ml/kg of predicted body weight, PBW, at time of extubation or to maintain sustained hand grip or 5-s head lift, presence of diplopia or ventilatory failure after extubation);

- Rate of major extrapulmonary complications, defined based on existing diagnosis in the medical chart following usual accepted definitions[61] unless otherwise specified:

  - Cardiovascular:
    - myocardial ischemia or infarction: Increase in serum cardiac biomarker values (preferably cardiac troponin) with at least one value above the 99th percentile upper reference limit and at least one of the following criteria[65]:
      - Symptoms of myocardial ischemia;
      - New ischemic ECG changes;
      - Development of pathological Q waves;
      - Imaging evidence of new loss of viable myocardium or new regional wall motion abnormality in a pattern consistent with an ischemic etiology;
      - Identification of a coronary thrombus by angiography or autopsy.
    - cardiogenic pulmonary edema: Cardiogenic pulmonary edema is defined as evidence of fluid accumulation in the alveoli due to poor cardiac function.

- arrhythmia: electrocardiograph (ECG) evidence of cardiac rhythm disturbance.
- cardiac arrest: The International Liaison Committee on Resuscitation defines cardiac arrest as the cessation of cardiac mechanical activity, as confirmed by the absence of signs of circulation[68].
- stroke: defined as an embolic, thrombotic or hemorrhagic cerebral event with persistent residual motor, sensory or cognitive dysfunction (e.g. hemiplegia, hemiparesis, aphasia, sensory deficit, impaired memory).

- Gastrointestinal:
  - bleeding: Gastrointestinal bleed is defined as unambiguous clinical or endoscopic evidence of blood in the gastrointestinal tract. Upper gastrointestinal bleeding (or hemorrhage) is that originating proximal to the ligament of Treitz, in practice from the esophagus, stomach and duodenum. Lower gastrointestinal bleeding is that originating from the small bowel or colon.
  - anastomotic breakdown: Leak of luminal contents from a surgical connection between two hollow viscera. The luminal contents may emerge either through the wound or at the drain site, or they may collect near the anastomosis, causing fever, abscess, septicemia, metabolic disturbance and/or multiple organ failure. The escape of luminal contents from the site of the anastomosis into an adjacent localized area, detected by imaging, in the absence of clinical symptoms and signs should be recorded as a subclinical leak[69].
  - paralytic ileus; Failure to tolerate solid food or defecate for three or more days after surgery[70].

- Liver Disease:
  Liver failure: serum bilirubin level>34 μmol/L with elevation of the transaminase and lactic dehydrogenase levels above twice normal values.

- Acute renal failure: Stage 1 or higher Kidney Disease Improving Global Outcomes (KDIGO) guidelines[71] (Table A1).

Table A1 Kidney Disease Improving Global Outcomes Guidelines (KIDGO)

**Stage**	**Serum Creatinine**	**Urine Output**
1	1.5–1.9 times baseline value within 7 days<br>or<br>≥27mmol/L (≥0.3 mg/dL) increase within 48 h	≤0.5 mL/kg/h for 6–12 h
2	2.0–2.9 times baseline value within 7 days	≤0.5 mL/kg/h for 12 h

3	3.0 times baseline value within 7 days<br>or<br>Increase in serum creatinine to ≥354 mmol/L (≥4.0 mg/dL with an acute rise of >44 mmol/L (0.5 mg/dL)<br>or<br>Initiation of renal replacement therapy	≤0.3 mL/kg/h for 24 h<br>or<br>Anuria for 12 h

- Systemic inflammatory response syndrome (SIRS): Defined by the presence of two or more of the following[60]
  - Temperature >38°C or <36°C
  - Heart rate >90/min
  - Respiratory rate >20/min or $PaCO_2$ <32 mmHg (4.3 kPa)
  - White blood cell count >12 000/mm^3 or <4000/mm^3

- Infections
  - Source uncertain: The CDC defines infection, source uncertain as one where there is strong clinical suspicion of infection but the source has not been confirmed because clinical information suggests more than one possible site, meeting two or more of the following criteria[72] core temperature <36°C or >38°C; white cell count >12 x 10^9/L or <4 x 10^9/L; respiratory rate >20 breaths/minute or PaCO2 <4.7 kPa (35mmHg); pulse rate >90 beats/minute
  - Bacteremia (bloodstream infection): The CDC defines laboratory confirmed bloodstream infection as one which meets at least one of the following criteria[72] which should not be related to infection at another site:
    1) Patient has a recognized pathogen cultured from one or more blood cultures and the organism cultured from blood is not related to an infection at another site.
    2) Patient has at least one of the following signs or symptoms: fever > 38°C, chills or hypotension, and at least one of the following:
       a. Common skin contaminant cultured from two or more blood cultures drawn on separate occasions
       b. Common skin contaminant cultured from at least one blood culture from a patient with an intravascular line, and the physician institutes appropriate antimicrobial therapy
       c. Positive blood antigen test.
  - Sepsis: defined following the Sepsis-3 criteria[60]. Sepsis is a life-threatening organ dysfunction caused by a dysregulated host response to infection. Organ dysfunction can be identified as an acute change in total SOFA score ≥2 points consequent to the

infection. The baseline SOFA score can be assumed to be zero in patients not known to have preexisting organ dysfunction. The SOFA score is included in the table below

Table 1. Sequential [Sepsis-Related] Organ Failure Assessment Score[a]

System	Score 0	1	2	3	4
Respiration					
Pa_{O_2}/F_{IO_2}, mm Hg (kPa)	≥400 (53.3)	<400 (53.3)	<300 (40)	<200 (26.7) with respiratory support	<100 (13.3) with respiratory support
Coagulation					
Platelets, $\times 10^3/\mu L$	≥150	<150	<100	<50	<20
Liver					
Bilirubin, mg/dL (μmol/L)	<1.2 (20)	1.2-1.9 (20-32)	2.0-5.9 (33-101)	6.0-11.9 (102-204)	>12.0 (204)
Cardiovascular	MAP ≥70 mm Hg	MAP <70 mm Hg	Dopamine <5 or dobutamine (any dose)[b]	Dopamine 5.1-15 or epinephrine ≤0.1 or norepinephrine ≤0.1[b]	Dopamine >15 or epinephrine >0.1 or norepinephrine >0.1[b]
Central nervous system					
Glasgow Coma Scale score[c]	15	13-14	10-12	6-9	<6
Renal					
Creatinine, mg/dL (μmol/L)	<1.2 (110)	1.2-1.9 (110-170)	2.0-3.4 (171-299)	3.5-4.9 (300-440)	>5.0 (440)
Urine output, mL/d				<500	<200

Abbreviations: F_{IO_2}, fraction of inspired oxygen; MAP, mean arterial pressure; Pa_{O_2}, partial pressure of oxygen.

[a] Adapted from Vincent et al.[27]

[b] Catecholamine doses are given as μg/kg/min for at least 1 hour.

[c] Glasgow Coma Scale scores range from 3-15; higher score indicates better neurological function.

- <u>Surgical site infection:</u> defined as one which meets the following criteria[72].
  1) Infection occurs within 30 days after surgery and
  2) Involves skin and subcutaneous tissue of the incision, or deep soft tissues (e.g. fascial and muscle layers) of the incision, or the infection appears to be related to the surgical procedure and involves any part of the body, excluding the skin incision, fascia or muscle layers opened or manipulated during the operative procedure.
  3) The patient has at least one of the following:
     a. purulent drainage from the superficial/deep incision or from a drain that is placed through a stab wound into the organ/space
     b. organisms isolated from an aseptically obtained culture of fluid or tissue from the superficial incision
     c. at least one of the following symptoms or signs of infection:
        i. pain or tenderness, localized swelling, redness or heat, and superficial incision is deliberately opened by surgeon and is culture positive or not cultured. A culture negative finding does not meet this criterion.
        ii. a deep incision spontaneously dehisces or is deliberately opened by a surgeon and is culture-positive or not cultured when the patient has at least one of the following symptoms or signs: fever (> 38°C), or

localized pain or tenderness. A culture-negative finding does not meet this criterion.

iii. an abscess or other evidence of infection involving the deep incision is found on direct examination, during surgery, or by histopathological or radiological examination.

iv. an abscess or other evidence of infection involving the organ/space that is found on direct examination, during reoperation or by histopathological or radiological examination.

d. diagnosis of a surgical site infection by a surgeon or attending physician.

- o <u>Urinary tract infection:</u> A simplified version of the CDC recommendations defines a urinary tract infection as follows: a positive urine culture of $\geq 10^5$ colony forming units per mL with no more than two species of micro-organisms, and with at least one of the following symptoms or signs: fever (> 38°C), urgency, frequency, dysuria, suprapubic tenderness, costovertebral angle pain or tenderness with no other recognized cause[72].

- Coagulation
  - o <u>Disseminated Intravascular Coagulation (DIC):</u> defined following the 2001 definition by the International Society on Thrombosis and Hemostasis (ISTH): “DIC is an acquired syndrome characterized by the intravascular activation of coagulation without a specific localization and arising from different causes. It can originate from and cause damage to the microvasculature, which if sufficiently severe, can produce organ dysfunction”[74]. It will be diagnosed following the modified algorithm currently used and described[75]:

Acute DIC Algorithm Proposed by the International Society on Thrombosis and Haemostasis[a]

Algorithm
1. Presence of an underlying disorder known to be associated with DIC?<br>If yes: proceed. If no: do not use this algorithm
2. Global coagulation results:
a. Platelet count (>100,000/μL = 0, <100,000/μL = 1, <50,000/μL = 2)
b. Fibrin degradation products such as D-dimer (no increase = 0, moderate increase = 2, strong increase = 3)
c. Prolonged prothrombin time (<3 seconds = 0, >3 seconds = 1, >6 seconds = 2)
d. Fibrinogen level (>1.0 g/L = 0; <1.0 g/L = 1)

DIC, disseminated intravascular coagulation.
[a]Interpretation of algorithm: A score of 5 or higher is compatible with acute DIC. The algorithm can be repeated on occasion if acute DIC remains a consideration and the laboratory values change. Modified from Taylor et al.[6]

- Postoperative hemorrhage: defined as blood loss within 72 h after the start of surgery which would normally result in transfusion of blood.
- Thromboembolism: A new blood clot or thrombus or embolus reducing blood circulation within the venous or arterial systems with the exception of the pulmonary venous system.
- Pulmonary embolism: A new blood clot or thrombus or embolus reducing blood circulation within the venous pulmonary system.

- Other (describe):

– Length of stay in the post-anesthesia care unit

– Length of postoperative oxygen therapy/other respiratory support

– Unexpected admission to ICU

– Length of ICU stay

– Length of hospital stay

– Hospital readmission(s) after initial discharge

– Patient-Reported Outcomes Measurements (PROMIS®) short forms (Appendix IV) addressing: Dyspnea Characteristics, Severity and Functional Limitations; and Fatigue, at days 7, 30 and 90 after the day of surgery, compared to before surgery.

– Plasma concentrations (raw and ratios compared to individual’s levels before surgery) of selected biomarkers of inflammatory, epithelial and endothelial injury.

Appendix II - Written educational material for the intervention group

Patient Education for the PRIME-AIR study

Our **goal** with this study is to reduce respiratory complications after abdominal surgery. These complications are one of the main reasons why patients like you have a slower recovery and stay in the hospital longer. We think that keeping your lungs ‘open’ (well filled with air) may improve the amount of oxygen in your blood, reduce the risk of lung infection and other respiratory complications and lower the need for breathing treatments.

What **we** will do:

- Before surgery: We will teach you breathing exercises (incentive spirometry).
- During surgery: We will use the ventilator (the machine that helps you breathe during surgery) to keep your lungs well inflated during your surgery. We will also make sure that your muscles are not weak at the end of surgery. You will be under anesthesia for this part and not aware of it.
- After surgery: We will work with you so that you do your breathing exercises well after surgery. We will also check that you are getting out of bed and starting to walk as expected. We will check on you and your medical chart to record your respiratory symptoms, complications and treatments. We will do this several times every day after your surgery until you leave the hospital or up to 7 days after your surgery. We will follow up with you by calling you at home or visiting you at the hospital after 7, 30 and 90 **days** have passed after your surgery.

What we would like **you** to do:
Follow current recommendations for patients after surgery like yours. These include **breathing exercises (incentive spirometry) and ambulation to keep your lungs open after surgery.**

- Keep the head of your bed elevated (higher than 30°), if possible.
- Do your breathing exercises (**incentive spirometry**). For these:
  - Keep the incentive spirometer within reach.
  - Sit up as straight as possible and cough to clear your lungs if needed.
    - Support your incision with a pillow or by hugging your belly with your arm
  - Place your lips around the mouthpiece and hold the incentive spirometer upright.
  - Breathe in **slowly** and as deeply as possible by raising the ball or the piston/plate in the chamber of your device to the set target; **hold your breath for at least 5 seconds once your lungs are full.** If your incentive spirometer has a floating ball, your goal is to keep that ball in the middle of the chamber while you breathe in.
  - Exhale slowly and repeat.
  - Do 10 incentive spirometry breaths every hour while awake for at least 10 hours per day. This will add up to **at least 100 deep breaths/day**.
- As soon as you can after surgery, get out of bed and walk (**ambulation**)
  - Check with your nurses and doctors if it is safe to move, and get assistance.

- Please ask your nurse or doctor for pain medication if you need it for controlling your pain.
- We hope that you can achieve these goals:
  - Day-of-surgery (**Day 0**): sit up in a chair with assistance.
  - Day after surgery (**Day 1**): sit up in a chair 3 times/day for meals; walk in room or hallway 2 times/day with assistance.
  - **Day 2**: get out of bed for all meals and for at least 6 hours during the day; walk in hallway more than 15 meters (about 50 feet) or approx. 20 steps) 3 times/day with assistance.
  - **Day 3 and on**: increase ambulation, ideally walking beyond 75 m (about 250 feet or approx. 100 steps) 3–5 times/day.

Thank you for participating in this study!

Appendix III - Maximal compliance strategy for PEEP titration

**Ventilatory Settings**	• Volume-controlled ventilation, square flow-waveform<br>• V_T=7 mL/kgPBW, adjustment to 6-8 ml/kg PBW if required<br>• RR=12 bpm titrated to $PetCO_2$=35-45 mmHg<br>• F_IO_2 ≥0.4 to SpO_2≥96%<br>• I:E ratio started at 1:2 and adjusted if necessary to the patient's physiology<br>• Inspiratory pause 20%<br>• Maximum acceptable plateau pressure 30 cmH_2O, V_T will be reduced if needed to achieve that value
**When**	• Immediately following intubation;<br>• After any intervention potentially associated with lung collapse such as application of surgical retractors, pneumoperitoneum insufflation/deflation, disconnection of the endotracheal tube, tracheal suctioning, Trendelenburg position, or if the static respiratory system compliance is reduced by ≥15%;<br>• Otherwise, hourly trials will be done counted from the last trial.
**Recruitment maneuver**	• Assure adequate muscle paralysis, volemia and hemodynamics<br>• Recruitment maneuver: This will consist of a **stepwise incremental PEEP maneuver**:<br>In volume-controlled ventilation, starting from a PEEP=0 cmH_2O, increase PEEP in steps of 5 cmH_20 until Ppl=30-32 cmH_2O cmH_2O (i.e., PEEP= 5, 10, 15 and 20 cmH_2O). Remain for at least 30 s at each step. If there is any concern for cardiopulmonary instability at the last step, this can be reduced to 2-3 cmH_2O aiming for the targeted Ppl range.<br>Address hemodynamic issues if they occur before advancing to a higher PEEP step, and reduce PEEP if needed to prioritize hemodynamic management before proceeding.<br>Recruitment maneuvers will be performed before the spontaneous breathing trial preceding extubation.<br>The maneuver will be interrupted if the patient becomes bradycardic or tachycardic (< 60 or > 140 beats per minute), develops a new arrhythmia, becomes hypotensive (systolic blood pressure < 80 mmHg) or hypoxemic (SpO_2 < 85%). Incidence of out-of-protocol change of ventilatory settings to assure SpO_2>90%, Ppl≤30 cmH_2O, or at the discretion of the patient's anesthesia providers will be recorded.
**Decremental PEEP Titration**	Performed in steps of 45-75 s each until a drop in the static Crs is observed. At this point the PEEP is set at the value which reached the recruitment range of $P_{plateau}$ =30-32 cmH_2O for 30 sec after which the chosen PEEP is set to continue ventilation. If a value lower than 20 cmH_2O was used as the maximal level of recruitment, that will be the starting PEEP for the decremental titration.

Appendix IV – PROMIS Forms

Short Form Fatigue:

PROMIS Short Form v1.0 - Fatigue - 13a - 20Feb2017
http://www.healthmeasures.net/index.php?option=com_instruments&view=measure&id=822&Itemid=992

PROMIS® Item Bank v1.0 – Fatigue – Short Form 13a (FACIT-Fatigue)

Fatigue – Short Form 13a (FACIT-Fatigue)

Please respond to each question or statement by marking one box per row.

	During the past 7 days…	Not at all	A little bit	Somewhat	Quite a bit	Very much
HI7	I feel fatigued	☐ 1	☐ 2	☐ 3	☐ 4	☐ 5
HI12	I feel weak all over	☐ 1	☐ 2	☐ 3	☐ 4	☐ 5
AN1	I feel listless ("washed out")	☐ 1	☐ 2	☐ 3	☐ 4	☐ 5
AN2	I feel tired	☐ 1	☐ 2	☐ 3	☐ 4	☐ 5
AN3	I have trouble starting things because I am tired	☐ 1	☐ 2	☐ 3	☐ 4	☐ 5
AN4	I have trouble finishing things because I am tired	☐ 1	☐ 2	☐ 3	☐ 4	☐ 5
AN5	I have energy	☐ 5	☐ 4	☐ 3	☐ 2	☐ 1
AN7	I am able to do my usual activities	☐ 5	☐ 4	☐ 3	☐ 2	☐ 1
AN8	I need to sleep during the day	☐ 1	☐ 2	☐ 3	☐ 4	☐ 5
AN12	I am too tired to eat	☐ 1	☐ 2	☐ 3	☐ 4	☐ 5
AN14	I need help doing my usual activities	☐ 1	☐ 2	☐ 3	☐ 4	☐ 5
AN15	I am frustrated by being too tired to do the things I want to do	☐ 1	☐ 2	☐ 3	☐ 4	☐ 5
AN16	I have to limit my social activity because I am tired	☐ 1	☐ 2	☐ 3	☐ 4	☐ 5

20 February 2017
© 2008-2017 PROMIS Health Organization and PROMIS Cooperative Group Page 1 of 1

Short Form Dyspnea Functional Limitations:

PROMIS Short Form v1.0 - Dyspnea Functional Limitations - 10a 02Sept2016
http://www.healthmeasures.net/index.php?option=com_instruments&view=measure&id=774&Itemid=992

Dyspnea Functional Limitations - Short Form 10a

Please respond to each question or statement by marking one box per row.

	**Considering your shortness of breath <u>over the past 7 days</u>, rate the amount of difficulty you had when doing the following activities…**	**No difficulty**	**A little difficulty**	**Some difficulty**	**Much difficulty**	**I did not do this in the past 7 days**
DYSFL001	Dressing yourself without help	☐ 0	☐ 1	☐ 2	☐ 3	☐ X
DYSFL002	Walking 50 steps/paces on flat ground at a normal speed without stopping	☐ 0	☐ 1	☐ 2	☐ 3	☐ X
DYSFL003	Walking up 20 stairs (2 flights) without stopping	☐ 0	☐ 1	☐ 2	☐ 3	☐ X
DYSFL004	Preparing meals	☐ 0	☐ 1	☐ 2	☐ 3	☐ X
DYSFL005	Washing dishes..	☐ 0	☐ 1	☐ 2	☐ 3	☐ X
DYSFL006	Sweeping or mopping.............................	☐ 0	☐ 1	☐ 2	☐ 3	☐ X
DYSFL007	Making a bed...	☐ 0	☐ 1	☐ 2	☐ 3	☐ X
DYSFL008	Lifting something weighing 10-20 lbs (about 4.5-9kg, like a large bag of groceries)..	☐ 0	☐ 1	☐ 2	☐ 3	☐ X
DYSFL009	Carrying something weighing 10-20 lbs (about 4.5-9kg, like a large bag of groceries) from one room to another........	☐ 0	☐ 1	☐ 2	☐ 3	☐ X
DYSFL010	Walking (faster than your usual speed) for ½ mile (almost 1 km) without stopping	☐ 0	☐ 1	☐ 2	☐ 3	☐ X

© 2010-2016 PROMIS Health Organization and PROMIS Cooperative Group

PRIME-AIR Study

Research Consent Form

Subject Identification

Certificate of Confidentiality Template
Version Date: January 2019

Protocol Title: PRIME-AIR STUDY- An Anesthesia-Centered Bundle to Reduce Postoperative Pulmonary Complications<br><br>Principal Investigator: Marcos F Vidal Melo<br><br>Site Principal Investigator: University of Colorado – Ana Fernandez-Bustamante, MD, PhD<br><br>Description of Subject Population: Adult patients planning to undergo open abdominal surgery

About this consent form

Please read this form carefully. It tells you important information about a research study. A member of our research team will also talk to you about taking part in this research study. People who agree to take part in research studies are called "subjects". This term will be used throughout this consent form.

Partners HealthCare System is made up of Partners hospitals, health care providers, and researchers. In the rest of this consent form, we refer to the Partners system simply as "Partners".

If you decide to take part in this research study, you must sign this form to show that you want to take part. We will give you a signed copy of this form to keep.

Key Information

Taking part in this research study is up to you. You can decide not to take part. If you decide to take part now, you can change your mind and drop out later. Your decision won't change the medical care you get at our hospitals now or in the future.

The following key information is to help you decide whether or not to take part in this research study. We have included more details about the research in the Detailed Information section that follows the key information.

Why is this research study being done?

We want to learn if different ways of doing routine anesthesia and patient care before, during and after their surgery can reduce breathing problems after abdominal surgery.

How long will you take part in this research study?

The total time of the study is 3 months. A member of the study team will visit you daily while you are in the hospital up to 7 days after your surgery. After you leave the hospital, study staff will make 3 short follow up phone calls to you.

What will happen if you take part in this research study?

If you decide to join this study, your abdominal surgery will **not** change in any way. The anesthesia medications and techniques that you will receive in this study are generally used in clinical care. However, as part of the study, they will be in a study specific regimen. Your doctors will have the final say in deciding what they believe is best for you during your surgery.

We will assign you by chance (like a coin toss) to one of two groups: the Usual Care group or the Intervention group. Both groups will receive anesthesia care according to current clinical standards. If you are assigned to the Usual Care group, you will be cared for as usual by the team assigned to your surgery and anesthesia. If you are assigned to the Intervention group, during surgery, the medications to keep your muscles relaxed will be guided by the study protocol using a clinical device to check the level of your muscle relaxation. Also, the breathing assist machine will be adjusted using a method in which doctors try to find the pressure that makes your lungs expand the best during surgery.

Both groups may receive educational materials before the surgery, which may differ depending on what group you are in. You will be visited and called daily by study staff after the surgery while in the hospital. Either group may be asked to perform specific breathing exercises and move out of bed while in the hospital.

Why might you choose to take part in this study?

You may not benefit from taking part in this research study. It is possible that your breathing problems are reduced if you take part in this study, but we cannot guarantee this. What we learn from this study may help future patients who will receive abdominal surgery and may be at risk of developing lung problems.

Why might you choose NOT to take part in this study?

Taking part in this research study has some risks and requirements that you should consider carefully.

Because you are having elective abdominal surgery, several important risks are present even if you do not participate in the study. These risks will be discussed with you by your doctors. Some of the risks of the study include the breathing problems, discomfort with blood draws, privacy concerns, or that you may find some of the questions in the health questionnaire sensitive or distressing.

A detailed description of side effects, risks, and possible discomforts can be found later in this consent form in the section called "What are the risks and possible discomforts from being in this research study?".

What other treatments or procedures are available for your condition?

Use of a breathing assist machine and muscle relaxants are part of the regular care during abdominal surgery. As a component of your regular medical care, it will be done whether or not you take part in the research. Different anesthesiologists have different preferences on the pressure settings they use during artificial breathing and doses of muscle relaxants. To date, there is no established guideline for the ranges of pressures that are used. Breathing exercises and encouraging you to get out of bed and walk after surgery are used for clinical care during recovery from surgery.

If you have questions or concerns about this research study, whom can you call?

You can call us with your questions or concerns. Our telephone numbers are listed below. Ask questions as often as you want.

Marcos F Vidal Melo, MD is the person in charge of this research study, and at the MGH site. You can call him at the 617-726-4654 Monday-Friday from 9am to 5pm. You can also page him through the hospital page system at 617-726-6000 on the off time or on the weekends with questions about this research study. ~~You can also call Maureen English at the MGH at 617-726-6434 Monday-Friday from 7am-3pm with questions about this research study or about the scheduling of study visits or calls.~~ For the University of Colorado, Dr. Ana Fernandez-Bustamante is the person in charge. You can call her at 720-848-3316 Monday-Friday from 9am to 5pm. You can also call Nick Naughton at the University of Colorado at 303-724-0833

Monday-Friday from 9am-5pm with questions about this research study or about the scheduling of study visits or calls.

If you want to speak with someone **not** directly involved in this research study, please contact the Partners Human Research Committee office. You can call them at 857-282-1900.

You can talk to them about:

- Your rights as a research subject
- Your concerns about the research
- A complaint about the research
- Any pressure to take part in, or to continue in the research study

Detailed Information

A description of this clinical trial will be available on *http://www.ClinicalTrials.gov,* as required by U.S. Law. This Web site will not include information that can identify you. At most, the Web site will include a summary of the results. You can search this Web site at any time.

Why is this research study being done?

We are doing this study to find out if we can reduce breathing problems after surgery by doing a combination of things that try to keep your lungs well expanded during and after surgery, compared to the usual care.

Everything we will do in the study (how we use the breathing machine and muscle relaxation during surgery and care after surgery) has been used in patients before. We would like to find out if doing this specific set of steps helps to decrease the risk for lung problems during and after surgery.

For the type of surgery that you will have, patients are given general anesthesia. General anesthesia is medication given to prevent pain and awareness during surgery. While under general anesthesia, medications are given to relax your muscles to allow an anesthesia machine to breathe for you. This means you will breathe with the help of a breathing assist machine that is connected to a tube placed in your windpipe.

Breathing assist machines push air into the lungs at different levels of pressure. The way to control the pressure is by changing the settings on the machines, like the volume control on a radio. Recently, doctors have found that some settings used in the breathing machines may cause less harm to the lungs than others. In this study we want to find the best settings to be applied by the breathing assist machine during your surgery. We will compare a method that uses a protocol to expand the lungs in a certain way during surgery, with the condition when no specific guidance is given to the anesthesia team. This method has been used in patients before, and we would like to find out if it causes fewer lung problems during and after surgery.

During abdominal surgery, muscle relaxants are usually given to relax the muscles of your belly and make the surgery easier. Before you wake up from anesthesia, medications are sometimes given to reverse the effect of those muscle relaxants and return your normal muscle tone. The doses of all these medications can change the risk for breathing problems after surgery. In this study, we will follow a protocol to give you these doses. This method has been used in patients before, and we would like to find out if it causes fewer lung problems during and after surgery.

You may be asked to do breathing exercises and start moving out of bed after surgery. All these are part of the routine care after surgery.

Who will take part in this research?

This will be a study in multiple medical centers in the US for a total of 750 patients. We expect to enroll a total of 53subjects at the University of Colorado.

The National Institutes of Health, National Heart, Lung and Blood Institute is paying for the study to be done.

What will happen in this research study?

If you choose to take part in this study, we will ask you to sign this consent form before we do any study procedures.

It is important to know that if you agree to take part in this study, your abdominal surgery will **not** change in any way. The anesthesia medications and techniques that you will receive in this study are generally used in clinical care. However, as part of the study, they will be in a study specific regimen. Your doctors will have final say in deciding what they believe is best for you during your surgery. You will be asked to sign a separate consent form for your abdominal surgery.

Assignment to Study Group
We will assign you by chance (like a coin toss) to the Usual Care group or the Intervention group. You and the study doctor cannot choose your study group. You will have an equal chance of being assigned to either group**.** You won’t know which study group you are in, but the study doctor will know.

Usual care group: You will receive medications for general anesthesia that are used for routine care, including medications to keep your muscles relaxed during surgery and return them to normal at the end of surgery. You will receive breathing assistance from the breathing assist machine using the pressure settings typical for your type of surgery deemed to be appropriate by your anesthesia team.

Intervention group: You will receive medications for general anesthesia that are used for routine care. Doses of medications to keep your muscles relaxed, and returned to normal at the end of surgery will be determined by the study protocol and guided by a device to check the level (or depth) of muscle relaxation. You will receive breathing assistance from the breathing assist

machine in which study doctors try to find the pressures that make your lungs expand the best during surgery. This is done by adjusting the pressures settings to allow your lungs to expand with the least amount of change in pressure during breathing.

Both groups: You may receive educational materials before the surgery, which may differ depending on what group you are in. You will be visited and called daily by study staff after the surgery while in the hospital. Either group may be asked to perform specific breathing exercises and move out of bed while in the hospital.

As part of this study, we will review your medical record and collect basic information about your medical history and ask you to fill out two one-page questionnaires about your level of shortness of breath and fatigue before your surgery.

We will also draw three blood samples: before your surgery, at the end of surgery and the day after your surgery. We will draw about 2 teaspoons of blood each time and a total of 6 teaspoons of blood during the entire course of this research study. We will analyze these blood samples for signs of lung injury and inflammation as part of this study.

After your surgery, we will give you follow up phone calls, or visit you, if you are still at the hospital, 7, 30 and day 90 days after your surgery. The phone calls will be about 15-20 minutes with questions about your lungs and health after the surgery.

You can give us permission to get this information from a representative (for example, your wife/husband). If we cannot get in contact with you or this representative, investigators will try to get information needed for this study follow-up through your medical records, community physicians or other hospitals, by telephone and/or in writing.

Review of Medical Records from Hospital Admissions or Emergency Department Visits.
Partners has an electronic system that lets your study doctors know if you are admitted to a Partners Hospital, or if you visit a Partners Emergency Department. We want to make sure the study doctors know about any possible problems or side effects you experience while you are taking part in the study.

Study Information Included in Your Electronic Medical Records.
A notation that you are taking part in this research study may be made in your electronic medical record. Information from the research that relates to your general medical care may be included in the record (for example: list of allergies, results of standard blood tests done at the hospital labs.)

Optional storing of blood samples in a biorepository:

After the study ends, we would like to store some of your blood samples in the National Heart Lung and Blood Institute (NHLBI) Biologic Specimen and Data Repository (BioLINCC). After transfer to BioLINCC, other investigators outside of this study may request the blood samples. If the NIH/NHLBI grants them permission, investigators may use these blood samples to answer research questions related to lung conditions, anesthesia, surgery, and perioperative medicine.

You can choose **not** to have your blood samples stored in the BioLINCC repository. You can still take part in the research study whether or not you give permission for sharing and use of the blood samples and health information for the BioLINCC.

How are my samples and health information stored in the blood bank?
Staff at the PRIME-AIR biorepository in the University of Colorado will receive your samples and health information de-identified. A code number to your samples and health information will be assigned prior to shipment. Your name, medical record number, or other information that identifies you will not be stored with your samples or health information. The key to the code that connects your name to your samples and information will be stored securely in a separate file at the facility where your surgery took place. This information can only be accessed by approved study staff at this facility.
Once the study is finished, samples will be submitted to the NHLBI BioLINCC. If BioLINCC approves the samples, samples will be sent to their repository. The code number that identifies you will not be provided to BioLINCC. Therefore, your samples cannot be withdrawn.

Which researchers can use my samples and what information about me can they have?
In order to allow researchers to share research results, agencies such as the National Institutes of Health (NIH) have developed secure banks, including the **NHLBI Biologic Specimen and Data Repository (BioLINCC**) that collect and store research samples and health-related information and data, including genetic studies. BioLINCC or other NIH central banks may share these samples or information with other qualified and approved researchers to do more studies. Results or samples given to the central banks will not contain information that directly identifies you. There are many safeguards in place at these banks to protect your privacy.

How long will the bank keep my blood samples and information?
We may store your blood samples and information indefinitely.

Can I stop allowing your samples and information to be stored and used for research?
You can withdraw your blood samples while this study is active and your samples are in the PRIME-AIR biorepository. Once biospecimens go into the NHLBI BioLINCC repository, there is no option for you to withdraw consent for their use.

What are the risks of the BioLINCC repository?
The main risk of storing your blood samples and health information into the BioLINCC repository is a potential loss of privacy. By coding your samples and health information we reduce that risk.

You can still take part in the research study whether or not you give permission for the storage, use, and sharing of the samples and health information for other research through the BioLINCC.

Do you agree to let us store and use your blood samples linked to your health information for future research projects at the BioLINCC or other NIH-approved biorepositories?

☐ YES ☐ NO Initials _______________

How may we use and share your samples and health information for other research?

The samples and information we collect in this study may help advance other research. If you join this study, we will remove all information that identifies you (for example your name, medical record number, and date of birth) and use these de-identified samples and data in other research. It won't be possible to link the information or samples back to you. Information and/or samples may be shared with investigators at our hospitals, at other academic institutions, or at for-profit, commercial entities. You will not be asked to provide additional informed consent for these uses.

Will you get the results of this research study?

Your doctors will get information that affects your clinical care, for instance, if you particularly benefit from lung expansion. You and your doctor should not expect to get information about the general results of the research study or the results of your individual participation in the research study. We and the researchers involved in this study will study samples and information from many people. It could take many years before anyone knows the results of the study.

What are the risks and possible discomforts from being in this research study?

How your surgery will be performed will be decided by your treating physicians and is completely unrelated to your taking part in this study. Your doctors will discuss with you the

risks of your surgery and you'll be asked to sign a separate consent form for your surgery and anesthesia.

There are a few risks associated with taking part in this study. It is important to understand that these risks are an unavoidable part of the use of a breathing assist machine and muscle relaxants during anesthesia for abdominal surgery even if you do not participate in the study.

- **Lung problems related to artificial breathing and abdominal surgery:** Any person who goes under general anesthesia and has a breathing tube placed into their windpipe has a risk of lung problems. Doctors use forced air flow through the tube in your airways to help you breathe and this may rarely cause either inflammation or air leaks in your lungs if the pressure is too high. Problems because of low lung volumes (less air than usual inside the lungs) are common during and after general anesthesia and abdominal surgery. Low lung volumes may increase the risk of lung problems after surgery such as pneumonia. The use of the low-pressure ranges with the use of a breathing assist machine may also be associated with low lung volumes.

- **Risk of muscle weakness:** During anesthesia and abdominal surgery, your muscles are relaxed during surgery. This is routinely done with medications. At the end of surgery, this muscle relaxation is reversed before you wake up from general anesthesia. In case there is any muscle weakness after surgery, you could have problems with your breathing, including shallow breathing, low oxygen levels and higher risk of infection in your lungs (pneumonia).

- **Low blood pressure:** During anesthesia and surgery, anyone can develop low blood pressure. The use of the high-pressure ranges for artificial ventilation can increase the tendency for low blood pressure, which produces the need for a person to receive fluids and medications to help control the blood pressure. Your blood pressure is measured frequently during surgery (at least every 3 minutes) so that blood pressure can be promptly corrected if low.

- **Blood drawing risks:** You may have a bruise (a black and blue mark) or pain where we take the blood samples. There is also a small risk of feeling lightheaded, fainting, or infection. We will draw blood from the IV catheter that is placed in your arm as part of your routine care for your surgery whenever possible. Once the IV catheter is in place, you should not have any discomfort from having blood drawn.

- **Risk of pain and lightheadedness** from breathing exercises and getting out of bed (and walking) after surgery. These are part of routine care to improve your breathing and facilitate

your recovery after surgery. They can make your pain worse, and they can make you feel lightheaded temporarily if you try to do it too fast

- There is the risk that you might find some of the questions in the **health questionnaires** sensitive or distressing. You are free to decline to answer any particular question you do not wish to answer for any reason.

- **Risk of loss of confidentiality:** There is a risk that people outside of the research team will see your research information. We will do all that we can to protect your information, but it cannot be completely guaranteed.

The anesthetics and monitoring used for this study are used routinely and extensively in everyday clinical practice. You should not experience any discomfort because you will be under general anesthesia during your surgery.

What are the possible benefits from being in this research study?

You may not benefit from taking part in this study. It is possible that your breathing problems are reduced if you take part in this study, but we cannot guarantee this. What we learn from this study may help future patients who are undergoing abdominal surgery and may be at high risk of developing lung problems.

What other treatments or procedures are available for your condition?

If you choose not to take part in this study, your care will be given at the discretion of your anesthesia and surgery team. This is the same as when you are assigned to the usual care group in the study.

Can you still get medical care within Partners if you don't take part in this research study, or if you stop taking part?

Yes. Your decision won't change the medical care you get within Partners now or in the future. There will be no penalty, and you won't lose any benefits you receive now or have a right to receive.

We will tell you if we learn new information that could make you change your mind about taking part in this research study.

What should you do if you want to stop taking part in the study?

If you take part in this research study, and want to drop out, you should tell us. We will make sure that you stop the study safely. We will also talk to you about follow-up care, if needed.

Also, it is possible that we will have to ask you to drop out of the study before you finish it. If this happens, we will tell you why. We will also help arrange other care for you, if needed.

Will you be paid to take part in this research study

You will not be paid for taking part in this research study.

What will you have to pay for if you take part in this research study?

Study funds will pay for certain study-related items and services (special blood tests for markers of lung injury collected before and after surgery) that will be done only for the research. You/your health insurer will be billed for routine costs of surgery and anesthesia that you would receive even if you did not take part in the research. You will be responsible for payment of any deductibles and co-payments required by your insurer for this routine care or other billed care.

If you have any questions about costs to you that may result from taking part in the research, please speak with the study doctors and study staff. If necessary, we will arrange for you to speak with someone in Patient Financial Services about these costs.

What happens if you are injured as a result of taking part in this research study?

We will offer you the care needed to treat any injury that directly results from taking part in this research study. We reserve the right to bill your insurance company or other third parties, if appropriate, for the care you get for the injury. We will try to have these costs paid for, but you may be responsible for some of them. For example, if the care is billed to your insurer, you will be responsible for payment of any deductibles and co-payments required by your insurer.

If you are injured as a direct result of taking part in this research study, we will assist you in obtaining the medical care needed to treat the injury.

Injuries sometimes happen in research even when no one is at fault. There are no plans to pay you or give you other compensation for an injury, should one occur. However, you are not giving up any of your legal rights by signing this form.

If you think you have been injured or have experienced a medical problem as a result of taking part in this research study, tell the person in charge of this study as soon as possible. The researcher's name and phone number are listed in the beginning of this consent form.

If you take part in this research study, how will we protect your privacy?

Federal law requires Partners to protect the privacy of health information and related information that identifies you. We refer to this information as "identifiable information."

In this study, we may collect identifiable information about you from:

- Past, present, and future medical records
- Research procedures, including research office visits, tests, interviews, and questionnaires

Who may see, use, and share your identifiable information and why:

- Partners researchers and staff involved in this study
- The sponsor(s) of the study, and people or groups it hires to help perform this research or to audit the research
- Other researchers and medical centers that are part of this study
- The Partners ethics board or an ethics board outside Partners that oversees the research
- A group that oversees the data (study information) and safety of this study
- Non-research staff within Partners who need identifiable information to do their jobs, such as for treatment, payment (billing), or hospital operations (such as assessing the quality of care or research)
- People or groups that we hire to do certain work for us, such as data storage companies, accreditors, insurers, and lawyers
- Federal agencies (such as the U.S. Department of Health and Human Services (DHHS) and agencies within DHHS like the Food and Drug Administration, the National Institutes of Health, and the Office for Human Research Protections), state agencies, and foreign government bodies that oversee, evaluate, and audit research, which may include inspection of your records

- Public health and safety authorities, if we learn information that could mean harm to you or others (such as to make required reports about communicable diseases or about child or elder abuse)

Certificate of Confidentiality

A federal Certificate of Confidentiality (Certificate) has been issued for this research to add special protection for information and specimens that may identify you. With a Certificate, unless you give permission (such as in this form) and except as described above, the researchers are not allowed to share your identifiable information or identifiable specimens, including for a court order or subpoena.

Certain information from the research will be put into your medical record and will not be covered by the Certificate. This includes records of medical tests or procedures done at the hospitals and clinics, and information that treating health care providers may need to care for you. Please ask your study doctor if you have any questions about what information will be included in your medical record. Other researchers receiving your identifiable information or specimens are expected to comply with the privacy protections of the Certificate. The Certificate does not stop you from voluntarily releasing information about yourself or your participation in this study.

Even with these measures to protect your privacy, once your identifiable information is shared outside Partners, we cannot control all the ways that others use or share it and cannot promise that it will remain completely private.

Because research is an ongoing process, we cannot give you an exact date when we will either destroy or stop using or sharing your identifiable information. Your permission to use and share your identifiable information does not expire.

The results of this research may be published in a medical book or journal, or used to teach others. However, your name or other identifiable information **will not** be used for these purposes without your specific permission.

Your Privacy Rights

You have the right **not** to sign this form that allows us to use and share your identifiable information for research; however, if you don't sign it, you can't take part in this research study.

You have the right to withdraw your permission for us to use or share your identifiable information for this research study. If you want to withdraw your permission, you must notify the person in charge of this research study in writing. Once permission is withdrawn, you cannot continue to take part in the study.

If you withdraw your permission, we will not be able to take back information that has already been used or shared with others, and such information may continue to be used for certain purposes, such as to comply with the law or maintain the reliability of the study.

You have the right to see and get a copy of your identifiable information that is used or shared for treatment or for payment. To ask for this information, please contact the person in charge of this research study. You may only get such information after the research is finished.

Informed Consent and Authorization

Statement of Person Giving Informed Consent and Authorization

- I have read this consent form.
- This research study has been explained to me, including risks and possible benefits (if any), other possible treatments or procedures, and other important things about the study.
- I have had the opportunity to ask questions.
- I understand the information given to me.

Signature of Subject:

I give my consent to take part in this research study and agree to allow my identifiable information to be used and shared as described above.

________________________________ ____________ ____________
Subject Date Time (optional)

Signature of Study Doctor or Person Obtaining Consent:

Statement of Study Doctor or Person Obtaining Consent

- I have explained the research to the study subject.
- I have answered all questions about this research study to the best of my ability.

Study Doctor or Person Obtaining Consent	Date	Time (optional)

Consent of Non-English Speaking Subjects Using the "Short Form" in the Subject's Spoken Language

Statement of Hospital Medical Interpreter

As someone who understands both English and the language spoken by the subject, I interpreted, in the subject's language, the researcher's presentation of the English consent form. The subject was given the opportunity to ask questions.

Hospital Medical Interpreter	Date	Time (optional)

OR

Statement of Other Individual (Non-Interpreter)

As someone who understands both English and the language spoken by the subject, I represent that the English version of the consent form was presented orally to the subject in the subject's own language, and that the subject was given the opportunity to ask questions.

Name	Date	Time (optional)
